# From sex differences to sex inequalities in life expectancy: A cross-country observational benchmarking analysis

**DOI:** 10.1371/journal.pmed.1004828

**Published:** 2025-12-11

**Authors:** Angela Y. Chang, Emily K. Johnson, Sarah Bolongaita, Kent Buse, Sarah J. Hawkes, Omar Karlsson, Felicia M. Knaul, Margaret E. Kruk, Ole F. Norheim, Osondu Ogbuoji, David Watkins, Dean T. Jamison

**Affiliations:** 1 Danish Institute for Advanced Study, University of Southern Denmark, Odense, Denmark; 2 Danish Centre for Health Economics, University of Southern Denmark, Odense, Denmark; 3 Bergen Centre for Ethics and Priority Setting, Department of Global Public Health and Primary Care, University of Bergen, Bergen, Norway; 4 Global 50/50, Cambridge, United Kingdom; 5 Department of Global Population Health, Monash University Malaysia, Kuala Lumpur, Malaysia; 6 Duke University Population Research Institute, Durham, North Carolina, United States of America; 7 Department of Economic History, School of Economics and Management, Lund University, Lund, Sweden; 8 Department of Medicine, David Geffen School of Medicine, University of California, Los Angeles, California, United States of America; 9 Tómatelo a Pecho, A. C., Mexico City, Mexico; 10 Escuela de Medicina y Ciencias de la Salud, Tecnológico de Monterrey, Facultad de Excelencia, Mexico City, Mexico; 11 Jonsson Comprehensive Cancer Center, University of California, Los Angeles, California, United States of America; 12 QuEST Center, Washington University School of Medicine in St. Louis, St. Louis, Missouri, United States of America; 13 Department of Medicine, Washington University School of Medicine in St. Louis, St. Louis, Missouri, United States of America; 14 Department of Global Health and Population, Harvard T. H. Chan School of Public Health, Boston, Massachusetts, United States of America; 15 Centre for Policy Impact in Global Health, Duke University, Durham, North Carolina, United States of America; 16 Department of Population Health, Duke School of Medicine, Duke University, Durham, North Carolina, United States of America; 17 Department of Global Health, University of Washington, Seattle, Washington, United States of America; 18 Institute for Global Health Sciences, University of California, San Francisco, California, United States of America; Washington University School of Medicine, UNITED STATES OF AMERICA

## Abstract

**Background:**

The answer to whether females or males have better health, and which sex is the more disadvantaged, has depended in part on the metric and how the inequality is measured. This study introduces a new method for analyzing and interpreting sex inequalities in health outcomes—defined as the avoidable sex differences in health outcomes—that is systematic and potentially more objective. For this paper, we focus on life expectancy at different ages.

**Methods and findings:**

We introduce the adjusted sex ratio as a measure of sex inequalities and determining sex disadvantage. First, we calculated the sex ratio of life expectancy at ages 0, 5, 15, 35, 50, and 70. To understand what is achievable under favorable conditions, we identified countries in the 5th percentile of the highest life expectancy for each sex and used these values as benchmarks, and calculated the sex ratio of these best-performing countries (“frontier”). We calculated the country- and age-specific adjusted sex ratio by dividing country sex ratios by frontier sex ratios. This assumes that theoretically, under the current risk and healthcare environments, females all over the world have the potential to live up to the life expectancy of the females in the frontier countries, and separately, all males to their male-specific frontier. An adjusted ratio of greater than one indicates male disadvantage, while below one indicates female disadvantage. To avoid overinterpreting small differences, we defined a narrow range around equality (ratio of 1) within which we do not label either sex as disadvantaged. Before adjustment, males in all countries (except two) and at all ages had lower life expectancy than females. After adjustment, between 13% (at age 0) and 33% (at age 70) of the 237 countries shift from male to female disadvantage in life expectancy. More than half of the countries remain male-disadvantaged, indicating that males are generally disadvantaged in terms of life expectancy in most countries, even after our adjustments. India and approximately half of the countries in the Middle East and North Africa, North Atlantic, sub-Saharan Africa, and Western Pacific and Southeast Asia show female disadvantage. The number of countries with female disadvantage rises with age, especially in sub-Saharan Africa and Western Pacific and Southeastern Asia. Central and Eastern Europe show substantial male disadvantage across nearly all ages, even with adjustment. Our frontier selection and buffer range are empirical choices, and other definitions could be equally valid. Although our sex-specific benchmarks use the best-performing countries for each sex, they are not meant to represent purely biological differences, as observed sex gaps in life expectancy may also reflect unmeasured genetic variation, environmental exposures, and their interactions with sex.

**Conclusion:**

This study provides a novel, potentially more objective method for assessing sex inequalities in health outcomes, and presents the trends across countries, age, and time.

## Author summary

**Why was this study done?:**

- Females and males often experience different health outcomes, but it is unclear which sex is more disadvantaged overall. While females tend to live longer, some argue that they suffer more from illness or barriers to healthcare. Others emphasize that males face higher risks of premature death.
- Most studies do not adjust for biological differences or provide a consistent way to compare health disadvantages between sexes.
- We aimed to create a more objective and fair method to measure sex-based inequalities in life expectancy around the world.

**What did the researchers do and find?:**

- We analyzed life expectancy data from 237 countries and territories in 2000 and 2019 and compared differences between females and males across different ages.
- We developed a new method that adjusts for expected biological differences using data from the best-performing countries.
- We found that, while males are generally more disadvantaged, in up to one-third of countries, especially at older ages, females are actually worse off after adjustment. These patterns varied by country, region, age, and over time.

**What do these findings mean?:**

- Our findings challenge the common belief that one sex is always more disadvantaged in health outcomes. Instead, disadvantage, while often real, depends on the country, age group, broader context, and the method applied.
- This new approach provides a better way to understand and monitor sex-based health inequalities. It can help policymakers design more targeted and fair health programs for both sexes.
- We use the highest observed sex-specific life expectancies to identify potential avoidable gaps in life expectancy for each sex, with the aim of estimating biological differences, genetic variations, environmental exposures, and their interactions with sex. This is one approach of setting the frontier; other definitions of the frontier may be equally valid.

## Abstract 3

In a proof-of-concept study, Angela Chang and colleagues defined a metric to assess sex inequalities in health outcomes, using countries with the highest life expectancy as bench marks.

## Introduction

A recent World Economic Forum report introduced the concept of the “women’s health gap,” defined as the additional disease burden women face compared to men [1]. It emphasized the urgency of closing this gap, stating that our “collective future rests on closing the women’s health gap.” In contrast, it is also widely acknowledged that women generally live longer than men across the globe, and this longevity gap is widening in some countries and narrowing in others over time [2]. Hawkes and Buse have critiqued these different perspectives as “gender myths,” highlighting the dilemma in global health debates: some groups stress the disproportionate health burden on women, while others focus on higher mortality rates and risk exposures among men [3]. Are these messages contradicting? When it comes to health outcomes, which sex or genders are the more disadvantaged, if any, and does it even matter?

This study is about the measurement of sex inequalities in health, defined as the differences in health outcomes between females and males that may be due to sex, gender, or a combination of both. Some of these inequalities are avoidable, while others are not due to biological differences. We focus on sex and not gender inequalities in this paper due to limited availability and knowledge about gender-disaggregated data. Given that the majority of surveys, health surveillance, and studies do not define whether they are using sex or gender identity, we use the binary terminology of sex (i.e., male/female) in our data analysis, but apply a lens of gender analysis (including beyond the binary) to explain differences found [4]. There has been increased awareness of sex inequalities in health in recent years, driven in part by researchers advocating for more sex-disaggregated data [2,5,6]. Comparisons of health outcomes can then inform priority setting and resource allocation, particularly towards the disadvantaged group. However, measuring and interpreting health inequalities between sexes presents particular challenges, which may have contributed to the lack of action or disinterest among policymakers. First, health can be measured in various ways, including mortality, morbidity, disease prevalence, access to care, well-being, and functioning [7]. For example, the United Nations Gender Inequality Index includes maternal mortality and adolescent fertility to capture gender inequality in health, which highlights female disadvantage but excludes male and nonreproductive female health [8]. Some health issues predominantly affect females, such as interpersonal violence and access to contraception and abortion services, leading researchers and advocacy groups to conclude that females are generally more disadvantaged. Second, global health initiatives often prioritize issues affecting women and girls. A report reviewed the programmatic priorities of 146 major global health organizations and found that 72 organizations focused only on women’s health, and none focused on men’s health [5]. Third, given that women are generally more disadvantaged in the broader societal context—such as labor force participation, political representation, and unpaid caregiving burden—it is common to assume that women also experience worse health outcomes given their societal positions and social determinants, even though this may not always be the case. Fourth, researchers may interpret sex inequalities at the country level based on their existing knowledge of the context. For example, given India’s well-documented systemic discrimination against females, many conclude that Indian females are disadvantaged, despite their longer life expectancy compared to males [9,10]. Conversely, in Eastern Europe, where high alcohol consumption among males is notorious, higher premature mortality among males leads to conclusions of male disadvantage, often without comprehensive knowledge of the conditions affecting females [11].

In response to these challenges, this study aims to develop a more objective and systematic method for analyzing and interpreting sex inequalities in health outcomes, with a focus on inequalities preventable and amenable to health and societal intervention with current levels of knowledge. For illustration, we use life expectancy, one of the most commonly used health indicators for measuring population health, and one with a clear use case for a sex inequality measure that can take into account biological sex differences.

## Methods

Sex inequalities in health outcomes can be expressed either in ratios (female over male) or differences (female minus male). We used sex ratios as main results and provide the results in differences in S1 Appendix. Our approach has four steps: calculation of raw/unadjusted sex ratios, setting the frontier, calculating the adjusted sex ratios, and setting the buffer.

Step 1: For each year, country, and selected age (ages 0, 5, 15, 35, 50, and 70), we calculated the sex ratio of the life expectancy.

Step 2: To understand what is achievable under favorable conditions, we identified countries in the 5th percentile of the highest life expectancy for each sex and age and used these values as benchmarks. We call these countries as the “frontier,” as they are the best-performing countries in life expectancy. We estimate the frontier sex ratio by taking the ratio of the best-performing females (i.e., highest female life expectancies) and best-performing males. For each sex- and age frontier, the 5th percentile includes 13 best-performing countries. The choice of the 5th percentile as the frontier was tested in sensitivity analyses. We assumed the best-performing females have lived up to their highest survival potential given current social, structural, and health system determinants, and separately, best-performing males (which may come from different countries as best-performing females) have also lived up to their highest survival potential. This approach assumes that the sex gap implied in these settings provides an empirical benchmark for what is achievable, rather than a biological norm. More specifically, let $L_{f,a,i}$ be female life expectancy for age *a* in country *i*, and $L_{m,a,i}$ for males. Let $R_{a,i}=\;\frac{L_{f,a,i}\;}{L_{m,a,i}\;}$ be the sex ratio. We calculated ${R_{a}}^{*}=\;\frac{{L_{f,a}}^{*}}{{L_{m,a}}^{*}\;}$ as the frontier sex ratio at age *a*, where ${L_{f,a}}^{*}$ is the upper 5th percentile of $L_{f,a,i}$ and ${L_{m,a}}^{*}$ is the upper 5th percentile of $L_{m,a,i}$, for each age group *a*. Countries which inform ${L_{f,a}}^{*}$ are not necessarily the same as those used to calculate ${L_{m,a}}^{*}.\;$It is important to emphasize that the countries contributing to the female and male frontiers may differ.

Step 3: We adjusted the country-level sex ratio with the frontier sex ratio as an attempt to calculate the “avoidable” sex gap, assuming that the sex gap in the best-performing countries are currently unavoidable. The adjusted ratio, $N_{a,i}=\;\frac{R_{a,i}\;}{{R_{a}}^{*}\;}$ is our outcome of interest. $N_{a,i}\;$ above one indicates male disadvantage, while $N_{a,i}$ below one indicates female disadvantage. $N_{a,i}$ can be interpreted as the gap between sexes in achieving frontier life expectancy. For example, an adjusted ratio of 1.15 indicates that female progress towards frontier life expectancy is 115% that of male progress at the same age in the same country, suggesting male disadvantage. The adjusted ratios, therefore, aim to capture potentially avoidable sex differences in life expectancy, using a pragmatic standard that aligns with established practices in frontier-based health metrics [12–15].

Step 4: To avoid overinterpreting small differences, we defined a narrow range (called “buffers”) around equality (ratio of 1) within which we do not label either sex as disadvantaged. In cases where life expectancy is high for both sexes or the absolute difference between the two is small, an adjusted ratio may take a value slightly above or below 1.0 even though the actual inequality is negligible. These cases should fall within a buffer, within which we do not classify disadvantage, and which reflects some uncertainty in our frontier sex ratio. We set the upper and lower boundaries of this buffer by calculating the adjusted ratio with a half-year increase in the male and female frontier life expectancies, respectively. This creates an upper boundary at $U=\;\left(\frac{{L_{f}}^{*}+\text{0}.\text{5}}{{L_{m}}^{*}}\;\right)/R^{*}\;$and lower boundary at $L=\;\left(\frac{{L_{f}}^{*}}{{L_{m}}^{*}+\text{0}.\text{5}}\;\right)/R^{*}\;$ for each age (S1 Appendix). We also tested alternative buffer ranges in the sensitivity analyses.

Data on life expectancy from 2000 to 2019 are from the World Population Prospects (WPP) 2024 [16]. We applied the regional categorization from the Lancet Commission on Investing in Health (CIH) 3.0, with China, India, and the United States as their own regions due to their large population sizes [17].

### Sensitivity analyses

The calculation of sex inequalities and classification of disadvantage depends on two parameters: the percentile used to define the frontier for each sex and age (step 2) and the buffer (step 4). Without evidence of the irreducible inequalities in life expectancy, it is not possible to define these parameters objectively. Instead, we empirically identify appropriate thresholds and test the sensitivity of the results to variation in both parameters. We presented figures showing possible frontier percentiles of the top 1%, 10%, and 15% performance on sex-specific life expectancy. As another sensitivity analysis, we removed countries with a population less than 5 million people in 2019 to set the frontier. For the buffer, we demonstrated how three alternative definitions of the buffer would classify sex disadvantage given the distribution of adjusted ratios. First, we took 30% of the data centered around an adjusted ratio of 1. Second, we considered a buffer for the adjusted ratios of 0.99 and 1.01. Finally, we tested an alternative life expectancy-based buffer where upper and lower boundaries are set by calculating the adjusted ratios corresponding to a one-year decrease (instead of a half-year increase in the main results) in male and female life expectancy, respectively. A similar approach was taken in the difference (instead of ratio) space. The results presented in ratios were qualitatively similar to results in differences. We therefore only included the ratios in the paper and present the differences in S1 Appendix.

## Results

### Frontier countries

In 2019, the 5th percentile of female and male life expectancy at birth across countries was 85.6 and 81.3 years, respectively, with the ratio at 1.05. The countries at or above the frontier and the sex ratio for other ages are listed in Table 1. The frontier sex ratio increases with age: 1.06 at age 5, 1.07 at age 15, 1.12 at age 50, and 1.19 at age 70, meaning that the female-male gap is wider at older ages.

**Table 1:** Frontier life expectancies, ratios, and countries included in the frontier by age, 2019.

Age	Frontier life expectancy, female	Frontier life expectancy, male	Frontier sex ratio	Top 5^th^ percentile % countries, female	Top 5^th^ percentile countries, male
0	85.6	81.3	1.05	Andorra, Saint Barthelemy, Spain, Gibraltar, Hong Kong, Japan, Republic of Korea, Macao, Monaco, French Polynesia, Singapore, San Marino	Andorra, United Arab Emirates, Switzerland, Hong Kong, Japan, Liechtenstein, Monaco, Qatar, Singapore, San Marino, Sweden, Holy See
5	81.0	76.6	1.06	Andorra, Saint Barthelemy, Spain, Gibraltar, Hong Kong, Japan, Republic of Korea, Macao, Monaco, French Polynesia, San Marino, Holy See	Andorra, United Arab Emirates, Switzerland, Guernsey, Hong Kong, Japan, Liechtenstein, Monaco, Qatar, Singapore, San Marino, Holy See
15	71.1	66.7	1.07	Andorra, Saint Barthelemy, Spain, Gibraltar, Hong Kong, Japan, Republic of Korea, Macao, Monaco, French Polynesia, San Marino, Holy See	Andorra, United Arab Emirates, Switzerland, Guernsey, Gibraltar, Hong Kong, Liechtenstein, Monaco, Qatar, Singapore, San Marino, Holy See
35	51.3	47.2	1.09	Andorra, Saint Barthelemy, Spain, Gibraltar, Hong Kong, Japan, Republic of Korea, Macao, Monaco, French Polynesia, San Marino, Holy See	Andorra, United Arab Emirates, Australia, Switzerland, Gibraltar, Hong Kong, Iceland, Liechtenstein, Monaco, Qatar, San Marino, Holy See
50	36.8	32.8	1.12	Andorra, Saint Barthelemy, Spain, Gibraltar, Hong Kong, Japan, Republic of Korea, Monaco, French Polynesia, Reunion, San Marino, Holy See	Andorra, United Arab Emirates, Australia, Switzerland, Gibraltar, Hong Kong, Iceland, Japan, Monaco, Qatar, San Marino, Sweden
70	18.8	15.8	1.19	Saint Barthelemy, Spain, France, Gibraltar, Guadeloupe, Hong Kong, Japan, Republic of Korea, Monaco, Puerto Rico, Reunion, San Marino	Andorra, Australia, Canada, Switzerland, France, Gibraltar, Hong Kong, Iceland, Israel, Japan, Monaco, San Marino

### Distribution of unadjusted and adjusted sex ratios across countries

**Fig 1:**
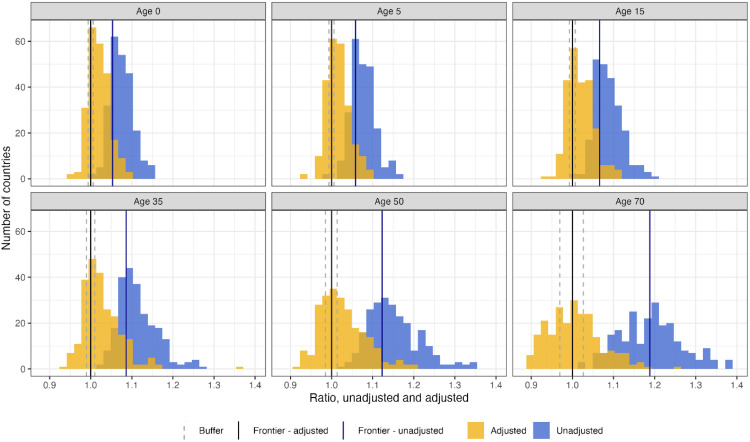
Distribution of ratios and adjusted ratios in 2019, by age. The dotted gray lines indicate the buffer within which we do not classify disadvantage. Upper and lower buffer boundaries are estimates by a 0.5-year life expectancy increase in female and male life expectancy, respectively. Buffer width is larger with age, as a 0.5-year increase in life expectancy is a greater proportion of total life expectancy and therefore has a greater impact on the ratio as age increases and remaining life expectancy decreases.

Fig 1 shows the distribution of the unadjusted and adjusted ratios in 2019 by life expectancy for each age. By design, the distribution of the adjusted ratios shifts towards 1.0 once we remove the frontier sex ratio (a fixed ratio for each age) observed in the highest-performing countries from each country’s original ratio. The degree of shift increases with age, reflecting a larger sex gap at older ages. Table 2 displays the count of countries by the disadvantage implied by the original sex ratios and by the adjusted sex ratios. Without any adjustment, only Nigeria and Togo at age 5 had lower female life expectancy than males. With our adjustment, for life expectancy at birth (age 0), despite females having higher life expectancy in all countries, 31 countries (13%) switched to female disadvantage after adjustment. The number of countries that are categorized as female disadvantage increases with age group, with the highest number of countries, 78 countries (33%), at age 70. The population-weighted distribution of the sex ratios is presented in S1 Appendix.

**Table 2:** Number and proportion of countries classified as female disadvantage, male disadvantage, or no disadvantage before and after adjustment, by life expectancy at different ages, 2019.

	Original sex ratio			Adjusted sex ratio		
	(A) Female disadvantage	(B) Male disadvantage	(C) No disadvantage	(A) Female disadvantage	(B) Male disadvantage	(C) No disadvantage
Birth	0 (0%)	237 (100%)	0 (0%)	31 (13%)	162 (68%)	44 (19%)
Age 5	2 (1%)	235 (99%)	0 (0%)	48 (20%)	141 (59%)	48 (20%)
Age 15	0 (0%)	237 (100%)	0 (0%)	44 (19%)	144 (61%)	49 (21%)
Age 35	0 (0%)	237 (100%)	0 (0%)	45 (19%)	135 (57%)	57 (24%)
Age 50	0 (0%)	237 (100%)	0 (0%)	61 (26%)	125 (53%)	51 (22%)
Age 70	0 (0%)	237 (100%)	0 (0%)	78 (33%)	79 (33%)	80 (34%)

**Fig 2:**
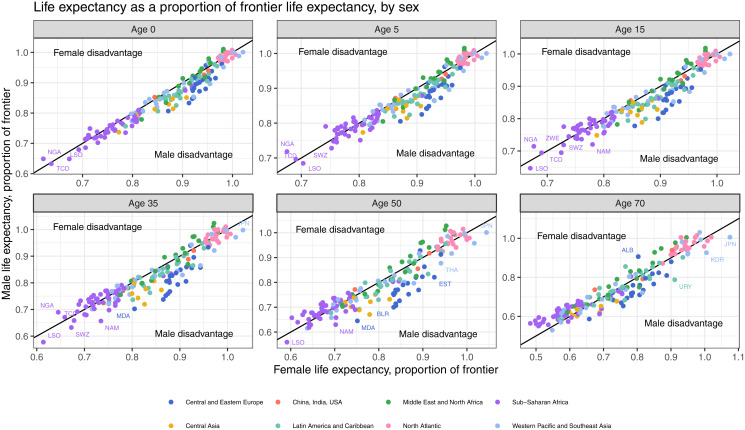
Life expectancy as a proportion of frontier life expectancy for 2019, by sex and life expectancy at different ages. The shaded gray area represents the buffer, within which we do not classify disadvantage. Upper and lower buffer boundaries are estimates by a 0.5-year life expectancy increase in female and male life expectancy, respectively.

The distribution of adjusted sex ratios is presented in Fig 2. We present the adjust ratios by first showing the sex-specific life expectancy against the frontier life expectancy (females in the x-axis and males in the y-axis). Countries on the diagonal black line have adjusted ratios of 1.0, suggesting no sex inequality; countries on the left of the diagonal line have ratios below 1.0, suggesting female disadvantage, and countries on the right side have ratios above 1.0, suggesting male disadvantage. For example, using life expectancy at birth, in Nigeria actual female life expectancy is 62.2% (53.2 years/85.6 years) of the female frontier life expectancy, and 64.9% (52.8 years/81.3 years) of the male frontier life expectancy. The adjust sex ratio is 0.96, interpreted as 4% disadvantage in females and placing Nigeria as female disadvantage. Our interpretation is that life expectancy at birth of Nigerian females is further away from the frontier females than Nigerian males are from the frontier males. Generally, we see more countries classified as male disadvantaged, but with increase in age, more countries move towards female disadvantage. Some countries, especially in Central and Eastern Europe, fall far below the line, suggesting strong male disadvantage. There is no clear relationship between the level of sex inequality and the average life expectancy across all countries, and consistent male disadvantage is present in both low- and high-longevity countries.

### Adjusted sex ratios by region

**Fig 3:**
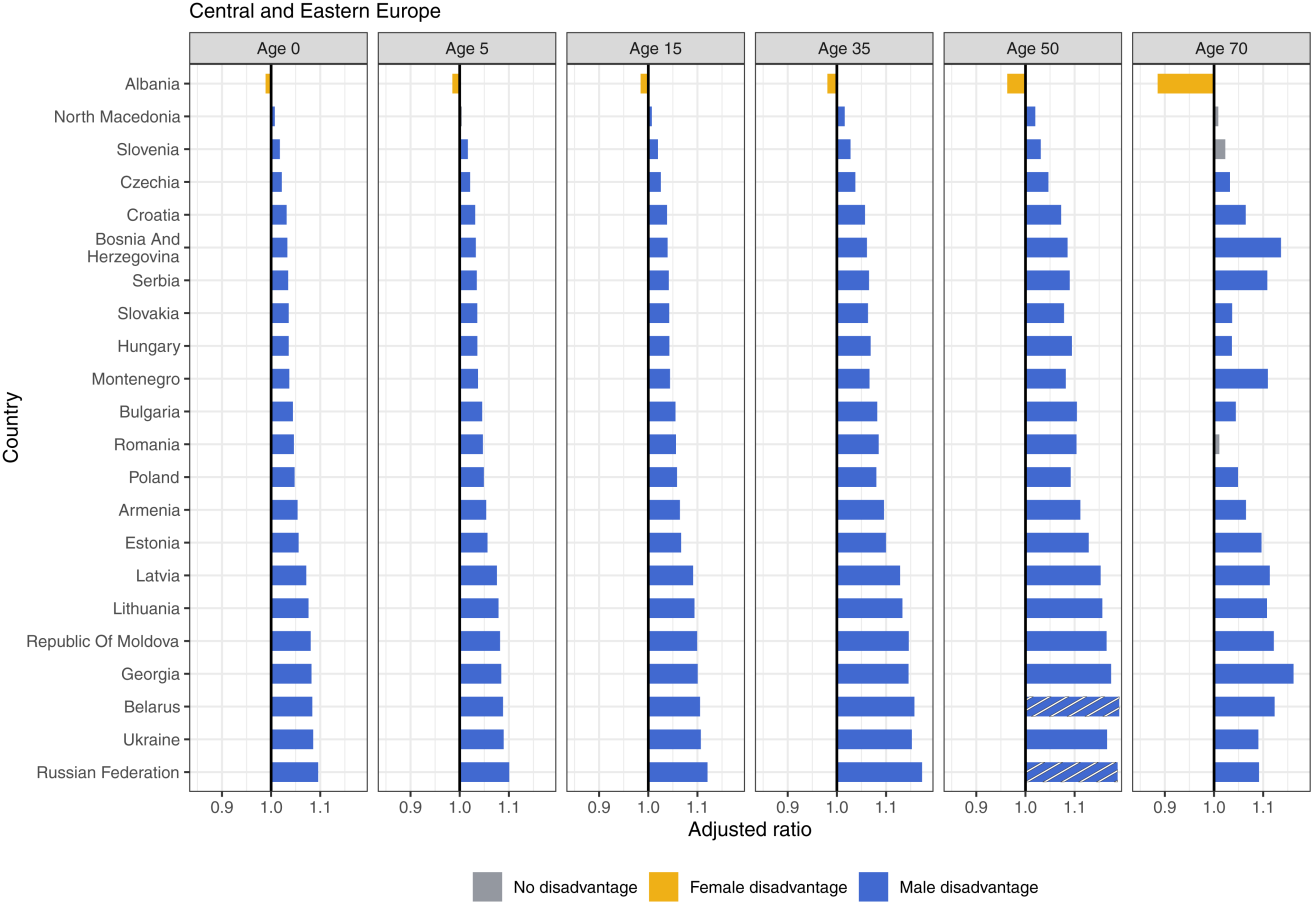
Adjusted ratios for Central and Eastern Europe, 2019, by age. Axes on the plots below are constrained to ratios between 0.85 and 1.18 for readability purposes. Countries with ratios above or below these boundaries are indicated by the striped bars. The full table of values for each country is included in S1 Appendix.

**Fig 4:**
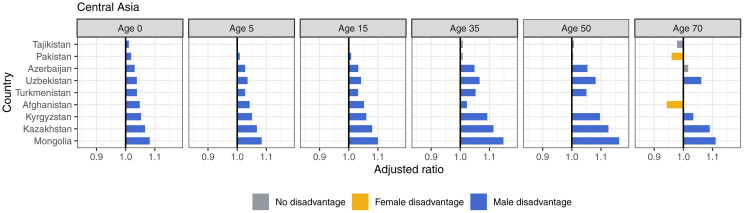
Adjusted ratios for Central Asia, 2019, by age.

**Fig 5:**
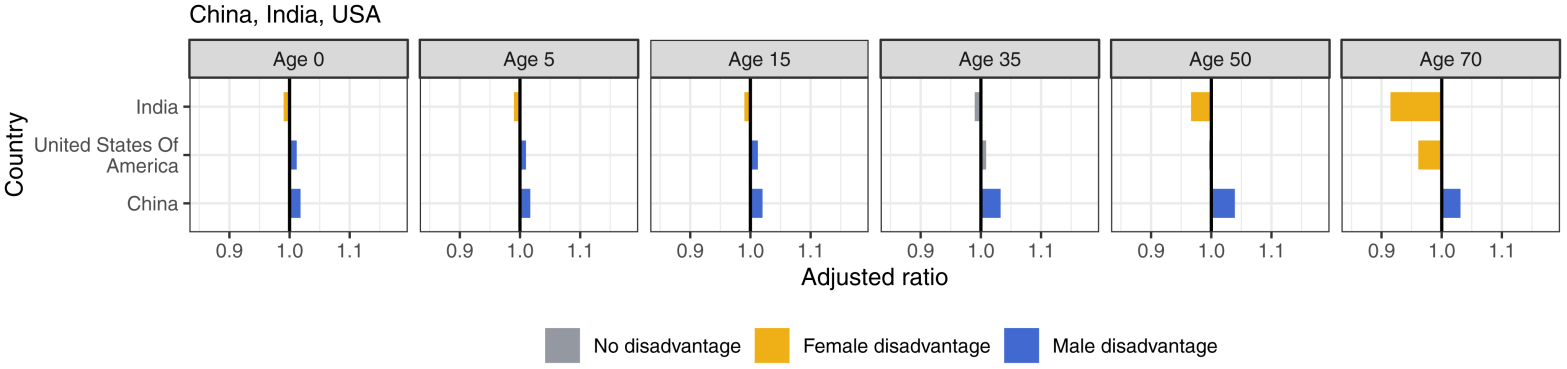
Adjusted ratios for China, India, and United States, 2019, by age.

We estimated the adjusted ratio for each country by the CIH regions. In Central and Eastern Europe, there was consistent male disadvantage across most ages, with the exception of Albania, where female disadvantage persists (Fig 3). Male disadvantage reached the highest levels in the region at 1.19 (i.e., 19% greater advantage in females than males) and 1.18 at age 50 in Belarus and Russia. Likewise, in Central Asia, male disadvantage started in all countries at birth and perpetuated throughout the life span. Female disadvantage in this region existed in Pakistan and Afghanistan, both at age 70 (Fig 4). India experienced female disadvantage at all ages except age 35 (Fig 5).

**Fig 6:**
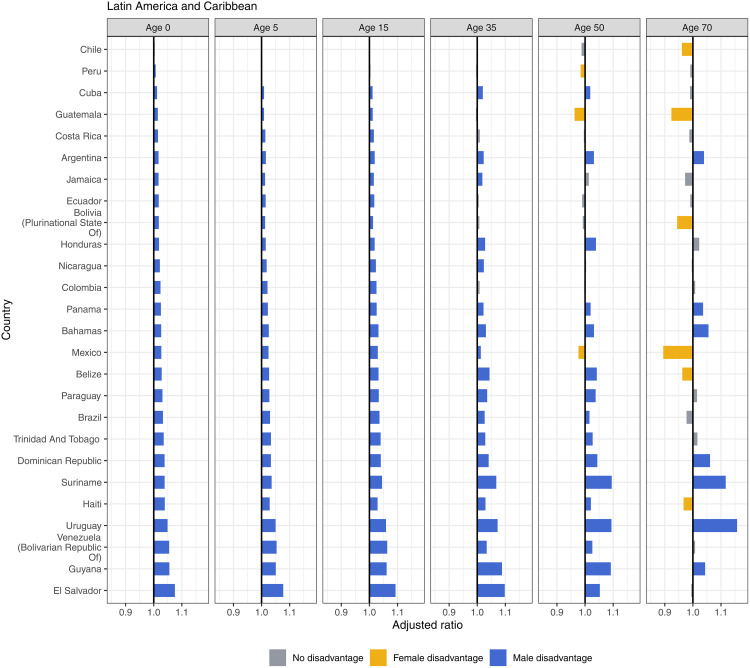
Adjusted ratios for Latin America and Caribbean, 2019, by age.

**Fig 7:**
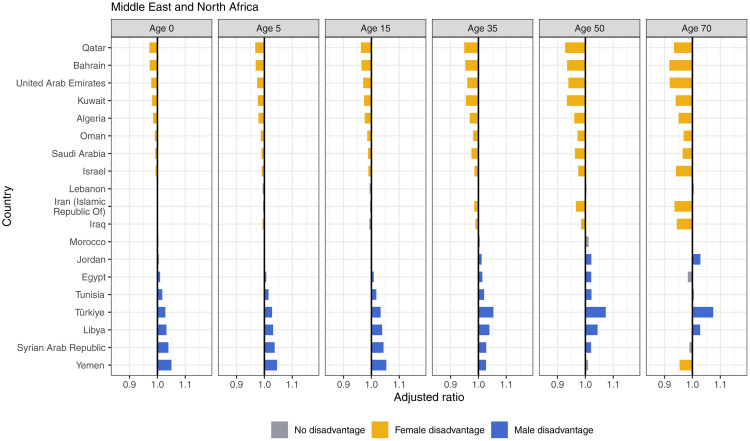
Adjusted ratios for Middle East and North Africa, 2019, by age.

Most countries in Latin America and the Caribbean showed consistent male disadvantage and rising with age (Fig 6). Mexico observed notable male disadvantages at birth through age 35, but then saw female disadvantages at age 70. Guatemala and Peru also experienced female disadvantage at age 50, and Chile, Guatemala, Bolivia, Belize, and Haiti experienced female disadvantage at age 70. In the Middle East and North Africa, eight countries experienced female disadvantage in life expectancy at birth (Fig 7). Eight countries had female disadvantage across the life span. Conversely, Yemen, Syrian Arab Republic, Libya, and Türkiye experienced male disadvantage in life expectancy at birth and in older ages, though only Türkiye and Libya sustained male disadvantage to life expectancy at age 70.

**Fig 8:**
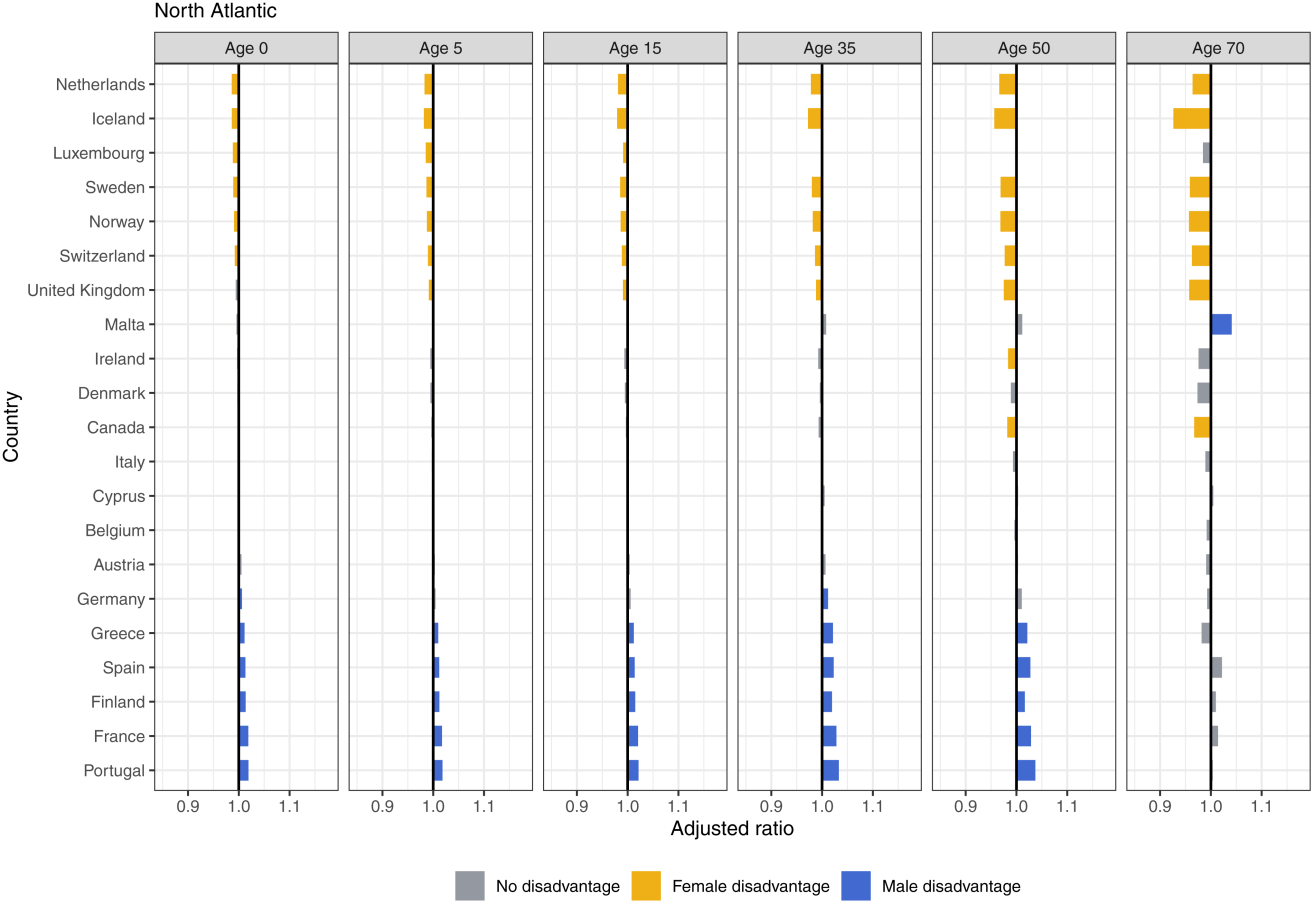
Adjusted ratios for North Atlantic, 2019, by age.

The magnitude of sex inequalities at any age in the North Atlantic is smaller than in other regions, with the exception of the female disadvantage in older ages in Iceland (Fig 8). The Netherlands, Iceland, Sweden, Norway, and Switzerland observed small female disadvantage in all ages. Five countries did not experience sex disadvantage at any age: Denmark, Italy, Cyprus, Belgium, and Austria. We saw small male disadvantages in Portugal, France, Finland, Spain, and Greece. The United States saw small male disadvantages at birth through age 15, then saw no disadvantage until age 70, where females experienced a minor disadvantage (Fig 5).

**Fig 9:**
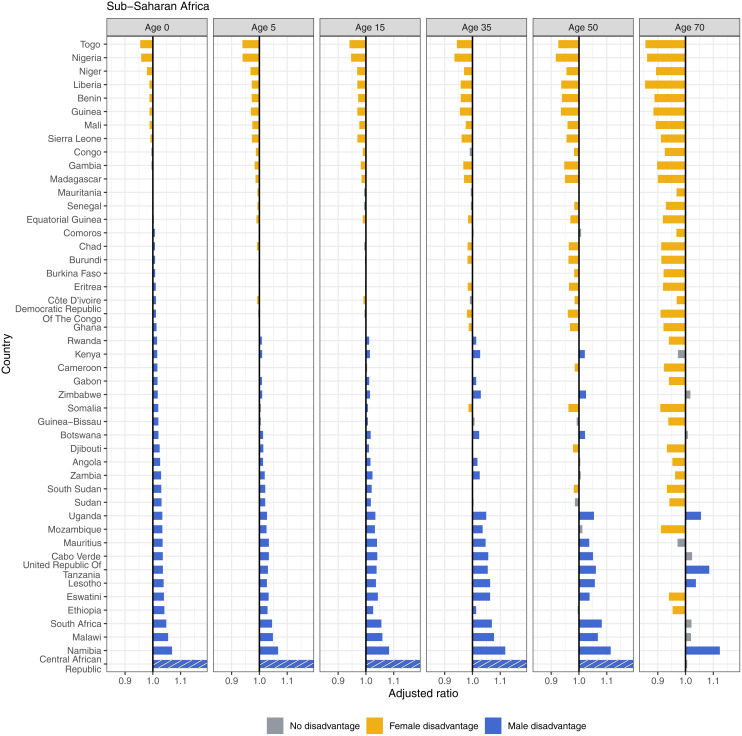
Adjusted ratios for sub-Saharan Africa, 2019, by age.

Sub-Saharan Africa had the highest and lowest ratios in any region, indicating the highest levels of male and female disadvantage globally (Fig 9). Togo, Nigeria, Niger, Liberia, Benin, Guinea, Mali, and Sierra Leone showed female disadvantages in all ages. Nigeria, for example, had a ratio of 0.96 at birth (i.e., a 4% advantage in males) and 0.86 (14% advantage in males) at age 70. Four additional countries showed female disadvantage in all except one age group. Males in the Central African Republic experience the greatest magnitude of sex disadvantage of any country, reaching 1.40 at birth, 1.44 at age 5, 1.65 at age 15, and 1.37 at age 35. Namibia, Tanzania, Lesotho, and Uganda consistently showed male disadvantage across the life span, while nine additional countries showed male disadvantage in all but one age group. In this region, eight countries had female disadvantage at birth, and 33 countries had male disadvantage, but the pattern flipped to 35 countries with female disadvantage and 4 with male disadvantage at age 70.

**Fig 10:**
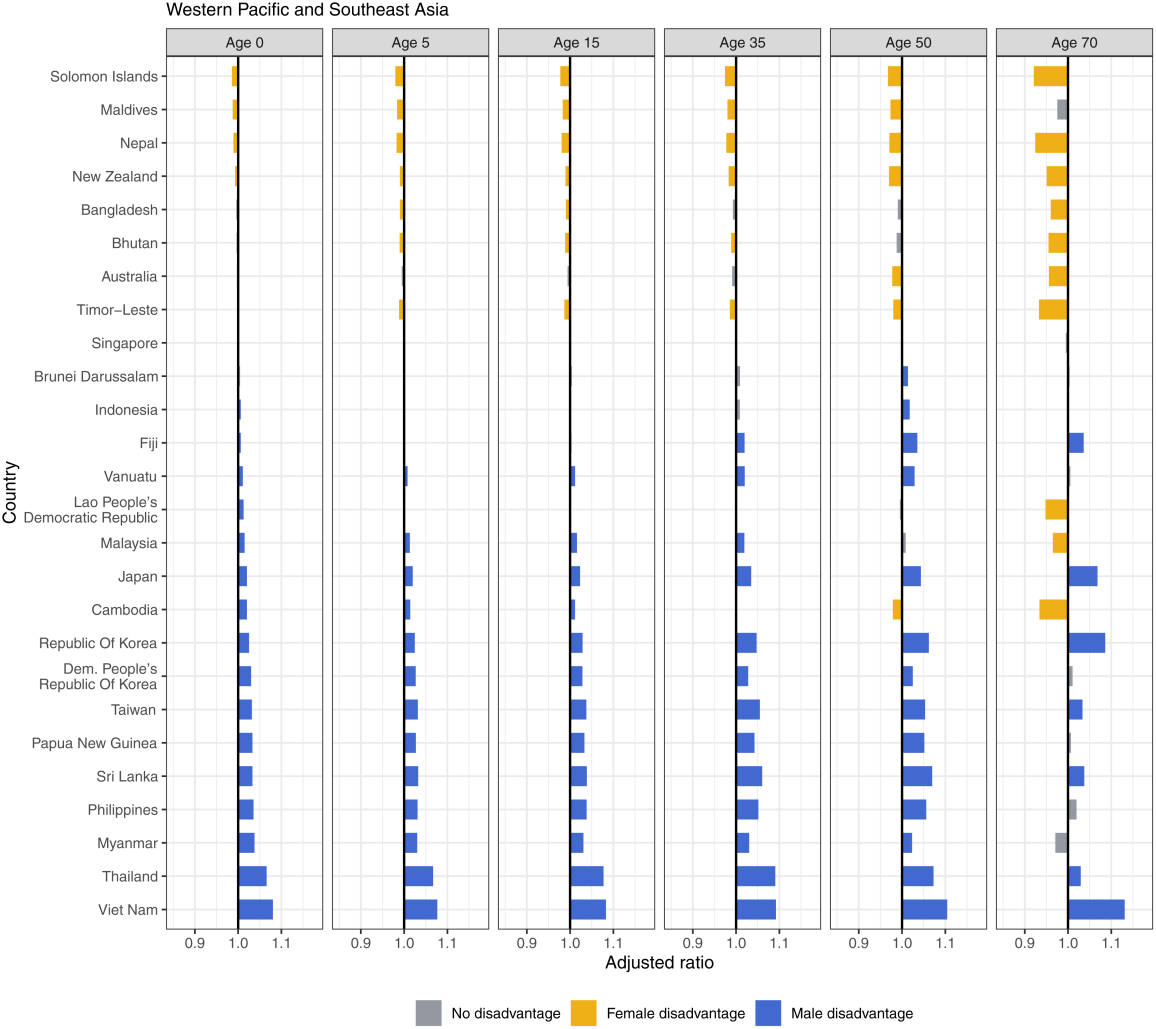
Adjusted ratios for Western Pacific and Southeast Asia, 2019, by age.

In Western Pacific and Southeast Asia, Viet Nam, Thailand, and Sri Lanka had male disadvantage, which stands out in the region, at 1.08 and 1.07 at birth, respectively, and consistently high until age 70, with a ratio of 1.13 in Viet Nam (Fig 10). Despite their high overall life expectancies, Japan, Taiwan, and Republic of Korea consistently showed male disadvantage across the life span. Other countries with mostly male disadvantaged include Myanmar, Philippines, Papua New Guinea, and Democratic People’s Republic of Korea. Solomon Islands, Nepal, and New Zealand showed female disadvantages in life expectancy at all ages. China experienced small but consistent male disadvantages across the life span. India showed female disadvantage across most ages (Fig 5).

**Fig 11:**
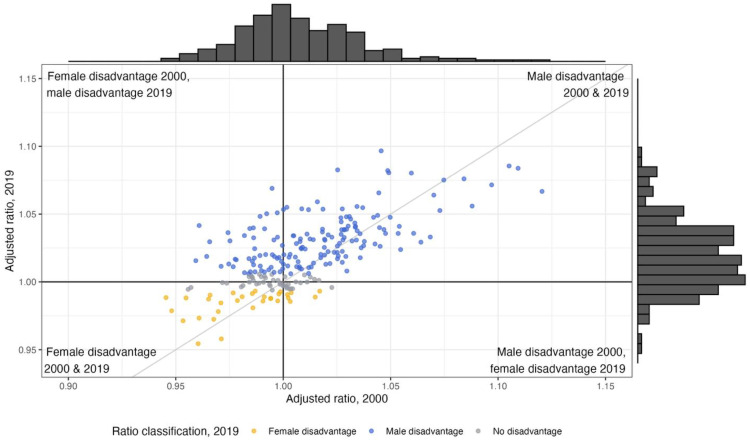
Comparison of adjusted ratios of life expectancy at birth in 2000 and 2019. Two countries were excluded from this plot for readability purposes: the Central African Republic (2000 ratio 0.98, 2019 ratio 1.39), and Russia (2000 ratio 1.15, 2019 ratio 1.10).

A comparison of the adjusted ratios of life expectancy at birth in all countries in 2000 and 2019 is shown in Fig 11. Between 2000 and 2019, the distribution of adjusted ratios coalesced around 1, and the number of countries with strong male disadvantage were reduced. In 2019, there were fewer countries with female disadvantage than in 2000. The lowest and highest adjusted ratios in 2000 were 0.95 in Mali and 1.15 in Russia; in 2019, the lowest and highest were 0.94 in Togo and 1.40 in the Central African Republic.

### Sensitivity analyses

The results from the sensitivity analyses are shown in S1 Appendix. The relationship between higher frontier percentiles (i.e., using a higher threshold for optimal life expectancy for each sex) and the frontier ratio is nonlinear, and all percentiles produced very similar ratios for each age until the 1% percentile for life expectancy at ages 50 and 70, which favored higher ratios and longer frontier life expectancy for females relative to males. Classification of inequality across buffer definitions was consistent for life expectancy at birth, where the buffer captures between 18% and 35% of the data. At older ages, definition of the buffer had a more significant effect on sex inequality classification. The life expectancy-based buffers showed increasing uncertainty with age, which is desirable since life expectancy is smaller at older ages. Absolute boundaries on the adjusted ratio showed the opposite pattern, implying increasing confidence in sex inequality classification with decreasing life expectancy.

## Discussion

We developed a simple approach to analyze and interpret sex inequalities in health outcomes. Our findings show females in 31 countries (13%) at age 0–78 countries (33%) at age 70 countries are more disadvantaged than males, despite having higher overall life expectancies. More than half of the countries remain male-disadvantaged, indicating that males are generally disadvantaged in most countries, even after adjustments. The number of countries with female disadvantage rises with age, especially in sub-Saharan Africa and Western Pacific and Southeastern Asia. We observed changes in disadvantage over time, with more countries classified as male disadvantaged in 2019 than in 2000. Our study shows variations across age, countries, and over time, suggesting that there is no general direction of sex disadvantage, and challenges common tendencies to state general disadvantage of one sex or another.

Most countries experience male disadvantage in life expectancy throughout the life span. Seventy-three countries (31%) had male disadvantage at all ages, and an additional 42 countries (18%) had male disadvantage at every age but one. Central Europe and Latin America, and the Caribbean were regions with disproportionate male disadvantage. Several different causes of higher male mortality have been proposed in the literature—mostly associated with social and structural factors, including lower education, homicides, wars, higher tobacco consumption, but also higher male infant mortality (which may be associated with biological susceptibility) [18–26]. Conflict-affected countries, such as the Central African Republic, Yemen, Syrian Arab Republic, and Libya, showed more male disadvantages [27–31]. Belarus, China, and Russia are in the top 10 countries globally for deaths due to tobacco use in adult males [32]. Of the countries with significant male disadvantage, Russia, Viet Nam, and Thailand had the highest sex differences in life expectancy at birth, at 10.4 years, 9.6 years, and 8.9 years, respectively. Thailand is recognized for achieving “good health with low cost,” and recent literature discussed Thailand’s female empowerment and gender equity as potential reasons for good health, but did not report the much lower life expectancy in males [33]. Notably, high life expectancy countries, such as Japan, Republic of Korea, and Taiwan, were also classified as male disadvantage. The fact that Japan was the frontier country for both sexes at multiple ages and still has sex disadvantage shows that inequalities persist even in countries with high longevity.

Although the global trend across countries points to male disadvantage, many countries still experience substantial female disadvantage over the life span. Even before adjustments, Nigeria and Togo experience lower life expectancy for females at age 5, and this gap is under-documented in the literature. Previous studies have found higher male childhood mortality in Nigeria between 2003 and 2013 [34,35]. The United Nations Inter-agency Group for Child Mortality Estimation did not report Nigeria and Togo as outliers with excess female child mortality [36]. India also showed female disadvantage across ages. Several studies have shown higher all-cause mortality and large female excess mortality in India’s younger population [23,36,37]. However, studies have also reported that mortality improvements have been greater among Indian females in recent years, and called for greater attention to preventing premature deaths in young males [38–40]. High-income northern European countries, such as the Netherlands, Iceland, and Sweden, also showed consistent female disadvantage. Studies show that gender equality in other aspects of life is correlated with narrower sex inequalities in life expectancy, attributed to female uptake of traditionally masculine behaviors and risks, such as smoking and alcohol consumption [41]. The female disadvantage observed in many countries at older ages is likely due to frontier females performing relatively better than frontier males, resulting in larger gaps between country-specific and frontier female life expectancy for females than for males.

There are pronounced trends in the direction of disadvantage across age and over time. Male disadvantage is present in the majority of countries at young ages, but by age 70, the proportion of countries with male and female disadvantage is about even. The distribution of life expectancy has greater dispersion for both sexes with age, but the dispersion is wider for females, which indicates that at old ages, countries are on average further from achieving female frontier life expectancy than males. Between 2000 and 2019, the number of countries with female disadvantage at birth decreased globally, and male disadvantage increased. This change is multifaceted. Frontier life expectancy increases were greater for males (5.3 years compared to 3.6 years for females), but average life expectancy increases for both sexes were more similar (5.2 years for males, 5.3 years for females). Additionally, some countries saw substantial improvements in female life expectancy that are consistent with other literature and were not matched by male improvements: 34 countries with female disadvantage in 2000 had male disadvantage in 2019, 20 of which were located in sub-Saharan Africa. The shift from female to male disadvantage at birth over time reflects improvements in female longevity in many countries (e.g., Afghanistan, Ethiopia, Tanzania), strong improvements in the male life expectancy in frontier countries (e.g., China, Hong Kong), and stagnation and even decline in male life expectancy in other parts of the world (e.g., Syria, Yemen, Libya).

Existing literature on measuring sex differences in health outcomes can be categorized into three sets. The first set directly compares outcomes between the two sexes without adjustments, i.e., estimates sex differences but not sex inequalities. For example, Baum and colleagues calculated the “gendered life expectancy difference” by subtracting male from female life expectancy at birth [42]. Patwardhan and colleagues calculated the absolute and relative differences in disability-adjusted life year rates between the sexes for 20 leading causes of disease burden [43]. They found substantial and persistent sex differences, with little progress in narrowing the gap since 1990. One limitation of such studies is that they cannot account for the possibility of a biological difference between sexes driving the presence of a gap. A key strength of our study is that it anchors outcomes to frontier countries, thus setting a more realistic and empirical sex gap to compare against. The second set, led by the United Nations Interagency Group for Child Mortality Estimation, compiles comprehensive data and uses Bayesian hierarchical time series models to estimate expected mortality rates and excess female mortality rates [36,37]. Similar to our study, their objective is to identify outliers with atypical sex ratios, i.e., sex inequalities. They found female disadvantage in 1–13 countries, depending on age group. They also found male disadvantage in 1–8 countries. Their outcome of interest is age-specific mortality rates, which are different but related to life expectancy at different ages. Applying our approach to age-specific mortality rates yields similar conclusions on sex inequalities. Our analytical approach is considerably simpler, but the objectives are aligned with those of the second set of studies. The third set compares the rates of decline in mortality rates by sex, for example, by the Lancet CIH [17,40]. Their findings are generally consistent with those of this paper, that in most countries, females have had higher rates of decline than males in the last decades.

This study has the following limitations. First, the frontier selection and buffer range are empirical choices, and other definitions could be equally valid. We explore the effects of alternative specifications in our sensitivity analyses. While our sex-specific benchmarks are based on the best-performing countries for each sex, they are not intended to represent purely biological differences. We recognize that observed sex gaps in life expectancy may be influenced by unmeasured factors such as genetic variation, environmental exposures, and their interactions with sex. Our choice of including different set of countries for the sex-specific frontier may also reflect broader differences in country characteristics beyond the sex differences. Alternatively, if data reports within-country life expectancy distribution (which WPP does not), one could define the frontier using the top decile or quintile of populations instead of the mean life expectancy (which we do here). Second, because the main objective of this paper is to introduce the methodology, we purposely presented just one outcome, life expectancy, not other important health outcomes. Life expectancy is a summary measure of the age-specific mortality rates of all consecutive ages (for example, life expectancy at birth is a summary of age-specific mortality rates up to the age 100+; life expectancy at age 35 summarizes mortality rates from 35 to 100+) [44]. The direction of sex disadvantage, therefore, may differ when applying to age-specific mortality or other outcomes. Most studies on sex differences do not discuss the implications of the choice of the measure, even though the conclusions on sex disadvantage may differ based on the measure. Furthermore, analyzing morbidity outcomes will likely lead to different conclusions about sex inequalities [43,45]. We also did not study sex inequalities beyond health. There is strong evidence on female disadvantage in sectors outside of health [46,47]. Third, Vaupel and colleagues emphasized wide life span variation even within each sex [48]. Estimation at the country level obfuscates subnational variation where sex inequalities may be more pronounced in the most disadvantaged populations. Fourth, we rely on WPP2024, which reflects data uncertainty and assumptions made by the statisticians, including “male mortality expected to exceed female mortality at older ages.” It is hard to determine how much of the inequalities quantified in this article is due to actual inequalities versus data uncertainty. WPP2024 also does not provide uncertainty intervals in their estimates. It is likely that sex differences in countries with better vital registration systems or larger population sizes are more precise. WPP2024 is, however, still the most reliable global data source on mortality. Fifth, the WPP2024 data and our approach does not allow for analysis of life expectancy by sex categories other than male/female (thus meaning that intersex people may not be included in the analysis) or of gender identity (which may be different to sex for people who identify as transgender or other gender categories). Lastly, while our study cannot provide insights into why one sex is more disadvantaged than the other in a country, the results point to the outliers that are more objectively categorized as disadvantaging one sex over another.

Sex and gender play a vital role in determining health outcomes, and sex inequalities exist in many countries [6]. Our findings show that females in 13%–33% of countries are more disadvantaged than males in terms of life expectancy at different ages, despite having higher life expectancies. In comparison, males in 33%–68% of the countries are more disadvantaged than females. The number of countries with female disadvantage rise with age, especially in sub-Saharan Africa and Western Pacific and Southeastern Asia. We call for better and more objective measurement frameworks applied to multiple health measures. The resulting evidence will serve to better guide gender-responsive approaches in global health and targeted policies and programmes that seek to focus on where need is greatest [6,49].

## Supporting information

S1 Appendix Appendix Table A. Adjusted ratio buffer boundaries, by age, 2019. Appendix Table B. Number of countries and proportion of world population classified as female disadvantage, male disadvantage, or no disadvantage before and after adjustment, by life expectancy at different ages, 2019. Appendix Table C. Adjusted sex ratios, unadjusted sex ratios, and life expectancy by sex at each age by country, 2019. Appendix Table D. Alternative frontier classification,  >5 million population countries. Appendix Table E. Number of countries with equal sex ratio or female/male disadvantage in life expectancy at different ages, removing countries not listed in the United Nations regions. Appendix Table F. Buffer definitions and life expectancies. Appendix Figure A. Distribution of differences and adjusted differences in 2019, by age. Appendix Figure B. Adjusted differences by life expectancy for the 30 most populous countries, 2019, by age. Appendix Figure C. Adjusted differences by CIH regions, 2019, by age. Appendix Figure D. Comparison of adjusted ratios and adjusted differences, by age. Appendix Figure E. Frontier ratio parameter sensitivity. Appendix Figure F. Country classification with various buffer methods, age 0 and 70.
(DOCX)

## Abbreviations

CIH: Commission on Investing in Health
WPP: World Population Prospects
