# Supplementary material for: From sex differences to sex inequalities in life expectancy: A cross-country observational benchmarking analysis
Source: PLoS Med. 2025 Dec 11;22(12):e1004828. doi: 10.1371/journal.pmed.1004828 (PMC12697978; doi:10.1371/journal.pmed.1004828)

**From sex differences to sex inequalities in life expectancy: A cross-country observational benchmarking analysis**

S1 APPENDIX

Table of Contents

[Appendix Table A. Adjusted ratio buffer boundaries, by age, 2019. 2](#_Toc205444540)

[Appendix Table B. Number of countries and proportion of world population classified as female disadvantage, male disadvantage, or no disadvantage before and after adjustment, by life expectancy at different ages, 2019. 3](#_Toc205444541)

[Appendix Table C. Adjusted sex ratios, unadjusted sex ratios, and life expectancy by sex at each age by country, 2019. 4](#_Toc205444542)

[Appendix Table D. Alternative frontier classification, >5 million population countries 40](#_Toc205444543)

[Appendix Table E. Number of countries with equal sex ratio or female/male disadvantage in life expectancy at different ages, removing countries not listed in the United Nations regions 41](#_Toc205444544)

[Appendix Table F. Buffer definitions and life expectancies 42](#_Toc205444545)

[Appendix Figure A. Distribution of differences and adjusted differences in 2019, by age 43](#_Toc205444546)

[Appendix Figure B. Adjusted differences by life expectancy for the 30 most populous countries, 2019, by age 44](#_Toc205444547)

[Appendix Figure C. Adjusted differences by CIH regions, 2019, by age 45](#_Toc205444548)

[Appendix Figure D. Comparison of adjusted ratios and adjusted differences, by age 50](#_Toc205444549)

[Appendix Figure E. Frontier ratio parameter sensitivity 51](#_Toc205444550)

[Appendix Figure F. Country classification with various buffer methods, age 0 and 70 52](#_Toc205444551)

## **Appendix Table A.** Adjusted ratio buffer boundaries, by age, 2019.

| Age | Buffer lower boundary | Buffer upper boundary |
| --- | --- | --- |
| 0 | 0.994 | 1.006 |
| 5 | 0.994 | 1.006 |
| 15 | 0.993 | 1.007 |
| 35 | 0.990 | 1.010 |
| 50 | 0.985 | 1.014 |
| 70 | 0.969 | 1.027 |

## **Appendix Table B.** Number of countries and proportion of world population classified as female disadvantage, male disadvantage, or no disadvantage before and after adjustment, by life expectancy at different ages, 2019.

|  | Original sex ratio | | | Adjusted sex ratio | | |
| --- | --- | --- | --- | --- | --- | --- |
|  | (A) Female disadvantage | (B) Male disadvantage | (C) No disadvantage | (A) Female disadvantage | (B) Male disadvantage | (C) No disadvantage |
| Birth | 0% (0 countries) | 100% (237 countries) | 0% (0 countries) | 24% (31 countries) | 67.56% (162 countries) | 8.44% (44 countries) |
| Age 5 | 2.79% (2 countries) | 97.21% (235 countries) | 0% (0 countries) | 28.86% (48 countries) | 59.07% (141 countries) | 12.07% (48 countries) |
| Age 15 | 0% (0 countries) | 100% (237 countries) | 0% (0 countries) | 27.86% (44 countries) | 59.32% (144 countries) | 12.82% (49 countries) |
| Age 35 | 0% (0 countries) | 100% (237 countries) | 0% (0 countries) | 11.37% (45 countries) | 50.86% (135 countries) | 37.77% (57 countries) |
| Age 50 | 0% (0 countries) | 100% (237 countries) | 0% (0 countries) | 33.86% (61 countries) | 47.49% (125 countries) | 18.65% (51 countries) |
| Age 70 | 0% (0 countries) | 100% (237 countries) | 0% (0 countries) | 48.17% (78 countries) | 31.61% (79 countries) | 20.22% (80 countries) |

## **Appendix Table C.** Adjusted sex ratios, unadjusted sex ratios, and life expectancy by sex at each age by country, 2019.

Life expectancy is from the UN World Population Prospect

Region acronyms in the table below are as follows: CA (Central Asia), CEE (Central and Eastern Europe), CIU (China, India and the USA), LAC (Latin America and the Caribbean), MENA (Middle East and North Africa), NA (North Atlantic), SSA (Sub-Saharan Africa), WPSA (Western Pacific and Southeast Asia)

* “None” means that we did not find any disadvantage for either sex

| Age | Country | Region | Male LE | Female LE | Raw ratio | Adjusted ratio | Disadvantage* |
| --- | --- | --- | --- | --- | --- | --- | --- |
| 0 | American Samoa |  | 70.1 | 75.72 | 1.08 | 1.03 | Male |
| 0 | Andorra |  | 82.18 | 86.1 | 1.05 | 0.99 | None |
| 0 | Anguilla |  | 75.08 | 82.15 | 1.09 | 1.04 | Male |
| 0 | Antigua And Barbuda |  | 74.14 | 79.84 | 1.08 | 1.02 | Male |
| 0 | Aruba |  | 73.05 | 78.81 | 1.08 | 1.02 | Male |
| 0 | Barbados |  | 74.29 | 78.54 | 1.06 | 1 | None |
| 0 | Bermuda |  | 77.61 | 85.03 | 1.1 | 1.04 | Male |
| 0 | Bonaire, Saint Eustatius And Saba |  | 74.71 | 80.44 | 1.08 | 1.02 | Male |
| 0 | British Virgin Islands |  | 74.14 | 79.65 | 1.07 | 1.02 | Male |
| 0 | Cayman Islands |  | 76.68 | 81.57 | 1.06 | 1.01 | Male |
| 0 | China, Hong Kong Sar |  | 82.28 | 88.24 | 1.07 | 1.02 | Male |
| 0 | China, Macao Sar |  | 80.58 | 85.98 | 1.07 | 1.01 | Male |
| 0 | Cook Islands |  | 71.11 | 78.43 | 1.1 | 1.05 | Male |
| 0 | Curaçao |  | 72.24 | 80.57 | 1.12 | 1.06 | Male |
| 0 | Dominica |  | 68.56 | 74.51 | 1.09 | 1.03 | Male |
| 0 | Falkland Islands (Malvinas) |  | 76.64 | 80.64 | 1.05 | 1 | None |
| 0 | Faroe Islands |  | 77.92 | 82.17 | 1.05 | 1 | None |
| 0 | French Guiana |  | 73.54 | 79.85 | 1.09 | 1.03 | Male |
| 0 | French Polynesia |  | 80.86 | 85.76 | 1.06 | 1.01 | Male |
| 0 | Gibraltar |  | 80.53 | 85.79 | 1.07 | 1.01 | Male |
| 0 | Greenland |  | 68.31 | 72.62 | 1.06 | 1.01 | Male |
| 0 | Grenada |  | 72.1 | 78.19 | 1.08 | 1.03 | Male |
| 0 | Guadeloupe |  | 77.4 | 85.06 | 1.1 | 1.04 | Male |
| 0 | Guam |  | 72.88 | 80.89 | 1.11 | 1.05 | Male |
| 0 | Guernsey |  | 81.12 | 85.41 | 1.05 | 1 | None |
| 0 | Holy See |  | 81.47 | 85.6 | 1.05 | 1 | None |
| 0 | Isle Of Man |  | 78.74 | 82.78 | 1.05 | 1 | None |
| 0 | Jersey |  | 77.66 | 82.24 | 1.06 | 1.01 | None |
| 0 | Kiribati |  | 64.03 | 67.5 | 1.05 | 1 | None |
| 0 | Kosovo (Under Unsc Res. 1244) |  | 75.06 | 79.36 | 1.06 | 1 | None |
| 0 | Liechtenstein |  | 81.47 | 85.22 | 1.05 | 0.99 | Female |
| 0 | Marshall Islands |  | 64.1 | 68.45 | 1.07 | 1.01 | Male |
| 0 | Martinique |  | 78.86 | 85.07 | 1.08 | 1.02 | Male |
| 0 | Mayotte |  | 74.31 | 78.11 | 1.05 | 1 | None |
| 0 | Micronesia (Fed. States Of) |  | 63.01 | 69.55 | 1.1 | 1.05 | Male |
| 0 | Monaco |  | 84.21 | 88.32 | 1.05 | 1 | None |
| 0 | Montserrat |  | 73.58 | 78.18 | 1.06 | 1.01 | Male |
| 0 | Nauru |  | 59.04 | 64.05 | 1.08 | 1.03 | Male |
| 0 | New Caledonia |  | 74.15 | 80.5 | 1.09 | 1.03 | Male |
| 0 | Niue |  | 66.62 | 72.13 | 1.08 | 1.03 | Male |
| 0 | Northern Mariana Islands |  | 76.17 | 79.82 | 1.05 | 0.99 | None |
| 0 | Palau |  | 66.46 | 72.04 | 1.08 | 1.03 | Male |
| 0 | Puerto Rico |  | 77.66 | 85.14 | 1.1 | 1.04 | Male |
| 0 | Réunion |  | 78.99 | 85.41 | 1.08 | 1.03 | Male |
| 0 | Saint Barthélemy |  | 79.95 | 87.57 | 1.1 | 1.04 | Male |
| 0 | Saint Helena |  | 73.9 | 80.48 | 1.09 | 1.03 | Male |
| 0 | Saint Kitts And Nevis |  | 68.02 | 75.48 | 1.11 | 1.05 | Male |
| 0 | Saint Lucia |  | 68.74 | 76.09 | 1.11 | 1.05 | Male |
| 0 | Saint Martin (French Part) |  | 76.46 | 83.84 | 1.1 | 1.04 | Male |
| 0 | Saint Pierre And Miquelon |  | 72.59 | 81.34 | 1.12 | 1.06 | Male |
| 0 | Saint Vincent And The Grenadines |  | 68.55 | 73.99 | 1.08 | 1.02 | Male |
| 0 | Samoa |  | 68.47 | 72.2 | 1.05 | 1 | None |
| 0 | San Marino |  | 83.59 | 86.79 | 1.04 | 0.99 | Female |
| 0 | Sao Tome And Principe |  | 64.78 | 71.86 | 1.11 | 1.05 | Male |
| 0 | Seychelles |  | 69.12 | 75.77 | 1.1 | 1.04 | Male |
| 0 | Sint Maarten (Dutch Part) |  | 73.13 | 78.58 | 1.07 | 1.02 | Male |
| 0 | State Of Palestine |  | 73.61 | 78.09 | 1.06 | 1.01 | Male |
| 0 | Tokelau |  | 74.42 | 78.34 | 1.05 | 1 | None |
| 0 | Tonga |  | 68.85 | 75.97 | 1.1 | 1.05 | Male |
| 0 | Turks And Caicos Islands |  | 75.56 | 79.97 | 1.06 | 1 | None |
| 0 | Tuvalu |  | 62.87 | 69.73 | 1.11 | 1.05 | Male |
| 0 | United States Virgin Islands |  | 70.01 | 80.86 | 1.15 | 1.1 | Male |
| 0 | Wallis And Futuna Islands |  | 77.06 | 78.99 | 1.03 | 0.97 | Female |
| 0 | Western Sahara |  | 68.8 | 72.34 | 1.05 | 1 | None |
| 0 | Albania | CEE | 77.83 | 81.06 | 1.04 | 0.99 | Female |
| 0 | Armenia | CEE | 71.02 | 78.83 | 1.11 | 1.05 | Male |
| 0 | Belarus | CEE | 69.22 | 79.01 | 1.14 | 1.08 | Male |
| 0 | Bosnia And Herzegovina | CEE | 73.96 | 80.46 | 1.09 | 1.03 | Male |
| 0 | Bulgaria | CEE | 71.58 | 78.73 | 1.1 | 1.04 | Male |
| 0 | Croatia | CEE | 74.91 | 81.38 | 1.09 | 1.03 | Male |
| 0 | Czechia | CEE | 76.27 | 82.07 | 1.08 | 1.02 | Male |
| 0 | Estonia | CEE | 74.38 | 82.72 | 1.11 | 1.06 | Male |
| 0 | Georgia | CEE | 69.22 | 78.89 | 1.14 | 1.08 | Male |
| 0 | Hungary | CEE | 73.05 | 79.69 | 1.09 | 1.04 | Male |
| 0 | Latvia | CEE | 70.88 | 79.99 | 1.13 | 1.07 | Male |
| 0 | Lithuania | CEE | 71.5 | 81.02 | 1.13 | 1.08 | Male |
| 0 | Montenegro | CEE | 73.24 | 79.98 | 1.09 | 1.04 | Male |
| 0 | North Macedonia | CEE | 74.51 | 79.06 | 1.06 | 1.01 | Male |
| 0 | Poland | CEE | 74.08 | 81.74 | 1.1 | 1.05 | Male |
| 0 | Republic Of Moldova | CEE | 65.57 | 74.62 | 1.14 | 1.08 | Male |
| 0 | Romania | CEE | 71.71 | 79.03 | 1.1 | 1.05 | Male |
| 0 | Russian Federation | CEE | 67.74 | 78.17 | 1.15 | 1.1 | Male |
| 0 | Serbia | CEE | 72.82 | 79.36 | 1.09 | 1.03 | Male |
| 0 | Slovakia | CEE | 74.26 | 81.01 | 1.09 | 1.04 | Male |
| 0 | Slovenia | CEE | 78.18 | 83.81 | 1.07 | 1.02 | Male |
| 0 | Ukraine | CEE | 68.87 | 78.73 | 1.14 | 1.09 | Male |
| 0 | Afghanistan | CA | 59.91 | 66.14 | 1.1 | 1.05 | Male |
| 0 | Azerbaijan | CA | 70.22 | 76.23 | 1.09 | 1.03 | Male |
| 0 | Kazakhstan | CA | 69.2 | 77.74 | 1.12 | 1.07 | Male |
| 0 | Kyrgyzstan | CA | 67.4 | 74.72 | 1.11 | 1.05 | Male |
| 0 | Mongolia | CA | 66.18 | 75.46 | 1.14 | 1.08 | Male |
| 0 | Pakistan | CA | 64.49 | 69.2 | 1.07 | 1.02 | Male |
| 0 | Tajikistan | CA | 68.73 | 73.18 | 1.06 | 1.01 | Male |
| 0 | Turkmenistan | CA | 66.1 | 72.31 | 1.09 | 1.04 | Male |
| 0 | Uzbekistan | CA | 68.92 | 75.37 | 1.09 | 1.04 | Male |
| 0 | China | CIU | 75.3 | 80.75 | 1.07 | 1.02 | Male |
| 0 | India | CIU | 69.31 | 72.29 | 1.04 | 0.99 | Female |
| 0 | Argentina | LAC | 74.16 | 79.44 | 1.07 | 1.02 | Male |
| 0 | Bahamas | LAC | 68.62 | 74.18 | 1.08 | 1.03 | Male |
| 0 | Belize | LAC | 69.87 | 75.67 | 1.08 | 1.03 | Male |
| 0 | Bolivia (Plurinational State Of) | LAC | 65.54 | 70.27 | 1.07 | 1.02 | Male |
| 0 | Brazil | LAC | 72.65 | 79.02 | 1.09 | 1.03 | Male |
| 0 | Chile | LAC | 78.1 | 82.55 | 1.06 | 1 | None |
| 0 | Colombia | LAC | 73.91 | 79.7 | 1.08 | 1.02 | Male |
| 0 | Costa Rica | LAC | 77.6 | 82.96 | 1.07 | 1.02 | Male |
| 0 | Cuba | LAC | 75 | 79.91 | 1.07 | 1.01 | Male |
| 0 | Dominican Republic | LAC | 69.9 | 76.45 | 1.09 | 1.04 | Male |
| 0 | Ecuador | LAC | 74.61 | 79.98 | 1.07 | 1.02 | Male |
| 0 | El Salvador | LAC | 67.1 | 75.98 | 1.13 | 1.08 | Male |
| 0 | Guatemala | LAC | 69.28 | 74.02 | 1.07 | 1.01 | Male |
| 0 | Guyana | LAC | 65.53 | 72.85 | 1.11 | 1.06 | Male |
| 0 | Haiti | LAC | 61.48 | 67.29 | 1.09 | 1.04 | Male |
| 0 | Honduras | LAC | 69.65 | 74.73 | 1.07 | 1.02 | Male |
| 0 | Jamaica | LAC | 69.08 | 74.01 | 1.07 | 1.02 | Male |
| 0 | Mexico | LAC | 71.59 | 77.42 | 1.08 | 1.03 | Male |
| 0 | Nicaragua | LAC | 71.02 | 76.42 | 1.08 | 1.02 | Male |
| 0 | Panama | LAC | 75.53 | 81.57 | 1.08 | 1.03 | Male |
| 0 | Paraguay | LAC | 70.73 | 76.8 | 1.09 | 1.03 | Male |
| 0 | Peru | LAC | 74.11 | 78.58 | 1.06 | 1.01 | Male |
| 0 | Suriname | LAC | 68.64 | 75.08 | 1.09 | 1.04 | Male |
| 0 | Trinidad And Tobago | LAC | 69.73 | 76.03 | 1.09 | 1.04 | Male |
| 0 | Uruguay | LAC | 73.58 | 81.29 | 1.1 | 1.05 | Male |
| 0 | Venezuela (Bolivarian Republic Of) | LAC | 69.02 | 76.68 | 1.11 | 1.05 | Male |
| 0 | Algeria | MENA | 74.35 | 77.09 | 1.04 | 0.98 | Female |
| 0 | Bahrain | MENA | 79.61 | 81.54 | 1.02 | 0.97 | Female |
| 0 | Egypt | MENA | 69.04 | 73.42 | 1.06 | 1.01 | Male |
| 0 | Iran (Islamic Republic Of) | MENA | 74.99 | 78.83 | 1.05 | 1 | None |
| 0 | Iraq | MENA | 69.33 | 73.01 | 1.05 | 1 | None |
| 0 | Israel | MENA | 80.99 | 84.72 | 1.05 | 0.99 | Female |
| 0 | Jordan | MENA | 74.81 | 79.23 | 1.06 | 1.01 | None |
| 0 | Kuwait | MENA | 78.98 | 81.59 | 1.03 | 0.98 | Female |
| 0 | Lebanon | MENA | 76.21 | 80.05 | 1.05 | 1 | None |
| 0 | Libya | MENA | 70.08 | 76.2 | 1.09 | 1.03 | Male |
| 0 | Morocco | MENA | 72.25 | 76.39 | 1.06 | 1 | None |
| 0 | Oman | MENA | 78.43 | 81.87 | 1.04 | 0.99 | Female |
| 0 | Qatar | MENA | 82.18 | 84.06 | 1.02 | 0.97 | Female |
| 0 | Saudi Arabia | MENA | 76.94 | 80.38 | 1.04 | 0.99 | Female |
| 0 | Syrian Arab Republic | MENA | 67.83 | 74.25 | 1.09 | 1.04 | Male |
| 0 | Tunisia | MENA | 73.04 | 78.26 | 1.07 | 1.02 | Male |
| 0 | Türkiye | MENA | 74.68 | 80.89 | 1.08 | 1.03 | Male |
| 0 | United Arab Emirates | MENA | 81.59 | 84.05 | 1.03 | 0.98 | Female |
| 0 | Yemen | MENA | 63.34 | 70.06 | 1.11 | 1.05 | Male |
| 0 | Austria | NA | 79.53 | 84.19 | 1.06 | 1.01 | None |
| 0 | Belgium | NA | 79.6 | 84 | 1.06 | 1 | None |
| 0 | Canada | NA | 80.17 | 84.36 | 1.05 | 1 | None |
| 0 | Cyprus | NA | 79.32 | 83.63 | 1.05 | 1 | None |
| 0 | Denmark | NA | 79.44 | 83.42 | 1.05 | 1 | None |
| 0 | Finland | NA | 79.17 | 84.51 | 1.07 | 1.01 | Male |
| 0 | France | NA | 79.75 | 85.57 | 1.07 | 1.02 | Male |
| 0 | Germany | NA | 78.83 | 83.54 | 1.06 | 1.01 | Male |
| 0 | Greece | NA | 78.74 | 83.87 | 1.07 | 1.01 | Male |
| 0 | Iceland | NA | 81.3 | 84.44 | 1.04 | 0.99 | Female |
| 0 | Ireland | NA | 80.41 | 84.4 | 1.05 | 1 | None |
| 0 | Italy | NA | 81.14 | 85.4 | 1.05 | 1 | None |
| 0 | Luxembourg | NA | 79.91 | 83.17 | 1.04 | 0.99 | Female |
| 0 | Malta | NA | 80.83 | 84.73 | 1.05 | 1 | None |
| 0 | Netherlands | NA | 80.47 | 83.56 | 1.04 | 0.99 | Female |
| 0 | Norway | NA | 81.18 | 84.69 | 1.04 | 0.99 | Female |
| 0 | Portugal | NA | 78.97 | 84.75 | 1.07 | 1.02 | Male |
| 0 | Spain | NA | 80.74 | 86.14 | 1.07 | 1.01 | Male |
| 0 | Sweden | NA | 81.35 | 84.73 | 1.04 | 0.99 | Female |
| 0 | Switzerland | NA | 81.9 | 85.56 | 1.04 | 0.99 | Female |
| 0 | United Kingdom | NA | 79.55 | 83.28 | 1.05 | 0.99 | None |
| 0 | Angola | SSA | 60.61 | 65.51 | 1.08 | 1.03 | Male |
| 0 | Benin | SSA | 58.69 | 61.07 | 1.04 | 0.99 | Female |
| 0 | Botswana | SSA | 64.77 | 69.59 | 1.07 | 1.02 | Male |
| 0 | Burkina Faso | SSA | 58.33 | 61.95 | 1.06 | 1.01 | Male |
| 0 | Burundi | SSA | 60.31 | 64.03 | 1.06 | 1.01 | Male |
| 0 | Cabo Verde | SSA | 72.13 | 78.69 | 1.09 | 1.04 | Male |
| 0 | Cameroon | SSA | 59.63 | 63.83 | 1.07 | 1.02 | Male |
| 0 | Central African Republic | SSA | 26.39 | 38.77 | 1.47 | 1.4 | Male |
| 0 | Chad | SSA | 51.44 | 54.58 | 1.06 | 1.01 | Male |
| 0 | Comoros | SSA | 63.79 | 67.68 | 1.06 | 1.01 | Male |
| 0 | Congo | SSA | 61.71 | 64.63 | 1.05 | 0.99 | None |
| 0 | Côte D'ivoire | SSA | 58.49 | 62.29 | 1.06 | 1.01 | Male |
| 0 | Democratic Republic Of The Congo | SSA | 58.36 | 62.15 | 1.07 | 1.01 | Male |
| 0 | Djibouti | SSA | 61.97 | 66.86 | 1.08 | 1.02 | Male |
| 0 | Equatorial Guinea | SSA | 60.67 | 64.14 | 1.06 | 1 | None |
| 0 | Eritrea | SSA | 65.17 | 69.36 | 1.06 | 1.01 | Male |
| 0 | Eswatini | SSA | 57.11 | 62.54 | 1.1 | 1.04 | Male |
| 0 | Ethiopia | SSA | 62.78 | 68.86 | 1.1 | 1.04 | Male |
| 0 | Gabon | SSA | 65.15 | 69.76 | 1.07 | 1.02 | Male |
| 0 | Gambia | SSA | 62.91 | 65.93 | 1.05 | 0.99 | None |
| 0 | Ghana | SSA | 62.39 | 66.57 | 1.07 | 1.01 | Male |
| 0 | Guinea | SSA | 58.16 | 60.53 | 1.04 | 0.99 | Female |
| 0 | Guinea-Bissau | SSA | 59.87 | 64.31 | 1.07 | 1.02 | Male |
| 0 | Kenya | SSA | 60.86 | 65.1 | 1.07 | 1.02 | Male |
| 0 | Lesotho | SSA | 52.72 | 57.67 | 1.09 | 1.04 | Male |
| 0 | Liberia | SSA | 60.02 | 62.44 | 1.04 | 0.99 | Female |
| 0 | Madagascar | SSA | 61.91 | 65.14 | 1.05 | 1 | None |
| 0 | Malawi | SSA | 61.58 | 68.43 | 1.11 | 1.06 | Male |
| 0 | Mali | SSA | 58.04 | 60.42 | 1.04 | 0.99 | Female |
| 0 | Mauritania | SSA | 65.82 | 69.52 | 1.06 | 1 | None |
| 0 | Mauritius | SSA | 71.11 | 77.52 | 1.09 | 1.04 | Male |
| 0 | Mozambique | SSA | 58.52 | 63.74 | 1.09 | 1.03 | Male |
| 0 | Namibia | SSA | 59.78 | 67.3 | 1.13 | 1.07 | Male |
| 0 | Niger | SSA | 59.13 | 60.95 | 1.03 | 0.98 | Female |
| 0 | Nigeria | SSA | 52.77 | 53.24 | 1.01 | 0.96 | Female |
| 0 | Rwanda | SSA | 64.36 | 68.81 | 1.07 | 1.02 | Male |
| 0 | Senegal | SSA | 65.86 | 69.57 | 1.06 | 1 | None |
| 0 | Sierra Leone | SSA | 58.3 | 60.87 | 1.04 | 0.99 | Female |
| 0 | Somalia | SSA | 55.24 | 59.31 | 1.07 | 1.02 | Male |
| 0 | South Africa | SSA | 62.7 | 69.22 | 1.1 | 1.05 | Male |
| 0 | South Sudan | SSA | 55.73 | 60.47 | 1.09 | 1.03 | Male |
| 0 | Sudan | SSA | 63.2 | 68.64 | 1.09 | 1.03 | Male |
| 0 | Togo | SSA | 60.89 | 61.2 | 1.01 | 0.95 | Female |
| 0 | Uganda | SSA | 63.48 | 69.13 | 1.09 | 1.03 | Male |
| 0 | United Republic Of Tanzania | SSA | 63.14 | 68.92 | 1.09 | 1.04 | Male |
| 0 | Zambia | SSA | 60.35 | 65.44 | 1.08 | 1.03 | Male |
| 0 | Zimbabwe | SSA | 58.83 | 63.05 | 1.07 | 1.02 | Male |
| 0 | United States Of America | CIU | 76.45 | 81.47 | 1.07 | 1.01 | Male |
| 0 | Australia | WPSA | 81.21 | 85.28 | 1.05 | 1 | None |
| 0 | Bangladesh | WPSA | 70.93 | 74.4 | 1.05 | 1 | None |
| 0 | Bhutan | WPSA | 70.43 | 73.9 | 1.05 | 1 | None |
| 0 | Brunei Darussalam | WPSA | 73.07 | 77.29 | 1.06 | 1 | None |
| 0 | Cambodia | WPSA | 67.53 | 72.56 | 1.07 | 1.02 | Male |
| 0 | Dem. People's Republic Of Korea | WPSA | 69.94 | 75.86 | 1.08 | 1.03 | Male |
| 0 | Fiji | WPSA | 65.12 | 68.99 | 1.06 | 1.01 | Male |
| 0 | Indonesia | WPSA | 68.32 | 72.39 | 1.06 | 1.01 | Male |
| 0 | Japan | WPSA | 81.36 | 87.43 | 1.07 | 1.02 | Male |
| 0 | Lao People's Democratic Republic | WPSA | 65.78 | 70.16 | 1.07 | 1.01 | Male |
| 0 | Malaysia | WPSA | 73.54 | 78.6 | 1.07 | 1.01 | Male |
| 0 | Maldives | WPSA | 78.39 | 81.51 | 1.04 | 0.99 | Female |
| 0 | Myanmar | WPSA | 63.57 | 69.48 | 1.09 | 1.04 | Male |
| 0 | Nepal | WPSA | 67.87 | 70.7 | 1.04 | 0.99 | Female |
| 0 | New Zealand | WPSA | 80.01 | 83.65 | 1.05 | 0.99 | Female |
| 0 | Papua New Guinea | WPSA | 62.9 | 68.43 | 1.09 | 1.03 | Male |
| 0 | Philippines | WPSA | 66.66 | 72.7 | 1.09 | 1.04 | Male |
| 0 | Republic Of Korea | WPSA | 80.34 | 86.74 | 1.08 | 1.03 | Male |
| 0 | Singapore | WPSA | 81.46 | 85.79 | 1.05 | 1 | None |
| 0 | Solomon Islands | WPSA | 68.75 | 71.36 | 1.04 | 0.99 | Female |
| 0 | Sri Lanka | WPSA | 73.42 | 79.89 | 1.09 | 1.03 | Male |
| 0 | Taiwan | WPSA | 77.37 | 84.07 | 1.09 | 1.03 | Male |
| 0 | Thailand | WPSA | 72.85 | 81.76 | 1.12 | 1.07 | Male |
| 0 | Timor-Leste | WPSA | 64.85 | 68.28 | 1.05 | 1 | None |
| 0 | Vanuatu | WPSA | 68.85 | 73.29 | 1.06 | 1.01 | Male |
| 0 | Viet Nam | WPSA | 69.41 | 78.97 | 1.14 | 1.08 | Male |
| 5 | American Samoa |  | 65.91 | 71.51 | 1.08 | 1.03 | Male |
| 5 | Andorra |  | 77.68 | 81.63 | 1.05 | 0.99 | Female |
| 5 | Anguilla |  | 70.72 | 77.51 | 1.1 | 1.04 | Male |
| 5 | Antigua And Barbuda |  | 69.97 | 75.58 | 1.08 | 1.02 | Male |
| 5 | Aruba |  | 69.3 | 74.9 | 1.08 | 1.02 | Male |
| 5 | Barbados |  | 70.25 | 74.41 | 1.06 | 1 | None |
| 5 | Bermuda |  | 72.94 | 80.18 | 1.1 | 1.04 | Male |
| 5 | Bonaire, Sint Eustatius And Saba |  | 70.82 | 76.38 | 1.08 | 1.02 | Male |
| 5 | British Virgin Islands |  | 70.36 | 75.58 | 1.07 | 1.02 | Male |
| 5 | Cayman Islands |  | 71.87 | 76.73 | 1.07 | 1.01 | Male |
| 5 | China, Hong Kong Sar |  | 77.46 | 83.41 | 1.08 | 1.02 | Male |
| 5 | China, Macao Sar |  | 75.96 | 81.15 | 1.07 | 1.01 | Male |
| 5 | Cook Islands |  | 66.67 | 74.01 | 1.11 | 1.05 | Male |
| 5 | Curaçao |  | 68.15 | 76.27 | 1.12 | 1.06 | Male |
| 5 | Dominica |  | 65.81 | 71.67 | 1.09 | 1.03 | Male |
| 5 | Falkland Islands (Malvinas) |  | 72.52 | 76.55 | 1.06 | 1 | None |
| 5 | Faroe Islands |  | 73.65 | 77.71 | 1.06 | 1 | None |
| 5 | French Guiana |  | 69.63 | 75.53 | 1.08 | 1.03 | Male |
| 5 | French Polynesia |  | 76.42 | 81.31 | 1.06 | 1.01 | None |
| 5 | Gibraltar |  | 76.54 | 81.59 | 1.07 | 1.01 | Male |
| 5 | Greenland |  | 64.16 | 68.36 | 1.07 | 1.01 | Male |
| 5 | Grenada |  | 68.33 | 74.33 | 1.09 | 1.03 | Male |
| 5 | Guadeloupe |  | 73.23 | 80.66 | 1.1 | 1.04 | Male |
| 5 | Guam |  | 68.96 | 76.86 | 1.11 | 1.05 | Male |
| 5 | Guernsey |  | 76.67 | 80.98 | 1.06 | 1 | None |
| 5 | Holy See |  | 77 | 81.16 | 1.05 | 1 | None |
| 5 | Isle Of Man |  | 74.9 | 78.86 | 1.05 | 1 | None |
| 5 | Jersey |  | 73.44 | 78.03 | 1.06 | 1 | None |
| 5 | Kiribati |  | 63.32 | 66.26 | 1.05 | 0.99 | Female |
| 5 | Kosovo (Under Unsc Res. 1244) |  | 71 | 75.16 | 1.06 | 1 | None |
| 5 | Liechtenstein |  | 77.01 | 80.8 | 1.05 | 0.99 | Female |
| 5 | Marshall Islands |  | 61.42 | 65.39 | 1.06 | 1.01 | None |
| 5 | Martinique |  | 74.6 | 80.74 | 1.08 | 1.02 | Male |
| 5 | Mayotte |  | 70.19 | 73.9 | 1.05 | 0.99 | None |
| 5 | Micronesia (Fed. States Of) |  | 59.9 | 66.13 | 1.1 | 1.04 | Male |
| 5 | Monaco |  | 79.61 | 83.73 | 1.05 | 0.99 | None |
| 5 | Montserrat |  | 69.1 | 73.77 | 1.07 | 1.01 | Male |
| 5 | Nauru |  | 55.94 | 60.79 | 1.09 | 1.03 | Male |
| 5 | New Caledonia |  | 70.26 | 76.38 | 1.09 | 1.03 | Male |
| 5 | Niue |  | 63.67 | 68.92 | 1.08 | 1.02 | Male |
| 5 | Northern Mariana Islands |  | 71.79 | 75.39 | 1.05 | 0.99 | Female |
| 5 | Palau |  | 63.15 | 68.51 | 1.08 | 1.03 | Male |
| 5 | Puerto Rico |  | 73.23 | 80.7 | 1.1 | 1.04 | Male |
| 5 | Réunion |  | 74.53 | 80.94 | 1.09 | 1.03 | Male |
| 5 | Saint Barthélemy |  | 75.57 | 83.02 | 1.1 | 1.04 | Male |
| 5 | Saint Helena |  | 70.14 | 76.4 | 1.09 | 1.03 | Male |
| 5 | Saint Kitts And Nevis |  | 64.28 | 71.61 | 1.11 | 1.05 | Male |
| 5 | Saint Lucia |  | 65.07 | 72.3 | 1.11 | 1.05 | Male |
| 5 | Saint Martin (French Part) |  | 72.36 | 79.52 | 1.1 | 1.04 | Male |
| 5 | Saint Pierre And Miquelon |  | 68.51 | 76.97 | 1.12 | 1.06 | Male |
| 5 | Saint Vincent And The Grenadines |  | 64.5 | 70.15 | 1.09 | 1.03 | Male |
| 5 | Samoa |  | 64.98 | 68.53 | 1.05 | 1 | None |
| 5 | San Marino |  | 78.74 | 81.92 | 1.04 | 0.98 | Female |
| 5 | Sao Tome And Principe |  | 60.97 | 68.05 | 1.12 | 1.05 | Male |
| 5 | Seychelles |  | 65.22 | 71.81 | 1.1 | 1.04 | Male |
| 5 | Sint Maarten (Dutch Part) |  | 69.51 | 74.67 | 1.07 | 1.02 | Male |
| 5 | State Of Palestine |  | 69.89 | 74.24 | 1.06 | 1 | None |
| 5 | Tokelau |  | 69.99 | 73.73 | 1.05 | 1 | None |
| 5 | Tonga |  | 64.75 | 71.74 | 1.11 | 1.05 | Male |
| 5 | Turks And Caicos Islands |  | 71.09 | 75.41 | 1.06 | 1 | None |
| 5 | Tuvalu |  | 59.47 | 66.13 | 1.11 | 1.05 | Male |
| 5 | United States Virgin Islands |  | 65.53 | 76.34 | 1.16 | 1.1 | Male |
| 5 | Wallis And Futuna Islands |  | 72.81 | 75.43 | 1.04 | 0.98 | Female |
| 5 | Western Sahara |  | 66.34 | 69.47 | 1.05 | 0.99 | Female |
| 5 | Albania | CEE | 73.64 | 76.77 | 1.04 | 0.99 | Female |
| 5 | Armenia | CEE | 66.96 | 74.67 | 1.12 | 1.05 | Male |
| 5 | Belarus | CEE | 64.46 | 74.22 | 1.15 | 1.09 | Male |
| 5 | Bosnia And Herzegovina | CEE | 69.46 | 75.92 | 1.09 | 1.03 | Male |
| 5 | Bulgaria | CEE | 67.08 | 74.23 | 1.11 | 1.05 | Male |
| 5 | Croatia | CEE | 70.27 | 76.71 | 1.09 | 1.03 | Male |
| 5 | Czechia | CEE | 71.53 | 77.29 | 1.08 | 1.02 | Male |
| 5 | Estonia | CEE | 69.61 | 77.82 | 1.12 | 1.06 | Male |
| 5 | Georgia | CEE | 64.98 | 74.57 | 1.15 | 1.08 | Male |
| 5 | Hungary | CEE | 68.41 | 74.99 | 1.1 | 1.04 | Male |
| 5 | Latvia | CEE | 66.17 | 75.33 | 1.14 | 1.08 | Male |
| 5 | Lithuania | CEE | 66.8 | 76.27 | 1.14 | 1.08 | Male |
| 5 | Montenegro | CEE | 68.56 | 75.25 | 1.1 | 1.04 | Male |
| 5 | North Macedonia | CEE | 70.09 | 74.49 | 1.06 | 1 | None |
| 5 | Poland | CEE | 69.42 | 77.08 | 1.11 | 1.05 | Male |
| 5 | Republic Of Moldova | CEE | 61.66 | 70.6 | 1.15 | 1.08 | Male |
| 5 | Romania | CEE | 67.26 | 74.54 | 1.11 | 1.05 | Male |
| 5 | Russian Federation | CEE | 63.17 | 73.58 | 1.16 | 1.1 | Male |
| 5 | Serbia | CEE | 68.27 | 74.76 | 1.1 | 1.03 | Male |
| 5 | Slovakia | CEE | 69.76 | 76.46 | 1.1 | 1.04 | Male |
| 5 | Slovenia | CEE | 73.41 | 78.98 | 1.08 | 1.02 | Male |
| 5 | Ukraine | CEE | 64.44 | 74.29 | 1.15 | 1.09 | Male |
| 5 | Afghanistan | CA | 59.21 | 65.33 | 1.1 | 1.04 | Male |
| 5 | Azerbaijan | CA | 66.86 | 72.69 | 1.09 | 1.03 | Male |
| 5 | Kazakhstan | CA | 65 | 73.44 | 1.13 | 1.07 | Male |
| 5 | Kyrgyzstan | CA | 63.81 | 70.96 | 1.11 | 1.05 | Male |
| 5 | Mongolia | CA | 62.35 | 71.52 | 1.15 | 1.08 | Male |
| 5 | Pakistan | CA | 64.43 | 68.78 | 1.07 | 1.01 | Male |
| 5 | Tajikistan | CA | 66.41 | 70.38 | 1.06 | 1 | None |
| 5 | Turkmenistan | CA | 64.41 | 70 | 1.09 | 1.03 | Male |
| 5 | Uzbekistan | CA | 65.15 | 71.39 | 1.1 | 1.04 | Male |
| 5 | China | CIU | 70.95 | 76.36 | 1.08 | 1.02 | Male |
| 5 | India | CIU | 66.72 | 69.9 | 1.05 | 0.99 | Female |
| 5 | Argentina | LAC | 70.08 | 75.29 | 1.07 | 1.02 | Male |
| 5 | Bahamas | LAC | 65.36 | 70.94 | 1.09 | 1.03 | Male |
| 5 | Belize | LAC | 65.86 | 71.55 | 1.09 | 1.03 | Male |
| 5 | Bolivia (Plurinational State Of) | LAC | 63.87 | 68.45 | 1.07 | 1.01 | Male |
| 5 | Brazil | LAC | 68.86 | 75.07 | 1.09 | 1.03 | Male |
| 5 | Chile | LAC | 73.66 | 78.06 | 1.06 | 1 | None |
| 5 | Colombia | LAC | 70.03 | 75.68 | 1.08 | 1.02 | Male |
| 5 | Costa Rica | LAC | 73.29 | 78.6 | 1.07 | 1.01 | Male |
| 5 | Cuba | LAC | 70.56 | 75.33 | 1.07 | 1.01 | Male |
| 5 | Dominican Republic | LAC | 67.56 | 73.87 | 1.09 | 1.03 | Male |
| 5 | Ecuador | LAC | 70.73 | 75.93 | 1.07 | 1.01 | Male |
| 5 | El Salvador | LAC | 63.08 | 71.89 | 1.14 | 1.08 | Male |
| 5 | Guatemala | LAC | 66.2 | 70.66 | 1.07 | 1.01 | Male |
| 5 | Guyana | LAC | 62.73 | 69.74 | 1.11 | 1.05 | Male |
| 5 | Haiti | LAC | 60.88 | 66.27 | 1.09 | 1.03 | Male |
| 5 | Honduras | LAC | 66.04 | 70.92 | 1.07 | 1.01 | Male |
| 5 | Jamaica | LAC | 65.59 | 70.28 | 1.07 | 1.01 | Male |
| 5 | Mexico | LAC | 67.71 | 73.42 | 1.08 | 1.02 | Male |
| 5 | Nicaragua | LAC | 67.36 | 72.56 | 1.08 | 1.02 | Male |
| 5 | Panama | LAC | 72 | 77.89 | 1.08 | 1.02 | Male |
| 5 | Paraguay | LAC | 67.26 | 73.15 | 1.09 | 1.03 | Male |
| 5 | Peru | LAC | 70.41 | 74.71 | 1.06 | 1 | None |
| 5 | Suriname | LAC | 65.05 | 71.29 | 1.1 | 1.04 | Male |
| 5 | Trinidad And Tobago | LAC | 66.08 | 72.25 | 1.09 | 1.03 | Male |
| 5 | Uruguay | LAC | 69.21 | 76.89 | 1.11 | 1.05 | Male |
| 5 | Venezuela (Bolivarian Republic Of) | LAC | 65.47 | 72.97 | 1.11 | 1.05 | Male |
| 5 | Algeria | MENA | 71.24 | 73.79 | 1.04 | 0.98 | Female |
| 5 | Bahrain | MENA | 75.19 | 77.09 | 1.03 | 0.97 | Female |
| 5 | Egypt | MENA | 65.54 | 69.81 | 1.07 | 1.01 | Male |
| 5 | Iran (Islamic Republic Of) | MENA | 71.05 | 74.85 | 1.05 | 1 | None |
| 5 | Iraq | MENA | 66.36 | 69.77 | 1.05 | 0.99 | Female |
| 5 | Israel | MENA | 76.33 | 79.98 | 1.05 | 0.99 | Female |
| 5 | Jordan | MENA | 71.09 | 75.35 | 1.06 | 1 | None |
| 5 | Kuwait | MENA | 74.74 | 77.24 | 1.03 | 0.98 | Female |
| 5 | Lebanon | MENA | 72.43 | 76.17 | 1.05 | 0.99 | None |
| 5 | Libya | MENA | 66.04 | 72.08 | 1.09 | 1.03 | Male |
| 5 | Morocco | MENA | 68.81 | 72.74 | 1.06 | 1 | None |
| 5 | Oman | MENA | 74.36 | 77.67 | 1.04 | 0.99 | Female |
| 5 | Qatar | MENA | 77.72 | 79.55 | 1.02 | 0.97 | Female |
| 5 | Saudi Arabia | MENA | 72.52 | 75.95 | 1.05 | 0.99 | Female |
| 5 | Syrian Arab Republic | MENA | 64.69 | 70.96 | 1.1 | 1.04 | Male |
| 5 | Tunisia | MENA | 69.17 | 74.28 | 1.07 | 1.01 | Male |
| 5 | Türkiye | MENA | 70.56 | 76.73 | 1.09 | 1.03 | Male |
| 5 | United Arab Emirates | MENA | 77.2 | 79.56 | 1.03 | 0.97 | Female |
| 5 | Yemen | MENA | 61.61 | 68.15 | 1.11 | 1.05 | Male |
| 5 | Austria | NA | 74.86 | 79.47 | 1.06 | 1 | None |
| 5 | Belgium | NA | 74.96 | 79.31 | 1.06 | 1 | None |
| 5 | Canada | NA | 75.63 | 79.75 | 1.05 | 1 | None |
| 5 | Cyprus | NA | 74.59 | 78.88 | 1.06 | 1 | None |
| 5 | Denmark | NA | 74.74 | 78.66 | 1.05 | 0.99 | None |
| 5 | Finland | NA | 74.4 | 79.69 | 1.07 | 1.01 | Male |
| 5 | France | NA | 75.14 | 80.9 | 1.08 | 1.02 | Male |
| 5 | Germany | NA | 74.15 | 78.82 | 1.06 | 1 | None |
| 5 | Greece | NA | 74.09 | 79.18 | 1.07 | 1.01 | Male |
| 5 | Iceland | NA | 76.53 | 79.5 | 1.04 | 0.98 | Female |
| 5 | Ireland | NA | 75.72 | 79.68 | 1.05 | 0.99 | None |
| 5 | Italy | NA | 76.4 | 80.63 | 1.06 | 1 | None |
| 5 | Luxembourg | NA | 75.52 | 78.74 | 1.04 | 0.99 | Female |
| 5 | Malta | NA | 76.33 | 80.58 | 1.06 | 1 | None |
| 5 | Netherlands | NA | 75.83 | 78.88 | 1.04 | 0.98 | Female |
| 5 | Norway | NA | 76.42 | 79.87 | 1.05 | 0.99 | Female |
| 5 | Portugal | NA | 74.27 | 80.03 | 1.08 | 1.02 | Male |
| 5 | Spain | NA | 76.01 | 81.38 | 1.07 | 1.01 | Male |
| 5 | Sweden | NA | 76.56 | 79.93 | 1.04 | 0.99 | Female |
| 5 | Switzerland | NA | 77.22 | 80.87 | 1.05 | 0.99 | Female |
| 5 | United Kingdom | NA | 74.94 | 78.61 | 1.05 | 0.99 | Female |
| 5 | Angola | SSA | 60.85 | 65.24 | 1.07 | 1.01 | Male |
| 5 | Benin | SSA | 59.68 | 61.4 | 1.03 | 0.97 | Female |
| 5 | Botswana | SSA | 62.84 | 67.39 | 1.07 | 1.01 | Male |
| 5 | Burkina Faso | SSA | 59.2 | 62.45 | 1.05 | 1 | None |
| 5 | Burundi | SSA | 59.2 | 62.5 | 1.06 | 1 | None |
| 5 | Cabo Verde | SSA | 68.26 | 74.7 | 1.09 | 1.03 | Male |
| 5 | Cameroon | SSA | 59.97 | 63.65 | 1.06 | 1 | None |
| 5 | Central African Republic | SSA | 29.52 | 45.02 | 1.53 | 1.44 | Male |
| 5 | Chad | SSA | 53.32 | 55.94 | 1.05 | 0.99 | Female |
| 5 | Comoros | SSA | 62.55 | 66.24 | 1.06 | 1 | None |
| 5 | Congo | SSA | 60.15 | 62.84 | 1.04 | 0.99 | Female |
| 5 | Côte D'ivoire | SSA | 58.83 | 61.76 | 1.05 | 0.99 | Female |
| 5 | Democratic Republic Of The Congo | SSA | 59.08 | 62.26 | 1.05 | 1 | None |
| 5 | Djibouti | SSA | 61.04 | 65.5 | 1.07 | 1.01 | Male |
| 5 | Equatorial Guinea | SSA | 61.39 | 64.28 | 1.05 | 0.99 | Female |
| 5 | Eritrea | SSA | 63.24 | 66.88 | 1.06 | 1 | None |
| 5 | Eswatini | SSA | 55.74 | 60.95 | 1.09 | 1.03 | Male |
| 5 | Ethiopia | SSA | 61.62 | 67.13 | 1.09 | 1.03 | Male |
| 5 | Gabon | SSA | 63.26 | 67.55 | 1.07 | 1.01 | Male |
| 5 | Gambia | SSA | 61.57 | 64.04 | 1.04 | 0.98 | Female |
| 5 | Ghana | SSA | 60.77 | 64.47 | 1.06 | 1 | None |
| 5 | Guinea | SSA | 60.22 | 61.78 | 1.03 | 0.97 | Female |
| 5 | Guinea-Bissau | SSA | 60.43 | 64.3 | 1.06 | 1.01 | None |
| 5 | Kenya | SSA | 58.79 | 62.82 | 1.07 | 1.01 | Male |
| 5 | Lesotho | SSA | 52.41 | 56.97 | 1.09 | 1.03 | Male |
| 5 | Liberia | SSA | 60.54 | 62.3 | 1.03 | 0.97 | Female |
| 5 | Madagascar | SSA | 61.59 | 64.27 | 1.04 | 0.99 | Female |
| 5 | Malawi | SSA | 59.8 | 66.36 | 1.11 | 1.05 | Male |
| 5 | Mali | SSA | 59.97 | 61.85 | 1.03 | 0.97 | Female |
| 5 | Mauritania | SSA | 64.05 | 67.3 | 1.05 | 0.99 | Female |
| 5 | Mauritius | SSA | 67.29 | 73.64 | 1.09 | 1.03 | Male |
| 5 | Mozambique | SSA | 58.37 | 63.35 | 1.09 | 1.03 | Male |
| 5 | Namibia | SSA | 57.52 | 64.97 | 1.13 | 1.07 | Male |
| 5 | Niger | SSA | 62.46 | 64 | 1.02 | 0.97 | Female |
| 5 | Nigeria | SSA | 55 | 54.69 | 0.99 | 0.94 | Female |
| 5 | Rwanda | SSA | 62.34 | 66.55 | 1.07 | 1.01 | Male |
| 5 | Senegal | SSA | 64 | 67.26 | 1.05 | 0.99 | Female |
| 5 | Sierra Leone | SSA | 61.1 | 62.92 | 1.03 | 0.97 | Female |
| 5 | Somalia | SSA | 57.88 | 61.59 | 1.06 | 1.01 | None |
| 5 | South Africa | SSA | 60.22 | 66.64 | 1.11 | 1.05 | Male |
| 5 | South Sudan | SSA | 57.08 | 61.67 | 1.08 | 1.02 | Male |
| 5 | Sudan | SSA | 62.34 | 67.35 | 1.08 | 1.02 | Male |
| 5 | Togo | SSA | 60.52 | 60.13 | 0.99 | 0.94 | Female |
| 5 | Uganda | SSA | 61.69 | 67.08 | 1.09 | 1.03 | Male |
| 5 | United Republic Of Tanzania | SSA | 61.34 | 66.92 | 1.09 | 1.03 | Male |
| 5 | Zambia | SSA | 59.56 | 64.24 | 1.08 | 1.02 | Male |
| 5 | Zimbabwe | SSA | 57.26 | 61.2 | 1.07 | 1.01 | Male |
| 5 | United States Of America | CIU | 71.99 | 76.95 | 1.07 | 1.01 | Male |
| 5 | Australia | WPSA | 76.55 | 80.56 | 1.05 | 0.99 | None |
| 5 | Bangladesh | WPSA | 68.42 | 71.71 | 1.05 | 0.99 | Female |
| 5 | Bhutan | WPSA | 67.47 | 70.67 | 1.05 | 0.99 | Female |
| 5 | Brunei Darussalam | WPSA | 68.84 | 72.99 | 1.06 | 1 | None |
| 5 | Cambodia | WPSA | 64.57 | 69.29 | 1.07 | 1.01 | Male |
| 5 | Dem. People's Republic Of Korea | WPSA | 66.33 | 72.07 | 1.09 | 1.03 | Male |
| 5 | Fiji | WPSA | 61.94 | 65.59 | 1.06 | 1 | None |
| 5 | Indonesia | WPSA | 65.14 | 68.94 | 1.06 | 1 | None |
| 5 | Japan | WPSA | 76.58 | 82.64 | 1.08 | 1.02 | Male |
| 5 | Lao People's Democratic Republic | WPSA | 64.2 | 68.08 | 1.06 | 1 | None |
| 5 | Malaysia | WPSA | 69.18 | 74.18 | 1.07 | 1.01 | Male |
| 5 | Maldives | WPSA | 73.99 | 77.04 | 1.04 | 0.98 | Female |
| 5 | Myanmar | WPSA | 61.79 | 67.36 | 1.09 | 1.03 | Male |
| 5 | Nepal | WPSA | 65.21 | 67.81 | 1.04 | 0.98 | Female |
| 5 | New Zealand | WPSA | 75.47 | 79.09 | 1.05 | 0.99 | Female |
| 5 | Papua New Guinea | WPSA | 61.1 | 66.41 | 1.09 | 1.03 | Male |
| 5 | Philippines | WPSA | 63.77 | 69.58 | 1.09 | 1.03 | Male |
| 5 | Republic Of Korea | WPSA | 75.61 | 81.98 | 1.08 | 1.02 | Male |
| 5 | Singapore | WPSA | 76.67 | 80.99 | 1.06 | 1 | None |
| 5 | Solomon Islands | WPSA | 65.27 | 67.68 | 1.04 | 0.98 | Female |
| 5 | Sri Lanka | WPSA | 69.02 | 75.42 | 1.09 | 1.03 | Male |
| 5 | Taiwan | WPSA | 72.75 | 79.42 | 1.09 | 1.03 | Male |
| 5 | Thailand | WPSA | 68.57 | 77.42 | 1.13 | 1.07 | Male |
| 5 | Timor-Leste | WPSA | 63.81 | 66.75 | 1.05 | 0.99 | Female |
| 5 | Vanuatu | WPSA | 65.29 | 69.64 | 1.07 | 1.01 | Male |
| 5 | Viet Nam | WPSA | 66.14 | 75.36 | 1.14 | 1.08 | Male |
| 15 | American Samoa |  | 56.04 | 61.58 | 1.1 | 1.03 | Male |
| 15 | Andorra |  | 67.75 | 71.69 | 1.06 | 0.99 | Female |
| 15 | Anguilla |  | 60.84 | 67.58 | 1.11 | 1.04 | Male |
| 15 | Antigua And Barbuda |  | 60.09 | 65.75 | 1.09 | 1.03 | Male |
| 15 | Aruba |  | 59.46 | 65.05 | 1.09 | 1.03 | Male |
| 15 | Barbados |  | 60.39 | 64.49 | 1.07 | 1 | None |
| 15 | Bermuda |  | 63.01 | 70.24 | 1.11 | 1.05 | Male |
| 15 | Bonaire, Sint Eustatius And Saba |  | 60.98 | 66.49 | 1.09 | 1.02 | Male |
| 15 | British Virgin Islands |  | 60.52 | 65.65 | 1.08 | 1.02 | Male |
| 15 | Cayman Islands |  | 61.95 | 66.87 | 1.08 | 1.01 | Male |
| 15 | China, Hong Kong Sar |  | 67.52 | 73.46 | 1.09 | 1.02 | Male |
| 15 | China, Macao Sar |  | 66.01 | 71.19 | 1.08 | 1.01 | Male |
| 15 | Cook Islands |  | 56.82 | 64.15 | 1.13 | 1.06 | Male |
| 15 | Curaçao |  | 58.4 | 66.39 | 1.14 | 1.07 | Male |
| 15 | Dominica |  | 56.15 | 61.86 | 1.1 | 1.03 | Male |
| 15 | Falkland Islands (Malvinas) |  | 62.64 | 66.67 | 1.06 | 1 | None |
| 15 | Faroe Islands |  | 63.74 | 67.75 | 1.06 | 1 | None |
| 15 | French Guiana |  | 59.86 | 65.65 | 1.1 | 1.03 | Male |
| 15 | French Polynesia |  | 66.51 | 71.38 | 1.07 | 1.01 | None |
| 15 | Gibraltar |  | 66.73 | 71.73 | 1.07 | 1.01 | Male |
| 15 | Greenland |  | 55.09 | 59.02 | 1.07 | 1 | None |
| 15 | Grenada |  | 58.53 | 64.57 | 1.1 | 1.03 | Male |
| 15 | Guadeloupe |  | 63.33 | 70.72 | 1.12 | 1.05 | Male |
| 15 | Guam |  | 59.09 | 67 | 1.13 | 1.06 | Male |
| 15 | Guernsey |  | 66.75 | 71.05 | 1.06 | 1 | None |
| 15 | Holy See |  | 67.08 | 71.23 | 1.06 | 1 | None |
| 15 | Isle Of Man |  | 65.12 | 69.06 | 1.06 | 0.99 | None |
| 15 | Jersey |  | 63.55 | 68.14 | 1.07 | 1.01 | None |
| 15 | Kiribati |  | 53.98 | 56.82 | 1.05 | 0.99 | Female |
| 15 | Kosovo (Under Unsc Res. 1244) |  | 61.07 | 65.25 | 1.07 | 1 | None |
| 15 | Liechtenstein |  | 67.09 | 70.87 | 1.06 | 0.99 | Female |
| 15 | Marshall Islands |  | 51.87 | 55.93 | 1.08 | 1.01 | Male |
| 15 | Martinique |  | 64.68 | 70.8 | 1.09 | 1.03 | Male |
| 15 | Mayotte |  | 60.33 | 64.02 | 1.06 | 1 | None |
| 15 | Micronesia (Fed. States Of) |  | 50.33 | 56.61 | 1.12 | 1.05 | Male |
| 15 | Monaco |  | 69.67 | 73.78 | 1.06 | 0.99 | None |
| 15 | Montserrat |  | 59.3 | 63.92 | 1.08 | 1.01 | Male |
| 15 | Nauru |  | 46.45 | 51.62 | 1.11 | 1.04 | Male |
| 15 | New Caledonia |  | 60.29 | 66.46 | 1.1 | 1.03 | Male |
| 15 | Niue |  | 54.04 | 59.24 | 1.1 | 1.03 | Male |
| 15 | Northern Mariana Islands |  | 61.89 | 65.49 | 1.06 | 0.99 | Female |
| 15 | Palau |  | 53.49 | 58.85 | 1.1 | 1.03 | Male |
| 15 | Puerto Rico |  | 63.31 | 70.76 | 1.12 | 1.05 | Male |
| 15 | Réunion |  | 64.6 | 71 | 1.1 | 1.03 | Male |
| 15 | Saint Barthélemy |  | 65.66 | 73.07 | 1.11 | 1.04 | Male |
| 15 | Saint Helena |  | 60.32 | 66.52 | 1.1 | 1.03 | Male |
| 15 | Saint Kitts And Nevis |  | 54.54 | 61.82 | 1.13 | 1.06 | Male |
| 15 | Saint Lucia |  | 55.33 | 62.47 | 1.13 | 1.06 | Male |
| 15 | Saint Martin (French Part) |  | 62.49 | 69.6 | 1.11 | 1.04 | Male |
| 15 | Saint Pierre And Miquelon |  | 58.68 | 67.02 | 1.14 | 1.07 | Male |
| 15 | Saint Vincent And The Grenadines |  | 54.71 | 60.53 | 1.11 | 1.04 | Male |
| 15 | Samoa |  | 55.34 | 58.98 | 1.07 | 1 | None |
| 15 | San Marino |  | 68.77 | 71.95 | 1.05 | 0.98 | Female |
| 15 | Sao Tome And Principe |  | 51.27 | 58.41 | 1.14 | 1.07 | Male |
| 15 | Seychelles |  | 55.43 | 62.01 | 1.12 | 1.05 | Male |
| 15 | Sint Maarten (Dutch Part) |  | 59.71 | 64.81 | 1.09 | 1.02 | Male |
| 15 | State Of Palestine |  | 60.08 | 64.39 | 1.07 | 1.01 | None |
| 15 | Tokelau |  | 60.11 | 63.9 | 1.06 | 1 | None |
| 15 | Tonga |  | 54.96 | 61.95 | 1.13 | 1.06 | Male |
| 15 | Turks And Caicos Islands |  | 61.2 | 65.52 | 1.07 | 1 | None |
| 15 | Tuvalu |  | 49.86 | 56.61 | 1.14 | 1.06 | Male |
| 15 | United States Virgin Islands |  | 55.94 | 66.46 | 1.19 | 1.11 | Male |
| 15 | Wallis And Futuna Islands |  | 62.88 | 65.53 | 1.04 | 0.98 | Female |
| 15 | Western Sahara |  | 56.72 | 59.74 | 1.05 | 0.99 | Female |
| 15 | Albania | CEE | 63.78 | 66.95 | 1.05 | 0.98 | Female |
| 15 | Armenia | CEE | 57.08 | 64.78 | 1.13 | 1.06 | Male |
| 15 | Belarus | CEE | 54.54 | 64.28 | 1.18 | 1.11 | Male |
| 15 | Bosnia And Herzegovina | CEE | 59.49 | 65.93 | 1.11 | 1.04 | Male |
| 15 | Bulgaria | CEE | 57.18 | 64.35 | 1.13 | 1.06 | Male |
| 15 | Croatia | CEE | 60.33 | 66.79 | 1.11 | 1.04 | Male |
| 15 | Czechia | CEE | 61.6 | 67.36 | 1.09 | 1.03 | Male |
| 15 | Estonia | CEE | 59.71 | 67.93 | 1.14 | 1.07 | Male |
| 15 | Georgia | CEE | 55.11 | 64.67 | 1.17 | 1.1 | Male |
| 15 | Hungary | CEE | 58.5 | 65.06 | 1.11 | 1.04 | Male |
| 15 | Latvia | CEE | 56.24 | 65.43 | 1.16 | 1.09 | Male |
| 15 | Lithuania | CEE | 56.88 | 66.36 | 1.17 | 1.09 | Male |
| 15 | Montenegro | CEE | 58.72 | 65.38 | 1.11 | 1.04 | Male |
| 15 | North Macedonia | CEE | 60.17 | 64.62 | 1.07 | 1.01 | Male |
| 15 | Poland | CEE | 59.49 | 67.15 | 1.13 | 1.06 | Male |
| 15 | Republic Of Moldova | CEE | 51.83 | 60.76 | 1.17 | 1.1 | Male |
| 15 | Romania | CEE | 57.38 | 64.63 | 1.13 | 1.06 | Male |
| 15 | Russian Federation | CEE | 53.31 | 63.69 | 1.19 | 1.12 | Male |
| 15 | Serbia | CEE | 58.35 | 64.82 | 1.11 | 1.04 | Male |
| 15 | Slovakia | CEE | 59.85 | 66.54 | 1.11 | 1.04 | Male |
| 15 | Slovenia | CEE | 63.49 | 69.04 | 1.09 | 1.02 | Male |
| 15 | Ukraine | CEE | 54.54 | 64.38 | 1.18 | 1.11 | Male |
| 15 | Afghanistan | CA | 49.89 | 55.98 | 1.12 | 1.05 | Male |
| 15 | Azerbaijan | CA | 57.11 | 62.89 | 1.1 | 1.03 | Male |
| 15 | Kazakhstan | CA | 55.2 | 63.59 | 1.15 | 1.08 | Male |
| 15 | Kyrgyzstan | CA | 54.04 | 61.1 | 1.13 | 1.06 | Male |
| 15 | Mongolia | CA | 52.56 | 61.67 | 1.17 | 1.1 | Male |
| 15 | Pakistan | CA | 54.98 | 59.11 | 1.08 | 1.01 | Male |
| 15 | Tajikistan | CA | 56.65 | 60.55 | 1.07 | 1 | None |
| 15 | Turkmenistan | CA | 54.7 | 60.21 | 1.1 | 1.03 | Male |
| 15 | Uzbekistan | CA | 55.46 | 61.63 | 1.11 | 1.04 | Male |
| 15 | China | CIU | 61.18 | 66.55 | 1.09 | 1.02 | Male |
| 15 | India | CIU | 57.05 | 60.23 | 1.06 | 0.99 | Female |
| 15 | Argentina | LAC | 60.22 | 65.42 | 1.09 | 1.02 | Male |
| 15 | Bahamas | LAC | 56.12 | 61.77 | 1.1 | 1.03 | Male |
| 15 | Belize | LAC | 56.02 | 61.67 | 1.1 | 1.03 | Male |
| 15 | Bolivia (Plurinational State Of) | LAC | 54.71 | 59.09 | 1.08 | 1.01 | Male |
| 15 | Brazil | LAC | 59.04 | 65.21 | 1.1 | 1.04 | Male |
| 15 | Chile | LAC | 63.76 | 68.15 | 1.07 | 1 | None |
| 15 | Colombia | LAC | 60.25 | 65.87 | 1.09 | 1.03 | Male |
| 15 | Costa Rica | LAC | 63.43 | 68.71 | 1.08 | 1.02 | Male |
| 15 | Cuba | LAC | 60.7 | 65.46 | 1.08 | 1.01 | Male |
| 15 | Dominican Republic | LAC | 57.67 | 63.99 | 1.11 | 1.04 | Male |
| 15 | Ecuador | LAC | 60.97 | 66.15 | 1.08 | 1.02 | Male |
| 15 | El Salvador | LAC | 53.28 | 62.09 | 1.17 | 1.09 | Male |
| 15 | Guatemala | LAC | 56.44 | 60.9 | 1.08 | 1.01 | Male |
| 15 | Guyana | LAC | 52.93 | 59.9 | 1.13 | 1.06 | Male |
| 15 | Haiti | LAC | 51.79 | 56.83 | 1.1 | 1.03 | Male |
| 15 | Honduras | LAC | 56.3 | 61.16 | 1.09 | 1.02 | Male |
| 15 | Jamaica | LAC | 55.82 | 60.47 | 1.08 | 1.02 | Male |
| 15 | Mexico | LAC | 57.9 | 63.58 | 1.1 | 1.03 | Male |
| 15 | Nicaragua | LAC | 57.55 | 62.79 | 1.09 | 1.02 | Male |
| 15 | Panama | LAC | 62.24 | 68.08 | 1.09 | 1.03 | Male |
| 15 | Paraguay | LAC | 57.47 | 63.32 | 1.1 | 1.03 | Male |
| 15 | Peru | LAC | 60.67 | 64.94 | 1.07 | 1 | None |
| 15 | Suriname | LAC | 55.27 | 61.59 | 1.11 | 1.04 | Male |
| 15 | Trinidad And Tobago | LAC | 56.28 | 62.4 | 1.11 | 1.04 | Male |
| 15 | Uruguay | LAC | 59.31 | 66.98 | 1.13 | 1.06 | Male |
| 15 | Venezuela (Bolivarian Republic Of) | LAC | 55.72 | 63.19 | 1.13 | 1.06 | Male |
| 15 | Algeria | MENA | 61.48 | 63.99 | 1.04 | 0.98 | Female |
| 15 | Bahrain | MENA | 65.36 | 67.21 | 1.03 | 0.96 | Female |
| 15 | Egypt | MENA | 55.84 | 60.04 | 1.08 | 1.01 | Male |
| 15 | Iran (Islamic Republic Of) | MENA | 61.35 | 65.11 | 1.06 | 1 | None |
| 15 | Iraq | MENA | 56.72 | 60.04 | 1.06 | 0.99 | None |
| 15 | Israel | MENA | 66.38 | 70.03 | 1.05 | 0.99 | Female |
| 15 | Jordan | MENA | 61.25 | 65.48 | 1.07 | 1 | None |
| 15 | Kuwait | MENA | 64.88 | 67.37 | 1.04 | 0.97 | Female |
| 15 | Lebanon | MENA | 62.56 | 66.29 | 1.06 | 0.99 | None |
| 15 | Libya | MENA | 56.26 | 62.3 | 1.11 | 1.04 | Male |
| 15 | Morocco | MENA | 59.03 | 62.91 | 1.07 | 1 | None |
| 15 | Oman | MENA | 64.59 | 67.84 | 1.05 | 0.99 | Female |
| 15 | Qatar | MENA | 67.87 | 69.64 | 1.03 | 0.96 | Female |
| 15 | Saudi Arabia | MENA | 62.75 | 66.11 | 1.05 | 0.99 | Female |
| 15 | Syrian Arab Republic | MENA | 55.07 | 61.28 | 1.11 | 1.04 | Male |
| 15 | Tunisia | MENA | 59.39 | 64.44 | 1.09 | 1.02 | Male |
| 15 | Türkiye | MENA | 60.62 | 66.77 | 1.1 | 1.03 | Male |
| 15 | United Arab Emirates | MENA | 67.27 | 69.63 | 1.04 | 0.97 | Female |
| 15 | Yemen | MENA | 52.14 | 58.57 | 1.12 | 1.05 | Male |
| 15 | Austria | NA | 64.93 | 69.52 | 1.07 | 1 | None |
| 15 | Belgium | NA | 65.02 | 69.36 | 1.07 | 1 | None |
| 15 | Canada | NA | 65.69 | 69.81 | 1.06 | 1 | None |
| 15 | Cyprus | NA | 64.63 | 68.92 | 1.07 | 1 | None |
| 15 | Denmark | NA | 64.8 | 68.71 | 1.06 | 0.99 | None |
| 15 | Finland | NA | 64.45 | 69.75 | 1.08 | 1.01 | Male |
| 15 | France | NA | 65.2 | 70.95 | 1.09 | 1.02 | Male |
| 15 | Germany | NA | 64.21 | 68.88 | 1.07 | 1.01 | None |
| 15 | Greece | NA | 64.13 | 69.22 | 1.08 | 1.01 | Male |
| 15 | Iceland | NA | 66.59 | 69.53 | 1.04 | 0.98 | Female |
| 15 | Ireland | NA | 65.79 | 69.71 | 1.06 | 0.99 | None |
| 15 | Italy | NA | 66.45 | 70.67 | 1.06 | 1 | None |
| 15 | Luxembourg | NA | 65.55 | 69.28 | 1.06 | 0.99 | Female |
| 15 | Malta | NA | 66.43 | 70.82 | 1.07 | 1 | None |
| 15 | Netherlands | NA | 65.88 | 68.92 | 1.05 | 0.98 | Female |
| 15 | Norway | NA | 66.48 | 69.92 | 1.05 | 0.99 | Female |
| 15 | Portugal | NA | 64.34 | 70.08 | 1.09 | 1.02 | Male |
| 15 | Spain | NA | 66.07 | 71.43 | 1.08 | 1.01 | Male |
| 15 | Sweden | NA | 66.6 | 69.98 | 1.05 | 0.99 | Female |
| 15 | Switzerland | NA | 67.27 | 70.92 | 1.05 | 0.99 | Female |
| 15 | United Kingdom | NA | 64.99 | 68.66 | 1.06 | 0.99 | Female |
| 15 | Angola | SSA | 51.66 | 55.98 | 1.08 | 1.02 | Male |
| 15 | Benin | SSA | 51.43 | 53.31 | 1.04 | 0.97 | Female |
| 15 | Botswana | SSA | 53.35 | 57.86 | 1.08 | 1.02 | Male |
| 15 | Burkina Faso | SSA | 50.49 | 53.78 | 1.07 | 1 | None |
| 15 | Burundi | SSA | 51 | 54.19 | 1.06 | 1 | None |
| 15 | Cabo Verde | SSA | 58.38 | 64.8 | 1.11 | 1.04 | Male |
| 15 | Cameroon | SSA | 50.91 | 54.52 | 1.07 | 1 | None |
| 15 | Central African Republic | SSA | 22.19 | 39.11 | 1.76 | 1.65 | Male |
| 15 | Chad | SSA | 46.28 | 49.05 | 1.06 | 0.99 | None |
| 15 | Comoros | SSA | 53.15 | 56.8 | 1.07 | 1 | None |
| 15 | Congo | SSA | 50.88 | 53.64 | 1.05 | 0.99 | Female |
| 15 | Côte D'ivoire | SSA | 49.65 | 52.46 | 1.06 | 0.99 | Female |
| 15 | Democratic Republic Of The Congo | SSA | 50.91 | 54.01 | 1.06 | 0.99 | None |
| 15 | Djibouti | SSA | 52.54 | 56.65 | 1.08 | 1.01 | Male |
| 15 | Equatorial Guinea | SSA | 52.26 | 55.1 | 1.05 | 0.99 | Female |
| 15 | Eritrea | SSA | 54.37 | 57.8 | 1.06 | 1 | None |
| 15 | Eswatini | SSA | 46.33 | 51.52 | 1.11 | 1.04 | Male |
| 15 | Ethiopia | SSA | 53.02 | 58.02 | 1.09 | 1.03 | Male |
| 15 | Gabon | SSA | 53.77 | 58.02 | 1.08 | 1.01 | Male |
| 15 | Gambia | SSA | 52.98 | 55.45 | 1.05 | 0.98 | Female |
| 15 | Ghana | SSA | 52.3 | 55.8 | 1.07 | 1 | None |
| 15 | Guinea | SSA | 51.89 | 53.62 | 1.03 | 0.97 | Female |
| 15 | Guinea-Bissau | SSA | 51.39 | 55.19 | 1.07 | 1.01 | Male |
| 15 | Kenya | SSA | 49.31 | 53.33 | 1.08 | 1.01 | Male |
| 15 | Lesotho | SSA | 43.11 | 47.63 | 1.1 | 1.04 | Male |
| 15 | Liberia | SSA | 51.31 | 53.02 | 1.03 | 0.97 | Female |
| 15 | Madagascar | SSA | 53 | 55.64 | 1.05 | 0.98 | Female |
| 15 | Malawi | SSA | 50.33 | 56.85 | 1.13 | 1.06 | Male |
| 15 | Mali | SSA | 51.17 | 53.27 | 1.04 | 0.98 | Female |
| 15 | Mauritania | SSA | 54.53 | 57.76 | 1.06 | 0.99 | None |
| 15 | Mauritius | SSA | 57.49 | 63.74 | 1.11 | 1.04 | Male |
| 15 | Mozambique | SSA | 49.11 | 54.06 | 1.1 | 1.03 | Male |
| 15 | Namibia | SSA | 48.01 | 55.46 | 1.16 | 1.08 | Male |
| 15 | Niger | SSA | 53.33 | 55.08 | 1.03 | 0.97 | Female |
| 15 | Nigeria | SSA | 47.62 | 48.08 | 1.01 | 0.95 | Female |
| 15 | Rwanda | SSA | 52.85 | 57.01 | 1.08 | 1.01 | Male |
| 15 | Senegal | SSA | 54.69 | 57.91 | 1.06 | 0.99 | None |
| 15 | Sierra Leone | SSA | 52.21 | 53.97 | 1.03 | 0.97 | Female |
| 15 | Somalia | SSA | 49.78 | 53.46 | 1.07 | 1.01 | Male |
| 15 | South Africa | SSA | 50.59 | 56.94 | 1.13 | 1.06 | Male |
| 15 | South Sudan | SSA | 49.18 | 53.52 | 1.09 | 1.02 | Male |
| 15 | Sudan | SSA | 53.62 | 58.19 | 1.09 | 1.02 | Male |
| 15 | Togo | SSA | 51.66 | 51.83 | 1 | 0.94 | Female |
| 15 | Uganda | SSA | 52.23 | 57.6 | 1.1 | 1.03 | Male |
| 15 | United Republic Of Tanzania | SSA | 51.86 | 57.42 | 1.11 | 1.04 | Male |
| 15 | Zambia | SSA | 50.32 | 54.93 | 1.09 | 1.02 | Male |
| 15 | Zimbabwe | SSA | 47.9 | 51.82 | 1.08 | 1.01 | Male |
| 15 | United States Of America | CIU | 62.09 | 67.03 | 1.08 | 1.01 | Male |
| 15 | Australia | WPSA | 66.61 | 70.61 | 1.06 | 0.99 | None |
| 15 | Bangladesh | WPSA | 58.87 | 62.16 | 1.06 | 0.99 | Female |
| 15 | Bhutan | WPSA | 57.77 | 60.9 | 1.05 | 0.99 | Female |
| 15 | Brunei Darussalam | WPSA | 58.99 | 63.15 | 1.07 | 1 | None |
| 15 | Cambodia | WPSA | 55.48 | 59.83 | 1.08 | 1.01 | Male |
| 15 | Dem. People's Republic Of Korea | WPSA | 56.9 | 62.41 | 1.1 | 1.03 | Male |
| 15 | Fiji | WPSA | 52.18 | 55.82 | 1.07 | 1 | None |
| 15 | Indonesia | WPSA | 55.45 | 59.27 | 1.07 | 1 | None |
| 15 | Japan | WPSA | 66.64 | 72.7 | 1.09 | 1.02 | Male |
| 15 | Lao People's Democratic Republic | WPSA | 54.76 | 58.46 | 1.07 | 1 | None |
| 15 | Malaysia | WPSA | 59.36 | 64.3 | 1.08 | 1.02 | Male |
| 15 | Maldives | WPSA | 64.05 | 67.14 | 1.05 | 0.98 | Female |
| 15 | Myanmar | WPSA | 52.58 | 57.8 | 1.1 | 1.03 | Male |
| 15 | Nepal | WPSA | 55.67 | 58.21 | 1.05 | 0.98 | Female |
| 15 | New Zealand | WPSA | 65.56 | 69.17 | 1.05 | 0.99 | Female |
| 15 | Papua New Guinea | WPSA | 51.68 | 56.93 | 1.1 | 1.03 | Male |
| 15 | Philippines | WPSA | 54.09 | 59.87 | 1.11 | 1.04 | Male |
| 15 | Republic Of Korea | WPSA | 65.67 | 72.04 | 1.1 | 1.03 | Male |
| 15 | Singapore | WPSA | 66.73 | 71.04 | 1.06 | 1 | None |
| 15 | Solomon Islands | WPSA | 55.72 | 58.08 | 1.04 | 0.98 | Female |
| 15 | Sri Lanka | WPSA | 59.14 | 65.5 | 1.11 | 1.04 | Male |
| 15 | Taiwan | WPSA | 62.83 | 69.5 | 1.11 | 1.04 | Male |
| 15 | Thailand | WPSA | 58.87 | 67.63 | 1.15 | 1.08 | Male |
| 15 | Timor-Leste | WPSA | 54.41 | 57.25 | 1.05 | 0.99 | Female |
| 15 | Vanuatu | WPSA | 55.56 | 59.93 | 1.08 | 1.01 | Male |
| 15 | Viet Nam | WPSA | 56.83 | 65.62 | 1.15 | 1.08 | Male |
| 35 | American Samoa |  | 37.43 | 42.25 | 1.13 | 1.04 | Male |
| 35 | Andorra |  | 48.02 | 51.92 | 1.08 | 1 | None |
| 35 | Anguilla |  | 41.64 | 47.86 | 1.15 | 1.06 | Male |
| 35 | Antigua And Barbuda |  | 41.29 | 46.46 | 1.13 | 1.04 | Male |
| 35 | Aruba |  | 40.43 | 45.48 | 1.12 | 1.04 | Male |
| 35 | Barbados |  | 41.37 | 45.15 | 1.09 | 1 | None |
| 35 | Bermuda |  | 43.73 | 50.57 | 1.16 | 1.06 | Male |
| 35 | Bonaire, Sint Eustatius And Saba |  | 41.58 | 46.92 | 1.13 | 1.04 | Male |
| 35 | British Virgin Islands |  | 41.24 | 45.88 | 1.11 | 1.02 | Male |
| 35 | Cayman Islands |  | 43.09 | 47.83 | 1.11 | 1.02 | Male |
| 35 | China, Hong Kong Sar |  | 47.98 | 53.7 | 1.12 | 1.03 | Male |
| 35 | China, Macao Sar |  | 46.25 | 51.34 | 1.11 | 1.02 | Male |
| 35 | Cook Islands |  | 38.32 | 44.77 | 1.17 | 1.08 | Male |
| 35 | Curaçao |  | 39.33 | 46.82 | 1.19 | 1.1 | Male |
| 35 | Dominica |  | 37.53 | 42.4 | 1.13 | 1.04 | Male |
| 35 | Falkland Islands (Malvinas) |  | 43.12 | 47.09 | 1.09 | 1.01 | None |
| 35 | Faroe Islands |  | 44.15 | 47.9 | 1.08 | 1 | None |
| 35 | French Guiana |  | 41.17 | 46.36 | 1.13 | 1.04 | Male |
| 35 | French Polynesia |  | 46.82 | 51.61 | 1.1 | 1.01 | Male |
| 35 | Gibraltar |  | 47.59 | 52.18 | 1.1 | 1.01 | None |
| 35 | Greenland |  | 37.9 | 40.79 | 1.08 | 0.99 | None |
| 35 | Grenada |  | 39.51 | 45.29 | 1.15 | 1.06 | Male |
| 35 | Guadeloupe |  | 44.09 | 51.04 | 1.16 | 1.07 | Male |
| 35 | Guam |  | 40.91 | 47.74 | 1.17 | 1.07 | Male |
| 35 | Guernsey |  | 47.06 | 51.3 | 1.09 | 1 | None |
| 35 | Holy See |  | 47.37 | 51.47 | 1.09 | 1 | None |
| 35 | Isle Of Man |  | 46.11 | 49.67 | 1.08 | 0.99 | None |
| 35 | Jersey |  | 43.98 | 48.49 | 1.1 | 1.02 | Male |
| 35 | Kiribati |  | 35.99 | 38.56 | 1.07 | 0.99 | Female |
| 35 | Kosovo (Under Unsc Res. 1244) |  | 41.51 | 45.51 | 1.1 | 1.01 | None |
| 35 | Liechtenstein |  | 47.38 | 51.13 | 1.08 | 0.99 | None |
| 35 | Marshall Islands |  | 34.12 | 38.04 | 1.11 | 1.03 | Male |
| 35 | Martinique |  | 45.21 | 51.05 | 1.13 | 1.04 | Male |
| 35 | Mayotte |  | 41.13 | 44.53 | 1.08 | 1 | None |
| 35 | Micronesia (Fed. States Of) |  | 32.92 | 38.59 | 1.17 | 1.08 | Male |
| 35 | Monaco |  | 49.88 | 53.95 | 1.08 | 1 | None |
| 35 | Montserrat |  | 40.09 | 44.47 | 1.11 | 1.02 | Male |
| 35 | Nauru |  | 29.98 | 35.39 | 1.18 | 1.09 | Male |
| 35 | New Caledonia |  | 43.9 | 47.05 | 1.07 | 0.99 | Female |
| 35 | Niue |  | 35.82 | 40.41 | 1.13 | 1.04 | Male |
| 35 | Northern Mariana Islands |  | 42.57 | 45.88 | 1.08 | 0.99 | None |
| 35 | Palau |  | 35.38 | 40.22 | 1.14 | 1.05 | Male |
| 35 | Puerto Rico |  | 44.23 | 51 | 1.15 | 1.06 | Male |
| 35 | Réunion |  | 45.39 | 51.25 | 1.13 | 1.04 | Male |
| 35 | Saint Barthélemy |  | 46 | 53.26 | 1.16 | 1.07 | Male |
| 35 | Saint Helena |  | 40.99 | 46.95 | 1.15 | 1.05 | Male |
| 35 | Saint Kitts And Nevis |  | 36.25 | 42.59 | 1.17 | 1.08 | Male |
| 35 | Saint Lucia |  | 36.88 | 43.1 | 1.17 | 1.08 | Male |
| 35 | Saint Martin (French Part) |  | 42.98 | 49.9 | 1.16 | 1.07 | Male |
| 35 | Saint Pierre And Miquelon |  | 39.71 | 47.21 | 1.19 | 1.09 | Male |
| 35 | Saint Vincent And The Grenadines |  | 36.91 | 41.53 | 1.13 | 1.04 | Male |
| 35 | Samoa |  | 36.98 | 40.51 | 1.1 | 1.01 | None |
| 35 | San Marino |  | 49.09 | 52.11 | 1.06 | 0.98 | Female |
| 35 | Sao Tome And Principe |  | 33.66 | 40.04 | 1.19 | 1.09 | Male |
| 35 | Seychelles |  | 36.94 | 43 | 1.16 | 1.07 | Male |
| 35 | Sint Maarten (Dutch Part) |  | 40.45 | 45.32 | 1.12 | 1.03 | Male |
| 35 | State Of Palestine |  | 40.82 | 44.92 | 1.1 | 1.01 | Male |
| 35 | Tokelau |  | 41.05 | 44.82 | 1.09 | 1 | None |
| 35 | Tonga |  | 36.65 | 42.87 | 1.17 | 1.08 | Male |
| 35 | Turks And Caicos Islands |  | 42.01 | 46.02 | 1.1 | 1.01 | None |
| 35 | Tuvalu |  | 32.55 | 38.68 | 1.19 | 1.09 | Male |
| 35 | United States Virgin Islands |  | 37.43 | 46.89 | 1.25 | 1.15 | Male |
| 35 | Wallis And Futuna Islands |  | 43.22 | 45.97 | 1.06 | 0.98 | Female |
| 35 | Western Sahara |  | 38 | 40.71 | 1.07 | 0.99 | Female |
| 35 | Albania | CEE | 44.4 | 47.31 | 1.07 | 0.98 | Female |
| 35 | Armenia | CEE | 37.86 | 45.07 | 1.19 | 1.1 | Male |
| 35 | Belarus | CEE | 35.52 | 44.67 | 1.26 | 1.16 | Male |
| 35 | Bosnia And Herzegovina | CEE | 40.11 | 46.24 | 1.15 | 1.06 | Male |
| 35 | Bulgaria | CEE | 38.07 | 44.75 | 1.18 | 1.08 | Male |
| 35 | Croatia | CEE | 40.97 | 47.05 | 1.15 | 1.06 | Male |
| 35 | Czechia | CEE | 42.3 | 47.68 | 1.13 | 1.04 | Male |
| 35 | Estonia | CEE | 40.5 | 48.39 | 1.19 | 1.1 | Male |
| 35 | Georgia | CEE | 36.24 | 45.11 | 1.24 | 1.15 | Male |
| 35 | Hungary | CEE | 39.07 | 45.35 | 1.16 | 1.07 | Male |
| 35 | Latvia | CEE | 37.49 | 45.96 | 1.23 | 1.13 | Male |
| 35 | Lithuania | CEE | 38.04 | 46.84 | 1.23 | 1.13 | Male |
| 35 | Montenegro | CEE | 39.52 | 45.78 | 1.16 | 1.07 | Male |
| 35 | North Macedonia | CEE | 40.66 | 44.88 | 1.1 | 1.02 | Male |
| 35 | Poland | CEE | 40.43 | 47.45 | 1.17 | 1.08 | Male |
| 35 | Republic Of Moldova | CEE | 33.17 | 41.3 | 1.25 | 1.15 | Male |
| 35 | Romania | CEE | 38.19 | 45.01 | 1.18 | 1.08 | Male |
| 35 | Russian Federation | CEE | 34.83 | 44.4 | 1.27 | 1.17 | Male |
| 35 | Serbia | CEE | 38.99 | 45.13 | 1.16 | 1.07 | Male |
| 35 | Slovakia | CEE | 40.59 | 46.89 | 1.16 | 1.06 | Male |
| 35 | Slovenia | CEE | 44.1 | 49.24 | 1.12 | 1.03 | Male |
| 35 | Ukraine | CEE | 35.87 | 44.91 | 1.25 | 1.15 | Male |
| 35 | Afghanistan | CA | 34.18 | 37.99 | 1.11 | 1.02 | Male |
| 35 | Azerbaijan | CA | 38.1 | 43.41 | 1.14 | 1.05 | Male |
| 35 | Kazakhstan | CA | 36.52 | 44.2 | 1.21 | 1.11 | Male |
| 35 | Kyrgyzstan | CA | 35.1 | 41.67 | 1.19 | 1.09 | Male |
| 35 | Mongolia | CA | 33.99 | 42.38 | 1.25 | 1.15 | Male |
| 35 | Pakistan | CA | 36.7 | 40.2 | 1.1 | 1.01 | None |
| 35 | Tajikistan | CA | 37.63 | 41.24 | 1.1 | 1.01 | None |
| 35 | Turkmenistan | CA | 35.88 | 41.03 | 1.14 | 1.05 | Male |
| 35 | Uzbekistan | CA | 36.71 | 42.52 | 1.16 | 1.07 | Male |
| 35 | China | CIU | 41.97 | 47.09 | 1.12 | 1.03 | Male |
| 35 | India | CIU | 38.32 | 41.21 | 1.08 | 0.99 | None |
| 35 | Argentina | LAC | 41.46 | 46.07 | 1.11 | 1.02 | Male |
| 35 | Bahamas | LAC | 38.71 | 43.34 | 1.12 | 1.03 | Male |
| 35 | Belize | LAC | 37.29 | 42.27 | 1.13 | 1.04 | Male |
| 35 | Bolivia (Plurinational State Of) | LAC | 36.98 | 40.48 | 1.09 | 1.01 | None |
| 35 | Brazil | LAC | 41.14 | 45.87 | 1.11 | 1.03 | Male |
| 35 | Chile | LAC | 44.75 | 48.59 | 1.09 | 1 | None |
| 35 | Colombia | LAC | 42.54 | 46.59 | 1.1 | 1.01 | None |
| 35 | Costa Rica | LAC | 44.93 | 49.26 | 1.1 | 1.01 | None |
| 35 | Cuba | LAC | 41.48 | 45.95 | 1.11 | 1.02 | Male |
| 35 | Dominican Republic | LAC | 39.69 | 44.86 | 1.13 | 1.04 | Male |
| 35 | Ecuador | LAC | 42.96 | 46.92 | 1.09 | 1.01 | None |
| 35 | El Salvador | LAC | 36.02 | 42.96 | 1.19 | 1.1 | Male |
| 35 | Guatemala | LAC | 38.72 | 41.88 | 1.08 | 1 | None |
| 35 | Guyana | LAC | 34.79 | 41.13 | 1.18 | 1.09 | Male |
| 35 | Haiti | LAC | 34.49 | 38.56 | 1.12 | 1.03 | Male |
| 35 | Honduras | LAC | 37.67 | 42.06 | 1.12 | 1.03 | Male |
| 35 | Jamaica | LAC | 37.25 | 41.2 | 1.11 | 1.02 | Male |
| 35 | Mexico | LAC | 40.3 | 44.34 | 1.1 | 1.01 | Male |
| 35 | Nicaragua | LAC | 39.1 | 43.47 | 1.11 | 1.02 | Male |
| 35 | Panama | LAC | 43.93 | 48.78 | 1.11 | 1.02 | Male |
| 35 | Paraguay | LAC | 39.16 | 44.04 | 1.12 | 1.04 | Male |
| 35 | Peru | LAC | 42.3 | 45.78 | 1.08 | 1 | None |
| 35 | Suriname | LAC | 36.88 | 42.78 | 1.16 | 1.07 | Male |
| 35 | Trinidad And Tobago | LAC | 38.67 | 43.21 | 1.12 | 1.03 | Male |
| 35 | Uruguay | LAC | 40.85 | 47.59 | 1.17 | 1.07 | Male |
| 35 | Venezuela (Bolivarian Republic Of) | LAC | 39.45 | 44.29 | 1.12 | 1.03 | Male |
| 35 | Algeria | MENA | 42.32 | 44.54 | 1.05 | 0.97 | Female |
| 35 | Bahrain | MENA | 45.96 | 47.58 | 1.04 | 0.95 | Female |
| 35 | Egypt | MENA | 36.86 | 40.61 | 1.1 | 1.01 | Male |
| 35 | Iran (Islamic Republic Of) | MENA | 42.75 | 45.74 | 1.07 | 0.98 | Female |
| 35 | Iraq | MENA | 38.11 | 40.96 | 1.07 | 0.99 | Female |
| 35 | Israel | MENA | 46.93 | 50.27 | 1.07 | 0.99 | Female |
| 35 | Jordan | MENA | 41.82 | 45.96 | 1.1 | 1.01 | Male |
| 35 | Kuwait | MENA | 46.01 | 47.72 | 1.04 | 0.95 | Female |
| 35 | Lebanon | MENA | 43.05 | 46.73 | 1.09 | 1 | None |
| 35 | Libya | MENA | 38.2 | 43.13 | 1.13 | 1.04 | Male |
| 35 | Morocco | MENA | 39.86 | 43.53 | 1.09 | 1.01 | None |
| 35 | Oman | MENA | 45.25 | 48.22 | 1.07 | 0.98 | Female |
| 35 | Qatar | MENA | 48.34 | 49.85 | 1.03 | 0.95 | Female |
| 35 | Saudi Arabia | MENA | 43.9 | 46.5 | 1.06 | 0.97 | Female |
| 35 | Syrian Arab Republic | MENA | 37.73 | 42.13 | 1.12 | 1.03 | Male |
| 35 | Tunisia | MENA | 40.5 | 44.89 | 1.11 | 1.02 | Male |
| 35 | Türkiye | MENA | 41.09 | 47.04 | 1.14 | 1.05 | Male |
| 35 | United Arab Emirates | MENA | 47.73 | 49.77 | 1.04 | 0.96 | Female |
| 35 | Yemen | MENA | 35.66 | 39.8 | 1.12 | 1.03 | Male |
| 35 | Austria | NA | 45.52 | 49.78 | 1.09 | 1.01 | None |
| 35 | Belgium | NA | 45.58 | 49.63 | 1.09 | 1 | None |
| 35 | Canada | NA | 46.56 | 50.24 | 1.08 | 0.99 | None |
| 35 | Cyprus | NA | 44.93 | 49.06 | 1.09 | 1.01 | None |
| 35 | Denmark | NA | 45.25 | 48.95 | 1.08 | 1 | None |
| 35 | Finland | NA | 45.3 | 50.17 | 1.11 | 1.02 | Male |
| 35 | France | NA | 45.86 | 51.23 | 1.12 | 1.03 | Male |
| 35 | Germany | NA | 44.69 | 49.12 | 1.1 | 1.01 | Male |
| 35 | Greece | NA | 44.51 | 49.39 | 1.11 | 1.02 | Male |
| 35 | Iceland | NA | 47.25 | 49.91 | 1.06 | 0.97 | Female |
| 35 | Ireland | NA | 46.34 | 49.97 | 1.08 | 0.99 | None |
| 35 | Italy | NA | 46.9 | 50.86 | 1.08 | 1 | None |
| 35 | Luxembourg | NA | 46.11 | 50.08 | 1.09 | 1 | None |
| 35 | Malta | NA | 46.74 | 51.18 | 1.1 | 1.01 | None |
| 35 | Netherlands | NA | 46.3 | 49.19 | 1.06 | 0.98 | Female |
| 35 | Norway | NA | 47.09 | 50.22 | 1.07 | 0.98 | Female |
| 35 | Portugal | NA | 44.88 | 50.37 | 1.12 | 1.03 | Male |
| 35 | Spain | NA | 46.46 | 51.63 | 1.11 | 1.02 | Male |
| 35 | Sweden | NA | 47.23 | 50.28 | 1.06 | 0.98 | Female |
| 35 | Switzerland | NA | 47.73 | 51.13 | 1.07 | 0.99 | Female |
| 35 | United Kingdom | NA | 45.63 | 48.99 | 1.07 | 0.99 | Female |
| 35 | Angola | SSA | 34.29 | 37.92 | 1.11 | 1.02 | Male |
| 35 | Benin | SSA | 35.25 | 36.69 | 1.04 | 0.96 | Female |
| 35 | Botswana | SSA | 35.47 | 39.47 | 1.11 | 1.02 | Male |
| 35 | Burkina Faso | SSA | 33.91 | 36.78 | 1.08 | 1 | None |
| 35 | Burundi | SSA | 34.96 | 37.3 | 1.07 | 0.98 | Female |
| 35 | Cabo Verde | SSA | 39.38 | 45.21 | 1.15 | 1.06 | Male |
| 35 | Cameroon | SSA | 33.85 | 36.86 | 1.09 | 1 | None |
| 35 | Central African Republic | SSA | 16.92 | 25.17 | 1.49 | 1.37 | Male |
| 35 | Chad | SSA | 31.67 | 33.79 | 1.07 | 0.98 | Female |
| 35 | Comoros | SSA | 35.21 | 38.39 | 1.09 | 1 | None |
| 35 | Congo | SSA | 33.53 | 36.07 | 1.08 | 0.99 | None |
| 35 | Côte D'ivoire | SSA | 32.67 | 35.16 | 1.08 | 0.99 | None |
| 35 | Democratic Republic Of The Congo | SSA | 34.93 | 37.19 | 1.06 | 0.98 | Female |
| 35 | Djibouti | SSA | 36.04 | 39.06 | 1.08 | 1 | None |
| 35 | Equatorial Guinea | SSA | 34.85 | 37.25 | 1.07 | 0.98 | Female |
| 35 | Eritrea | SSA | 37.37 | 39.9 | 1.07 | 0.98 | Female |
| 35 | Eswatini | SSA | 29.88 | 34.51 | 1.16 | 1.06 | Male |
| 35 | Ethiopia | SSA | 36.39 | 40.06 | 1.1 | 1.01 | Male |
| 35 | Gabon | SSA | 35.85 | 39.46 | 1.1 | 1.01 | Male |
| 35 | Gambia | SSA | 36.36 | 38.19 | 1.05 | 0.97 | Female |
| 35 | Ghana | SSA | 35.86 | 38.45 | 1.07 | 0.99 | Female |
| 35 | Guinea | SSA | 35.58 | 36.9 | 1.04 | 0.95 | Female |
| 35 | Guinea-Bissau | SSA | 34.24 | 37.47 | 1.09 | 1.01 | None |
| 35 | Kenya | SSA | 32.19 | 35.93 | 1.12 | 1.03 | Male |
| 35 | Lesotho | SSA | 27.27 | 31.5 | 1.16 | 1.06 | Male |
| 35 | Liberia | SSA | 33.94 | 35.31 | 1.04 | 0.96 | Female |
| 35 | Madagascar | SSA | 36.39 | 38.33 | 1.05 | 0.97 | Female |
| 35 | Malawi | SSA | 32.95 | 38.57 | 1.17 | 1.08 | Male |
| 35 | Mali | SSA | 34.37 | 36.44 | 1.06 | 0.98 | Female |
| 35 | Mauritania | SSA | 36.26 | 39.15 | 1.08 | 0.99 | None |
| 35 | Mauritius | SSA | 39.02 | 44.39 | 1.14 | 1.05 | Male |
| 35 | Mozambique | SSA | 32.08 | 36.13 | 1.13 | 1.04 | Male |
| 35 | Namibia | SSA | 31.03 | 37.69 | 1.21 | 1.12 | Male |
| 35 | Niger | SSA | 35.74 | 37.65 | 1.05 | 0.97 | Female |
| 35 | Nigeria | SSA | 32.58 | 33.1 | 1.02 | 0.94 | Female |
| 35 | Rwanda | SSA | 35.01 | 38.55 | 1.1 | 1.01 | Male |
| 35 | Senegal | SSA | 36.64 | 39.58 | 1.08 | 0.99 | None |
| 35 | Sierra Leone | SSA | 34.82 | 36.35 | 1.04 | 0.96 | Female |
| 35 | Somalia | SSA | 34.35 | 36.79 | 1.07 | 0.99 | Female |
| 35 | South Africa | SSA | 33.75 | 39.23 | 1.16 | 1.07 | Male |
| 35 | South Sudan | SSA | 33.8 | 36.82 | 1.09 | 1 | None |
| 35 | Sudan | SSA | 36.84 | 40.18 | 1.09 | 1 | None |
| 35 | Togo | SSA | 34.62 | 35.5 | 1.03 | 0.94 | Female |
| 35 | Uganda | SSA | 34.7 | 39.56 | 1.14 | 1.05 | Male |
| 35 | United Republic Of Tanzania | SSA | 34.23 | 39.24 | 1.15 | 1.06 | Male |
| 35 | Zambia | SSA | 33.16 | 36.97 | 1.11 | 1.03 | Male |
| 35 | Zimbabwe | SSA | 31.1 | 34.8 | 1.12 | 1.03 | Male |
| 35 | United States Of America | CIU | 43.5 | 47.67 | 1.1 | 1.01 | None |
| 35 | Australia | WPSA | 47.31 | 50.93 | 1.08 | 0.99 | None |
| 35 | Bangladesh | WPSA | 40.06 | 43.2 | 1.08 | 0.99 | None |
| 35 | Bhutan | WPSA | 38.82 | 41.71 | 1.07 | 0.99 | Female |
| 35 | Brunei Darussalam | WPSA | 40.02 | 43.87 | 1.1 | 1.01 | None |
| 35 | Cambodia | WPSA | 38.18 | 41.37 | 1.08 | 1 | None |
| 35 | Dem. People's Republic Of Korea | WPSA | 38.95 | 43.5 | 1.12 | 1.03 | Male |
| 35 | Fiji | WPSA | 33.16 | 36.73 | 1.11 | 1.02 | Male |
| 35 | Indonesia | WPSA | 36.95 | 40.49 | 1.1 | 1.01 | None |
| 35 | Japan | WPSA | 47.13 | 52.98 | 1.12 | 1.03 | Male |
| 35 | Lao People's Democratic Republic | WPSA | 36.54 | 39.7 | 1.09 | 1 | None |
| 35 | Malaysia | WPSA | 40.45 | 44.78 | 1.11 | 1.02 | Male |
| 35 | Maldives | WPSA | 44.55 | 47.44 | 1.06 | 0.98 | Female |
| 35 | Myanmar | WPSA | 35.05 | 39.23 | 1.12 | 1.03 | Male |
| 35 | Nepal | WPSA | 37.21 | 39.52 | 1.06 | 0.98 | Female |
| 35 | New Zealand | WPSA | 46.43 | 49.58 | 1.07 | 0.98 | Female |
| 35 | Papua New Guinea | WPSA | 34.03 | 38.54 | 1.13 | 1.04 | Male |
| 35 | Philippines | WPSA | 35.7 | 40.78 | 1.14 | 1.05 | Male |
| 35 | Republic Of Korea | WPSA | 45.87 | 52.18 | 1.14 | 1.05 | Male |
| 35 | Singapore | WPSA | 47.08 | 51.23 | 1.09 | 1 | None |
| 35 | Solomon Islands | WPSA | 37.24 | 39.43 | 1.06 | 0.97 | Female |
| 35 | Sri Lanka | WPSA | 39.85 | 45.91 | 1.15 | 1.06 | Male |
| 35 | Taiwan | WPSA | 43.52 | 49.88 | 1.15 | 1.05 | Male |
| 35 | Thailand | WPSA | 40.84 | 48.39 | 1.18 | 1.09 | Male |
| 35 | Timor-Leste | WPSA | 36.29 | 38.86 | 1.07 | 0.99 | Female |
| 35 | Vanuatu | WPSA | 37.06 | 41.06 | 1.11 | 1.02 | Male |
| 35 | Viet Nam | WPSA | 39.16 | 46.46 | 1.19 | 1.09 | Male |
| 50 | American Samoa |  | 24.77 | 28.84 | 1.16 | 1.04 | Male |
| 50 | Andorra |  | 33.42 | 37.28 | 1.12 | 0.99 | None |
| 50 | Anguilla |  | 27.71 | 33.46 | 1.21 | 1.08 | Male |
| 50 | Antigua And Barbuda |  | 27.67 | 32.65 | 1.18 | 1.05 | Male |
| 50 | Aruba |  | 26.74 | 31.17 | 1.17 | 1.04 | Male |
| 50 | Barbados |  | 27.88 | 31.33 | 1.12 | 1 | None |
| 50 | Bermuda |  | 29.71 | 36.17 | 1.22 | 1.08 | Male |
| 50 | Bonaire, Sint Eustatius And Saba |  | 27.41 | 32.57 | 1.19 | 1.06 | Male |
| 50 | British Virgin Islands |  | 27.13 | 31.42 | 1.16 | 1.03 | Male |
| 50 | Cayman Islands |  | 29.65 | 34.04 | 1.15 | 1.02 | Male |
| 50 | China, Hong Kong Sar |  | 33.79 | 39.17 | 1.16 | 1.03 | Male |
| 50 | China, Macao Sar |  | 31.93 | 36.66 | 1.15 | 1.02 | Male |
| 50 | Cook Islands |  | 25.31 | 30.77 | 1.22 | 1.08 | Male |
| 50 | Curaçao |  | 25.51 | 32.48 | 1.27 | 1.13 | Male |
| 50 | Dominica |  | 24.12 | 28.37 | 1.18 | 1.05 | Male |
| 50 | Falkland Islands (Malvinas) |  | 28.81 | 32.73 | 1.14 | 1.01 | None |
| 50 | Faroe Islands |  | 29.7 | 33.31 | 1.12 | 1 | None |
| 50 | French Guiana |  | 27.69 | 32.11 | 1.16 | 1.03 | Male |
| 50 | French Polynesia |  | 32.27 | 36.98 | 1.15 | 1.02 | Male |
| 50 | Gibraltar |  | 33.39 | 37.68 | 1.13 | 1.01 | None |
| 50 | Greenland |  | 25.24 | 27.53 | 1.09 | 0.97 | Female |
| 50 | Grenada |  | 26.18 | 31.71 | 1.21 | 1.08 | Male |
| 50 | Guadeloupe |  | 30.62 | 36.79 | 1.2 | 1.07 | Male |
| 50 | Guam |  | 28.53 | 34.15 | 1.2 | 1.07 | Male |
| 50 | Guernsey |  | 32.5 | 36.69 | 1.13 | 1.01 | None |
| 50 | Holy See |  | 32.8 | 36.85 | 1.12 | 1 | None |
| 50 | Isle Of Man |  | 32.03 | 35.35 | 1.1 | 0.98 | Female |
| 50 | Jersey |  | 29.59 | 34.04 | 1.15 | 1.02 | Male |
| 50 | Kiribati |  | 23.15 | 25.48 | 1.1 | 0.98 | Female |
| 50 | Kosovo (Under Unsc Res. 1244) |  | 27.38 | 31.1 | 1.14 | 1.01 | None |
| 50 | Liechtenstein |  | 32.8 | 36.52 | 1.11 | 0.99 | None |
| 50 | Marshall Islands |  | 21.7 | 25.35 | 1.17 | 1.04 | Male |
| 50 | Martinique |  | 31.33 | 36.64 | 1.17 | 1.04 | Male |
| 50 | Mayotte |  | 27.15 | 30.43 | 1.12 | 1 | None |
| 50 | Micronesia (Fed. States Of) |  | 20.94 | 25.81 | 1.23 | 1.1 | Male |
| 50 | Monaco |  | 35.2 | 39.22 | 1.11 | 0.99 | None |
| 50 | Montserrat |  | 26.12 | 30.31 | 1.16 | 1.03 | Male |
| 50 | Nauru |  | 19.08 | 23.76 | 1.24 | 1.11 | Male |
| 50 | New Caledonia |  | 30.37 | 32.82 | 1.08 | 0.96 | Female |
| 50 | Niue |  | 22.89 | 26.99 | 1.18 | 1.05 | Male |
| 50 | Northern Mariana Islands |  | 28.46 | 31.64 | 1.11 | 0.99 | None |
| 50 | Palau |  | 22.64 | 26.96 | 1.19 | 1.06 | Male |
| 50 | Puerto Rico |  | 30.69 | 36.48 | 1.19 | 1.06 | Male |
| 50 | Réunion |  | 31.71 | 36.85 | 1.16 | 1.04 | Male |
| 50 | Saint Barthélemy |  | 31.49 | 38.55 | 1.22 | 1.09 | Male |
| 50 | Saint Helena |  | 26.9 | 32.6 | 1.21 | 1.08 | Male |
| 50 | Saint Kitts And Nevis |  | 23.37 | 28.8 | 1.23 | 1.1 | Male |
| 50 | Saint Lucia |  | 23.79 | 29.14 | 1.22 | 1.09 | Male |
| 50 | Saint Martin (French Part) |  | 28.68 | 35.36 | 1.23 | 1.1 | Male |
| 50 | Saint Pierre And Miquelon |  | 26.04 | 32.67 | 1.25 | 1.12 | Male |
| 50 | Saint Vincent And The Grenadines |  | 23.81 | 28.33 | 1.19 | 1.06 | Male |
| 50 | Samoa |  | 23.9 | 27.35 | 1.14 | 1.02 | Male |
| 50 | San Marino |  | 34.51 | 37.47 | 1.09 | 0.97 | Female |
| 50 | Sao Tome And Principe |  | 21.62 | 26.98 | 1.25 | 1.11 | Male |
| 50 | Seychelles |  | 24.04 | 29.18 | 1.21 | 1.08 | Male |
| 50 | Sint Maarten (Dutch Part) |  | 26.43 | 31.09 | 1.18 | 1.05 | Male |
| 50 | State Of Palestine |  | 26.77 | 30.72 | 1.15 | 1.02 | Male |
| 50 | Tokelau |  | 27.32 | 31.09 | 1.14 | 1.01 | None |
| 50 | Tonga |  | 23.81 | 29.2 | 1.23 | 1.09 | Male |
| 50 | Turks And Caicos Islands |  | 28.09 | 31.9 | 1.14 | 1.01 | None |
| 50 | Tuvalu |  | 20.76 | 25.96 | 1.25 | 1.11 | Male |
| 50 | United States Virgin Islands |  | 24.14 | 32.54 | 1.35 | 1.2 | Male |
| 50 | Wallis And Futuna Islands |  | 28.92 | 31.83 | 1.1 | 0.98 | Female |
| 50 | Western Sahara |  | 24.53 | 27.04 | 1.1 | 0.98 | Female |
| 50 | Albania | CEE | 30.45 | 32.92 | 1.08 | 0.96 | Female |
| 50 | Armenia | CEE | 24.61 | 30.72 | 1.25 | 1.11 | Male |
| 50 | Belarus | CEE | 22.97 | 30.71 | 1.34 | 1.19 | Male |
| 50 | Bosnia And Herzegovina | CEE | 26.15 | 31.87 | 1.22 | 1.09 | Male |
| 50 | Bulgaria | CEE | 24.75 | 30.7 | 1.24 | 1.1 | Male |
| 50 | Croatia | CEE | 27.08 | 32.61 | 1.2 | 1.07 | Male |
| 50 | Czechia | CEE | 28.32 | 33.28 | 1.18 | 1.05 | Male |
| 50 | Estonia | CEE | 26.93 | 34.12 | 1.27 | 1.13 | Male |
| 50 | Georgia | CEE | 23.43 | 30.89 | 1.32 | 1.17 | Male |
| 50 | Hungary | CEE | 25.27 | 31.06 | 1.23 | 1.09 | Male |
| 50 | Latvia | CEE | 24.7 | 31.97 | 1.29 | 1.15 | Male |
| 50 | Lithuania | CEE | 25.26 | 32.79 | 1.3 | 1.16 | Male |
| 50 | Montenegro | CEE | 25.93 | 31.5 | 1.21 | 1.08 | Male |
| 50 | North Macedonia | CEE | 26.61 | 30.47 | 1.15 | 1.02 | Male |
| 50 | Poland | CEE | 27.01 | 33.11 | 1.23 | 1.09 | Male |
| 50 | Republic Of Moldova | CEE | 21.16 | 27.68 | 1.31 | 1.17 | Male |
| 50 | Romania | CEE | 24.87 | 30.83 | 1.24 | 1.1 | Male |
| 50 | Russian Federation | CEE | 23.18 | 30.91 | 1.33 | 1.19 | Male |
| 50 | Serbia | CEE | 25.24 | 30.88 | 1.22 | 1.09 | Male |
| 50 | Slovakia | CEE | 26.9 | 32.57 | 1.21 | 1.08 | Male |
| 50 | Slovenia | CEE | 29.94 | 34.66 | 1.16 | 1.03 | Male |
| 50 | Ukraine | CEE | 23.83 | 31.2 | 1.31 | 1.17 | Male |
| 50 | Afghanistan | CA | 22.35 | 25.13 | 1.12 | 1 | None |
| 50 | Azerbaijan | CA | 24.66 | 29.17 | 1.18 | 1.05 | Male |
| 50 | Kazakhstan | CA | 24.03 | 30.37 | 1.26 | 1.13 | Male |
| 50 | Kyrgyzstan | CA | 22.61 | 27.84 | 1.23 | 1.1 | Male |
| 50 | Mongolia | CA | 22 | 28.7 | 1.3 | 1.16 | Male |
| 50 | Pakistan | CA | 23.62 | 26.65 | 1.13 | 1 | None |
| 50 | Tajikistan | CA | 24.23 | 27.41 | 1.13 | 1.01 | None |
| 50 | Turkmenistan | CA | 23.02 | 27.15 | 1.18 | 1.05 | Male |
| 50 | Uzbekistan | CA | 23.69 | 28.77 | 1.21 | 1.08 | Male |
| 50 | China | CIU | 28.07 | 32.74 | 1.17 | 1.04 | Male |
| 50 | India | CIU | 25.31 | 27.47 | 1.09 | 0.97 | Female |
| 50 | Argentina | LAC | 27.69 | 32.07 | 1.16 | 1.03 | Male |
| 50 | Bahamas | LAC | 26.48 | 30.68 | 1.16 | 1.03 | Male |
| 50 | Belize | LAC | 24.35 | 28.48 | 1.17 | 1.04 | Male |
| 50 | Bolivia (Plurinational State Of) | LAC | 24.52 | 27.31 | 1.11 | 0.99 | None |
| 50 | Brazil | LAC | 27.97 | 31.9 | 1.14 | 1.02 | Male |
| 50 | Chile | LAC | 30.95 | 34.31 | 1.11 | 0.99 | None |
| 50 | Colombia | LAC | 29.04 | 32.5 | 1.12 | 1 | None |
| 50 | Costa Rica | LAC | 31.35 | 35.05 | 1.12 | 1 | None |
| 50 | Cuba | LAC | 27.79 | 31.77 | 1.14 | 1.02 | Male |
| 50 | Dominican Republic | LAC | 26.66 | 31.24 | 1.17 | 1.04 | Male |
| 50 | Ecuador | LAC | 29.65 | 32.94 | 1.11 | 0.99 | None |
| 50 | El Salvador | LAC | 24.74 | 29.24 | 1.18 | 1.05 | Male |
| 50 | Guatemala | LAC | 26.21 | 28.33 | 1.08 | 0.96 | Female |
| 50 | Guyana | LAC | 22.72 | 27.83 | 1.23 | 1.09 | Male |
| 50 | Haiti | LAC | 22.23 | 25.47 | 1.15 | 1.02 | Male |
| 50 | Honduras | LAC | 24.34 | 28.39 | 1.17 | 1.04 | Male |
| 50 | Jamaica | LAC | 24.19 | 27.52 | 1.14 | 1.01 | None |
| 50 | Mexico | LAC | 27.77 | 30.45 | 1.1 | 0.98 | Female |
| 50 | Nicaragua | LAC | 26.21 | 29.53 | 1.13 | 1 | None |
| 50 | Panama | LAC | 30.3 | 34.69 | 1.14 | 1.02 | Male |
| 50 | Paraguay | LAC | 25.9 | 30.17 | 1.16 | 1.04 | Male |
| 50 | Peru | LAC | 28.98 | 32.03 | 1.11 | 0.98 | Female |
| 50 | Suriname | LAC | 23.96 | 29.45 | 1.23 | 1.09 | Male |
| 50 | Trinidad And Tobago | LAC | 25.67 | 29.6 | 1.15 | 1.03 | Male |
| 50 | Uruguay | LAC | 27.36 | 33.6 | 1.23 | 1.09 | Male |
| 50 | Venezuela (Bolivarian Republic Of) | LAC | 26.56 | 30.59 | 1.15 | 1.03 | Male |
| 50 | Algeria | MENA | 28.19 | 30.39 | 1.08 | 0.96 | Female |
| 50 | Bahrain | MENA | 31.58 | 33.13 | 1.05 | 0.93 | Female |
| 50 | Egypt | MENA | 23.08 | 26.45 | 1.15 | 1.02 | Male |
| 50 | Iran (Islamic Republic Of) | MENA | 28.96 | 31.41 | 1.08 | 0.97 | Female |
| 50 | Iraq | MENA | 24.65 | 27.25 | 1.11 | 0.98 | Female |
| 50 | Israel | MENA | 32.65 | 35.71 | 1.09 | 0.97 | Female |
| 50 | Jordan | MENA | 27.62 | 31.67 | 1.15 | 1.02 | Male |
| 50 | Kuwait | MENA | 31.48 | 32.99 | 1.05 | 0.93 | Female |
| 50 | Lebanon | MENA | 28.74 | 32.39 | 1.13 | 1 | None |
| 50 | Libya | MENA | 25.08 | 29.38 | 1.17 | 1.04 | Male |
| 50 | Morocco | MENA | 25.94 | 29.46 | 1.14 | 1.01 | None |
| 50 | Oman | MENA | 30.87 | 33.68 | 1.09 | 0.97 | Female |
| 50 | Qatar | MENA | 33.72 | 35.11 | 1.04 | 0.93 | Female |
| 50 | Saudi Arabia | MENA | 29.72 | 32.12 | 1.08 | 0.96 | Female |
| 50 | Syrian Arab Republic | MENA | 24.71 | 28.31 | 1.15 | 1.02 | Male |
| 50 | Tunisia | MENA | 26.67 | 30.6 | 1.15 | 1.02 | Male |
| 50 | Türkiye | MENA | 27.06 | 32.6 | 1.2 | 1.07 | Male |
| 50 | United Arab Emirates | MENA | 33.22 | 35.02 | 1.05 | 0.94 | Female |
| 50 | Yemen | MENA | 23.28 | 26.39 | 1.13 | 1.01 | None |
| 50 | Austria | NA | 31.32 | 35.27 | 1.13 | 1 | None |
| 50 | Belgium | NA | 31.47 | 35.19 | 1.12 | 1 | None |
| 50 | Canada | NA | 32.53 | 35.86 | 1.1 | 0.98 | Female |
| 50 | Cyprus | NA | 30.55 | 34.41 | 1.13 | 1 | None |
| 50 | Denmark | NA | 30.98 | 34.4 | 1.11 | 0.99 | None |
| 50 | Finland | NA | 31.28 | 35.7 | 1.14 | 1.02 | Male |
| 50 | France | NA | 31.89 | 36.84 | 1.15 | 1.03 | Male |
| 50 | Germany | NA | 30.54 | 34.65 | 1.13 | 1.01 | None |
| 50 | Greece | NA | 30.35 | 34.81 | 1.15 | 1.02 | Male |
| 50 | Iceland | NA | 33.02 | 35.46 | 1.07 | 0.96 | Female |
| 50 | Ireland | NA | 32.13 | 35.48 | 1.1 | 0.98 | Female |
| 50 | Italy | NA | 32.56 | 36.3 | 1.12 | 0.99 | None |
| 50 | Luxembourg | NA | 31.71 | 35.58 | 1.12 | 1 | None |
| 50 | Malta | NA | 32.34 | 36.72 | 1.14 | 1.01 | None |
| 50 | Netherlands | NA | 31.98 | 34.69 | 1.08 | 0.97 | Female |
| 50 | Norway | NA | 32.77 | 35.63 | 1.09 | 0.97 | Female |
| 50 | Portugal | NA | 30.86 | 35.94 | 1.16 | 1.04 | Male |
| 50 | Spain | NA | 32.12 | 37.05 | 1.15 | 1.03 | Male |
| 50 | Sweden | NA | 32.85 | 35.73 | 1.09 | 0.97 | Female |
| 50 | Switzerland | NA | 33.29 | 36.52 | 1.1 | 0.98 | Female |
| 50 | United Kingdom | NA | 31.69 | 34.7 | 1.09 | 0.98 | Female |
| 50 | Angola | SSA | 22.33 | 25.19 | 1.13 | 1 | None |
| 50 | Benin | SSA | 23.37 | 24.63 | 1.05 | 0.94 | Female |
| 50 | Botswana | SSA | 23.26 | 26.68 | 1.15 | 1.02 | Male |
| 50 | Burkina Faso | SSA | 21.91 | 24.18 | 1.1 | 0.98 | Female |
| 50 | Burundi | SSA | 23.19 | 25.05 | 1.08 | 0.96 | Female |
| 50 | Cabo Verde | SSA | 26.41 | 31.15 | 1.18 | 1.05 | Male |
| 50 | Cameroon | SSA | 22.14 | 24.48 | 1.11 | 0.98 | Female |
| 50 | Central African Republic | SSA | 12.07 | 16.19 | 1.34 | 1.19 | Male |
| 50 | Chad | SSA | 20.98 | 22.69 | 1.08 | 0.96 | Female |
| 50 | Comoros | SSA | 22.39 | 25.33 | 1.13 | 1.01 | None |
| 50 | Congo | SSA | 21.83 | 24.07 | 1.1 | 0.98 | Female |
| 50 | Côte D'ivoire | SSA | 21.37 | 23.61 | 1.1 | 0.98 | Female |
| 50 | Democratic Republic Of The Congo | SSA | 23.18 | 24.98 | 1.08 | 0.96 | Female |
| 50 | Djibouti | SSA | 23.92 | 26.28 | 1.1 | 0.98 | Female |
| 50 | Equatorial Guinea | SSA | 22.71 | 24.72 | 1.09 | 0.97 | Female |
| 50 | Eritrea | SSA | 24.85 | 26.9 | 1.08 | 0.96 | Female |
| 50 | Eswatini | SSA | 20.28 | 23.64 | 1.17 | 1.04 | Male |
| 50 | Ethiopia | SSA | 24.17 | 27.01 | 1.12 | 1 | None |
| 50 | Gabon | SSA | 23.53 | 26.44 | 1.12 | 1 | None |
| 50 | Gambia | SSA | 24.15 | 25.67 | 1.06 | 0.95 | Female |
| 50 | Ghana | SSA | 23.8 | 25.85 | 1.09 | 0.97 | Female |
| 50 | Guinea | SSA | 23.6 | 24.77 | 1.05 | 0.93 | Female |
| 50 | Guinea-Bissau | SSA | 22.45 | 25 | 1.11 | 0.99 | None |
| 50 | Kenya | SSA | 21.44 | 24.58 | 1.15 | 1.02 | Male |
| 50 | Lesotho | SSA | 18.35 | 21.78 | 1.19 | 1.06 | Male |
| 50 | Liberia | SSA | 21.98 | 23.1 | 1.05 | 0.94 | Female |
| 50 | Madagascar | SSA | 24.17 | 25.76 | 1.07 | 0.95 | Female |
| 50 | Malawi | SSA | 21.6 | 25.9 | 1.2 | 1.07 | Male |
| 50 | Mali | SSA | 22.23 | 23.93 | 1.08 | 0.96 | Female |
| 50 | Mauritania | SSA | 23.14 | 25.94 | 1.12 | 1 | None |
| 50 | Mauritius | SSA | 26.33 | 30.65 | 1.16 | 1.04 | Male |
| 50 | Mozambique | SSA | 20.8 | 23.64 | 1.14 | 1.01 | None |
| 50 | Namibia | SSA | 20.67 | 25.86 | 1.25 | 1.11 | Male |
| 50 | Niger | SSA | 23.13 | 24.8 | 1.07 | 0.95 | Female |
| 50 | Nigeria | SSA | 21.57 | 22.2 | 1.03 | 0.92 | Female |
| 50 | Rwanda | SSA | 22.87 | 25.66 | 1.12 | 1 | None |
| 50 | Senegal | SSA | 23.75 | 26.21 | 1.1 | 0.98 | Female |
| 50 | Sierra Leone | SSA | 22.24 | 23.85 | 1.07 | 0.96 | Female |
| 50 | Somalia | SSA | 22.87 | 24.7 | 1.08 | 0.96 | Female |
| 50 | South Africa | SSA | 22.64 | 27.5 | 1.21 | 1.08 | Male |
| 50 | South Sudan | SSA | 22.44 | 24.72 | 1.1 | 0.98 | Female |
| 50 | Sudan | SSA | 24.49 | 27.09 | 1.11 | 0.99 | None |
| 50 | Togo | SSA | 22.37 | 23.25 | 1.04 | 0.93 | Female |
| 50 | Uganda | SSA | 23.07 | 27.3 | 1.18 | 1.05 | Male |
| 50 | United Republic Of Tanzania | SSA | 22.42 | 26.71 | 1.19 | 1.06 | Male |
| 50 | Zambia | SSA | 21.61 | 24.42 | 1.13 | 1.01 | None |
| 50 | Zimbabwe | SSA | 20.66 | 23.79 | 1.15 | 1.03 | Male |
| 50 | United States Of America | CIU | 30.12 | 33.7 | 1.12 | 1 | None |
| 50 | Australia | WPSA | 33.27 | 36.51 | 1.1 | 0.98 | Female |
| 50 | Bangladesh | WPSA | 26.45 | 29.41 | 1.11 | 0.99 | None |
| 50 | Bhutan | WPSA | 25.14 | 27.87 | 1.11 | 0.99 | None |
| 50 | Brunei Darussalam | WPSA | 26.35 | 29.99 | 1.14 | 1.01 | Male |
| 50 | Cambodia | WPSA | 25.44 | 27.95 | 1.1 | 0.98 | Female |
| 50 | Dem. People's Republic Of Korea | WPSA | 25.78 | 29.66 | 1.15 | 1.02 | Male |
| 50 | Fiji | WPSA | 20.2 | 23.48 | 1.16 | 1.04 | Male |
| 50 | Indonesia | WPSA | 23.74 | 27.12 | 1.14 | 1.02 | Male |
| 50 | Japan | WPSA | 32.84 | 38.47 | 1.17 | 1.04 | Male |
| 50 | Lao People's Democratic Republic | WPSA | 23.51 | 26.27 | 1.12 | 1 | None |
| 50 | Malaysia | WPSA | 27.15 | 30.75 | 1.13 | 1.01 | None |
| 50 | Maldives | WPSA | 30.06 | 32.84 | 1.09 | 0.97 | Female |
| 50 | Myanmar | WPSA | 22.57 | 25.93 | 1.15 | 1.02 | Male |
| 50 | Nepal | WPSA | 23.97 | 26.13 | 1.09 | 0.97 | Female |
| 50 | New Zealand | WPSA | 32.34 | 35.22 | 1.09 | 0.97 | Female |
| 50 | Papua New Guinea | WPSA | 21.6 | 25.5 | 1.18 | 1.05 | Male |
| 50 | Philippines | WPSA | 23.03 | 27.3 | 1.19 | 1.06 | Male |
| 50 | Republic Of Korea | WPSA | 31.42 | 37.46 | 1.19 | 1.06 | Male |
| 50 | Singapore | WPSA | 32.71 | 36.65 | 1.12 | 1 | None |
| 50 | Solomon Islands | WPSA | 23.99 | 26.06 | 1.09 | 0.97 | Female |
| 50 | Sri Lanka | WPSA | 26.3 | 31.59 | 1.2 | 1.07 | Male |
| 50 | Taiwan | WPSA | 30.07 | 35.56 | 1.18 | 1.05 | Male |
| 50 | Thailand | WPSA | 28.78 | 34.67 | 1.2 | 1.07 | Male |
| 50 | Timor-Leste | WPSA | 23.35 | 25.68 | 1.1 | 0.98 | Female |
| 50 | Vanuatu | WPSA | 23.88 | 27.59 | 1.16 | 1.03 | Male |
| 50 | Viet Nam | WPSA | 26.13 | 32.4 | 1.24 | 1.1 | Male |
| 70 | American Samoa |  | 11.21 | 12.91 | 1.15 | 0.97 | None |
| 70 | Andorra |  | 15.85 | 18.79 | 1.19 | 1 | None |
| 70 | Anguilla |  | 12.26 | 16.03 | 1.31 | 1.1 | Male |
| 70 | Antigua And Barbuda |  | 12.51 | 16.04 | 1.28 | 1.08 | Male |
| 70 | Aruba |  | 11.1 | 13.77 | 1.24 | 1.04 | Male |
| 70 | Barbados |  | 12.41 | 14.31 | 1.15 | 0.97 | None |
| 70 | Bermuda |  | 13.63 | 18.44 | 1.35 | 1.14 | Male |
| 70 | Bonaire, Sint Eustatius And Saba |  | 11.73 | 15.14 | 1.29 | 1.09 | Male |
| 70 | British Virgin Islands |  | 11.56 | 14.11 | 1.22 | 1.03 | Male |
| 70 | Cayman Islands |  | 14.12 | 17.38 | 1.23 | 1.04 | Male |
| 70 | China, Hong Kong Sar |  | 16.92 | 21.02 | 1.24 | 1.05 | Male |
| 70 | China, Macao Sar |  | 14.57 | 18.02 | 1.24 | 1.04 | Male |
| 70 | Cook Islands |  | 11.42 | 14.3 | 1.25 | 1.05 | Male |
| 70 | Curaçao |  | 10.67 | 15.06 | 1.41 | 1.19 | Male |
| 70 | Dominica |  | 10.06 | 12.1 | 1.2 | 1.01 | None |
| 70 | Falkland Islands (Malvinas) |  | 12.61 | 15.25 | 1.21 | 1.02 | None |
| 70 | Faroe Islands |  | 13.01 | 15.52 | 1.19 | 1 | None |
| 70 | French Guiana |  | 11.46 | 14.31 | 1.25 | 1.05 | Male |
| 70 | French Polynesia |  | 15 | 18.55 | 1.24 | 1.04 | Male |
| 70 | Gibraltar |  | 16.22 | 19.37 | 1.19 | 1.01 | None |
| 70 | Greenland |  | 10.95 | 12.05 | 1.1 | 0.93 | Female |
| 70 | Grenada |  | 12.14 | 16.3 | 1.34 | 1.13 | Male |
| 70 | Guadeloupe |  | 15.36 | 19.17 | 1.25 | 1.05 | Male |
| 70 | Guam |  | 14.95 | 18.58 | 1.24 | 1.05 | Male |
| 70 | Guernsey |  | 15.17 | 18.31 | 1.21 | 1.02 | None |
| 70 | Holy See |  | 15.39 | 18.44 | 1.2 | 1.01 | None |
| 70 | Isle Of Man |  | 15.23 | 17.54 | 1.15 | 0.97 | None |
| 70 | Jersey |  | 13.12 | 16.24 | 1.24 | 1.04 | Male |
| 70 | Kiribati |  | 9.68 | 10.72 | 1.11 | 0.93 | Female |
| 70 | Kosovo (Under Unsc Res. 1244) |  | 11.71 | 13.86 | 1.18 | 1 | None |
| 70 | Liechtenstein |  | 15.39 | 18.18 | 1.18 | 0.99 | None |
| 70 | Marshall Islands |  | 9.18 | 11.05 | 1.2 | 1.01 | None |
| 70 | Martinique |  | 15.46 | 18.58 | 1.2 | 1.01 | None |
| 70 | Mayotte |  | 11.78 | 13.84 | 1.17 | 0.99 | None |
| 70 | Micronesia (Fed. States Of) |  | 9.02 | 11.36 | 1.26 | 1.06 | Male |
| 70 | Monaco |  | 17.2 | 20.4 | 1.19 | 1 | None |
| 70 | Montserrat |  | 10.97 | 13.5 | 1.23 | 1.04 | Male |
| 70 | Nauru |  | 8.43 | 10.6 | 1.26 | 1.06 | Male |
| 70 | New Caledonia |  | 14.13 | 15.56 | 1.1 | 0.93 | Female |
| 70 | Niue |  | 9.65 | 11.71 | 1.21 | 1.02 | None |
| 70 | Northern Mariana Islands |  | 12.6 | 14.68 | 1.17 | 0.98 | None |
| 70 | Palau |  | 9.67 | 11.87 | 1.23 | 1.03 | Male |
| 70 | Puerto Rico |  | 15.49 | 18.82 | 1.21 | 1.02 | None |
| 70 | Réunion |  | 15.81 | 19.06 | 1.21 | 1.01 | None |
| 70 | Saint Barthélemy |  | 14.44 | 19.84 | 1.37 | 1.16 | Male |
| 70 | Saint Helena |  | 11.43 | 15.16 | 1.33 | 1.12 | Male |
| 70 | Saint Kitts And Nevis |  | 10.1 | 12.83 | 1.27 | 1.07 | Male |
| 70 | Saint Lucia |  | 10.23 | 12.91 | 1.26 | 1.06 | Male |
| 70 | Saint Martin (French Part) |  | 12.52 | 17.26 | 1.38 | 1.16 | Male |
| 70 | Saint Pierre And Miquelon |  | 11.3 | 15.07 | 1.33 | 1.12 | Male |
| 70 | Saint Vincent And The Grenadines |  | 10.57 | 12.73 | 1.2 | 1.01 | None |
| 70 | Samoa |  | 10.28 | 12.26 | 1.19 | 1 | None |
| 70 | San Marino |  | 16.6 | 19.17 | 1.16 | 0.97 | None |
| 70 | Sao Tome And Principe |  | 9.53 | 12.1 | 1.27 | 1.07 | Male |
| 70 | Seychelles |  | 10.48 | 13.08 | 1.25 | 1.05 | Male |
| 70 | Sint Maarten (Dutch Part) |  | 11.17 | 14.06 | 1.26 | 1.06 | Male |
| 70 | State Of Palestine |  | 11.37 | 13.8 | 1.21 | 1.02 | None |
| 70 | Tokelau |  | 12.19 | 14.92 | 1.22 | 1.03 | Male |
| 70 | Tonga |  | 10.49 | 13.34 | 1.27 | 1.07 | Male |
| 70 | Turks And Caicos Islands |  | 12.55 | 15.1 | 1.2 | 1.01 | None |
| 70 | Tuvalu |  | 9.07 | 11.55 | 1.27 | 1.07 | Male |
| 70 | United States Virgin Islands |  | 10.1 | 15.11 | 1.5 | 1.26 | Male |
| 70 | Wallis And Futuna Islands |  | 13.23 | 15.4 | 1.16 | 0.98 | None |
| 70 | Western Sahara |  | 10.25 | 11.45 | 1.12 | 0.94 | Female |
| 70 | Albania | CEE | 14.34 | 15.09 | 1.05 | 0.89 | Female |
| 70 | Armenia | CEE | 10.83 | 13.7 | 1.27 | 1.06 | Male |
| 70 | Belarus | CEE | 10.6 | 14.14 | 1.33 | 1.12 | Male |
| 70 | Bosnia And Herzegovina | CEE | 11.24 | 15.17 | 1.35 | 1.14 | Male |
| 70 | Bulgaria | CEE | 11.41 | 14.16 | 1.24 | 1.04 | Male |
| 70 | Croatia | CEE | 12.01 | 15.18 | 1.26 | 1.06 | Male |
| 70 | Czechia | CEE | 12.95 | 15.89 | 1.23 | 1.03 | Male |
| 70 | Estonia | CEE | 12.8 | 16.68 | 1.3 | 1.1 | Male |
| 70 | Georgia | CEE | 10.33 | 14.26 | 1.38 | 1.16 | Male |
| 70 | Hungary | CEE | 12.02 | 14.79 | 1.23 | 1.04 | Male |
| 70 | Latvia | CEE | 11.62 | 15.37 | 1.32 | 1.11 | Male |
| 70 | Lithuania | CEE | 12.12 | 15.96 | 1.32 | 1.11 | Male |
| 70 | Montenegro | CEE | 11.16 | 14.71 | 1.32 | 1.11 | Male |
| 70 | North Macedonia | CEE | 11.4 | 13.66 | 1.2 | 1.01 | None |
| 70 | Poland | CEE | 13.03 | 16.23 | 1.25 | 1.05 | Male |
| 70 | Republic Of Moldova | CEE | 9.28 | 12.37 | 1.33 | 1.12 | Male |
| 70 | Romania | CEE | 11.86 | 14.24 | 1.2 | 1.01 | None |
| 70 | Russian Federation | CEE | 11.41 | 14.79 | 1.3 | 1.09 | Male |
| 70 | Serbia | CEE | 10.99 | 14.46 | 1.32 | 1.11 | Male |
| 70 | Slovakia | CEE | 12.58 | 15.49 | 1.23 | 1.04 | Male |
| 70 | Slovenia | CEE | 13.93 | 16.93 | 1.22 | 1.02 | None |
| 70 | Ukraine | CEE | 11.35 | 14.7 | 1.3 | 1.09 | Male |
| 70 | Afghanistan | CA | 9.45 | 10.59 | 1.12 | 0.94 | Female |
| 70 | Azerbaijan | CA | 10.42 | 12.58 | 1.21 | 1.02 | None |
| 70 | Kazakhstan | CA | 10.73 | 13.9 | 1.3 | 1.09 | Male |
| 70 | Kyrgyzstan | CA | 9.52 | 11.69 | 1.23 | 1.03 | Male |
| 70 | Mongolia | CA | 9.82 | 12.95 | 1.32 | 1.11 | Male |
| 70 | Pakistan | CA | 9.86 | 11.24 | 1.14 | 0.96 | Female |
| 70 | Tajikistan | CA | 10.05 | 11.68 | 1.16 | 0.98 | None |
| 70 | Turkmenistan | CA | 9.61 | 11.42 | 1.19 | 1 | None |
| 70 | Uzbekistan | CA | 10.32 | 13.02 | 1.26 | 1.06 | Male |
| 70 | China | CIU | 12.52 | 15.34 | 1.23 | 1.03 | Male |
| 70 | India | CIU | 11.66 | 12.67 | 1.09 | 0.91 | Female |
| 70 | Argentina | LAC | 12.72 | 15.71 | 1.23 | 1.04 | Male |
| 70 | Bahamas | LAC | 12.54 | 15.73 | 1.25 | 1.06 | Male |
| 70 | Belize | LAC | 11.27 | 12.88 | 1.14 | 0.96 | Female |
| 70 | Bolivia (Plurinational State Of) | LAC | 11.05 | 12.39 | 1.12 | 0.94 | Female |
| 70 | Brazil | LAC | 13.1 | 15.21 | 1.16 | 0.98 | None |
| 70 | Chile | LAC | 15.09 | 17.22 | 1.14 | 0.96 | Female |
| 70 | Colombia | LAC | 12.82 | 15.35 | 1.2 | 1.01 | None |
| 70 | Costa Rica | LAC | 15.1 | 17.72 | 1.17 | 0.99 | None |
| 70 | Cuba | LAC | 13.21 | 15.53 | 1.18 | 0.99 | None |
| 70 | Dominican Republic | LAC | 12.04 | 15.17 | 1.26 | 1.06 | Male |
| 70 | Ecuador | LAC | 13.82 | 16.27 | 1.18 | 0.99 | None |
| 70 | El Salvador | LAC | 11.47 | 13.55 | 1.18 | 0.99 | None |
| 70 | Guatemala | LAC | 11.78 | 12.93 | 1.1 | 0.92 | Female |
| 70 | Guyana | LAC | 10.9 | 13.52 | 1.24 | 1.04 | Male |
| 70 | Haiti | LAC | 9.32 | 10.71 | 1.15 | 0.97 | Female |
| 70 | Honduras | LAC | 10.39 | 12.62 | 1.21 | 1.02 | None |
| 70 | Jamaica | LAC | 10.48 | 12.1 | 1.16 | 0.97 | None |
| 70 | Mexico | LAC | 13.53 | 14.38 | 1.06 | 0.89 | Female |
| 70 | Nicaragua | LAC | 11.54 | 13.66 | 1.18 | 1 | None |
| 70 | Panama | LAC | 14.28 | 17.58 | 1.23 | 1.04 | Male |
| 70 | Paraguay | LAC | 11.68 | 14.08 | 1.21 | 1.01 | None |
| 70 | Peru | LAC | 13.49 | 15.88 | 1.18 | 0.99 | None |
| 70 | Suriname | LAC | 10.81 | 14.34 | 1.33 | 1.12 | Male |
| 70 | Trinidad And Tobago | LAC | 11.07 | 13.36 | 1.21 | 1.02 | None |
| 70 | Uruguay | LAC | 12.46 | 17.13 | 1.37 | 1.16 | Male |
| 70 | Venezuela (Bolivarian Republic Of) | LAC | 11.89 | 14.23 | 1.2 | 1.01 | None |
| 70 | Algeria | MENA | 11.91 | 13.45 | 1.13 | 0.95 | Female |
| 70 | Bahrain | MENA | 14.69 | 16 | 1.09 | 0.92 | Female |
| 70 | Egypt | MENA | 9.29 | 10.85 | 1.17 | 0.98 | None |
| 70 | Iran (Islamic Republic Of) | MENA | 12.51 | 13.9 | 1.11 | 0.94 | Female |
| 70 | Iraq | MENA | 10.31 | 11.56 | 1.12 | 0.94 | Female |
| 70 | Israel | MENA | 15.89 | 17.78 | 1.12 | 0.94 | Female |
| 70 | Jordan | MENA | 11.84 | 14.47 | 1.22 | 1.03 | Male |
| 70 | Kuwait | MENA | 13.13 | 14.65 | 1.12 | 0.94 | Female |
| 70 | Lebanon | MENA | 12.56 | 15 | 1.19 | 1.01 | None |
| 70 | Libya | MENA | 10.97 | 13.39 | 1.22 | 1.03 | Male |
| 70 | Morocco | MENA | 10.9 | 12.94 | 1.19 | 1 | None |
| 70 | Oman | MENA | 13.82 | 15.9 | 1.15 | 0.97 | Female |
| 70 | Qatar | MENA | 15.47 | 17.16 | 1.11 | 0.93 | Female |
| 70 | Saudi Arabia | MENA | 13.21 | 15.14 | 1.15 | 0.97 | Female |
| 70 | Syrian Arab Republic | MENA | 10.43 | 12.26 | 1.18 | 0.99 | None |
| 70 | Tunisia | MENA | 11.22 | 13.41 | 1.19 | 1.01 | None |
| 70 | Türkiye | MENA | 11.86 | 15.15 | 1.28 | 1.07 | Male |
| 70 | United Arab Emirates | MENA | 15 | 16.37 | 1.09 | 0.92 | Female |
| 70 | Yemen | MENA | 9.82 | 11.14 | 1.13 | 0.95 | Female |
| 70 | Austria | NA | 14.86 | 17.5 | 1.18 | 0.99 | None |
| 70 | Belgium | NA | 15 | 17.68 | 1.18 | 0.99 | None |
| 70 | Canada | NA | 15.89 | 18.26 | 1.15 | 0.97 | Female |
| 70 | Cyprus | NA | 13.79 | 16.46 | 1.19 | 1 | None |
| 70 | Denmark | NA | 14.69 | 17 | 1.16 | 0.97 | None |
| 70 | Finland | NA | 14.85 | 17.82 | 1.2 | 1.01 | None |
| 70 | France | NA | 15.9 | 19.16 | 1.2 | 1.01 | None |
| 70 | Germany | NA | 14.54 | 17.15 | 1.18 | 0.99 | None |
| 70 | Greece | NA | 14.49 | 16.91 | 1.17 | 0.98 | None |
| 70 | Iceland | NA | 15.88 | 17.47 | 1.1 | 0.93 | Female |
| 70 | Ireland | NA | 15.26 | 17.69 | 1.16 | 0.98 | None |
| 70 | Italy | NA | 15.5 | 18.21 | 1.18 | 0.99 | None |
| 70 | Luxembourg | NA | 15.16 | 17.73 | 1.17 | 0.98 | None |
| 70 | Malta | NA | 15.17 | 18.76 | 1.24 | 1.04 | Male |
| 70 | Netherlands | NA | 14.95 | 17.12 | 1.15 | 0.96 | Female |
| 70 | Norway | NA | 15.53 | 17.66 | 1.14 | 0.96 | Female |
| 70 | Portugal | NA | 15.04 | 17.93 | 1.19 | 1 | None |
| 70 | Spain | NA | 15.61 | 18.95 | 1.21 | 1.02 | None |
| 70 | Sweden | NA | 15.59 | 17.75 | 1.14 | 0.96 | Female |
| 70 | Switzerland | NA | 16.05 | 18.36 | 1.14 | 0.96 | Female |
| 70 | United Kingdom | NA | 15.16 | 17.24 | 1.14 | 0.96 | Female |
| 70 | Angola | SSA | 9.59 | 10.86 | 1.13 | 0.95 | Female |
| 70 | Benin | SSA | 10.02 | 10.57 | 1.06 | 0.89 | Female |
| 70 | Botswana | SSA | 10.45 | 12.52 | 1.2 | 1.01 | None |
| 70 | Burkina Faso | SSA | 8.72 | 9.54 | 1.09 | 0.92 | Female |
| 70 | Burundi | SSA | 9.94 | 10.78 | 1.08 | 0.91 | Female |
| 70 | Cabo Verde | SSA | 11.13 | 13.54 | 1.22 | 1.02 | None |
| 70 | Cameroon | SSA | 9.46 | 10.36 | 1.1 | 0.92 | Female |
| 70 | Central African Republic | SSA | 5.92 | 7.07 | 1.2 | 1.01 | None |
| 70 | Chad | SSA | 8.86 | 9.6 | 1.08 | 0.91 | Female |
| 70 | Comoros | SSA | 9.24 | 10.61 | 1.15 | 0.97 | Female |
| 70 | Congo | SSA | 9.53 | 10.47 | 1.1 | 0.93 | Female |
| 70 | Côte D'ivoire | SSA | 9.65 | 11.1 | 1.15 | 0.97 | Female |
| 70 | Democratic Republic Of The Congo | SSA | 9.95 | 10.75 | 1.08 | 0.91 | Female |
| 70 | Djibouti | SSA | 10.29 | 11.4 | 1.11 | 0.93 | Female |
| 70 | Equatorial Guinea | SSA | 9.73 | 10.62 | 1.09 | 0.92 | Female |
| 70 | Eritrea | SSA | 10.75 | 11.74 | 1.09 | 0.92 | Female |
| 70 | Eswatini | SSA | 10.47 | 11.7 | 1.12 | 0.94 | Female |
| 70 | Ethiopia | SSA | 10.41 | 11.79 | 1.13 | 0.95 | Female |
| 70 | Gabon | SSA | 10.65 | 11.9 | 1.12 | 0.94 | Female |
| 70 | Gambia | SSA | 10.4 | 11.09 | 1.07 | 0.9 | Female |
| 70 | Ghana | SSA | 10.23 | 11.19 | 1.09 | 0.92 | Female |
| 70 | Guinea | SSA | 10.13 | 10.64 | 1.05 | 0.88 | Female |
| 70 | Guinea-Bissau | SSA | 9.73 | 10.84 | 1.11 | 0.94 | Female |
| 70 | Kenya | SSA | 10.37 | 11.98 | 1.16 | 0.97 | None |
| 70 | Lesotho | SSA | 9.08 | 11.19 | 1.23 | 1.04 | Male |
| 70 | Liberia | SSA | 9.4 | 9.55 | 1.02 | 0.85 | Female |
| 70 | Madagascar | SSA | 10.41 | 11.14 | 1.07 | 0.9 | Female |
| 70 | Malawi | SSA | 9.69 | 11.74 | 1.21 | 1.02 | None |
| 70 | Mali | SSA | 8.87 | 9.41 | 1.06 | 0.89 | Female |
| 70 | Mauritania | SSA | 9.56 | 10.99 | 1.15 | 0.97 | Female |
| 70 | Mauritius | SSA | 12.73 | 14.68 | 1.15 | 0.97 | None |
| 70 | Mozambique | SSA | 8.88 | 9.62 | 1.08 | 0.91 | Female |
| 70 | Namibia | SSA | 9.69 | 12.94 | 1.33 | 1.12 | Male |
| 70 | Niger | SSA | 9.27 | 9.85 | 1.06 | 0.89 | Female |
| 70 | Nigeria | SSA | 9.14 | 9.35 | 1.02 | 0.86 | Female |
| 70 | Rwanda | SSA | 10.02 | 11.19 | 1.12 | 0.94 | Female |
| 70 | Senegal | SSA | 9.57 | 10.56 | 1.1 | 0.93 | Female |
| 70 | Sierra Leone | SSA | 9.05 | 9.79 | 1.08 | 0.91 | Female |
| 70 | Somalia | SSA | 9.83 | 10.62 | 1.08 | 0.91 | Female |
| 70 | South Africa | SSA | 11.53 | 13.99 | 1.21 | 1.02 | None |
| 70 | South Sudan | SSA | 9.57 | 10.61 | 1.11 | 0.93 | Female |
| 70 | Sudan | SSA | 10.57 | 11.83 | 1.12 | 0.94 | Female |
| 70 | Togo | SSA | 8.93 | 9.08 | 1.02 | 0.86 | Female |
| 70 | Uganda | SSA | 11.32 | 14.19 | 1.25 | 1.06 | Male |
| 70 | United Republic Of Tanzania | SSA | 10.11 | 13.04 | 1.29 | 1.09 | Male |
| 70 | Zambia | SSA | 8.81 | 10.08 | 1.14 | 0.96 | Female |
| 70 | Zimbabwe | SSA | 9.4 | 11.37 | 1.21 | 1.02 | None |
| 70 | United States Of America | CIU | 14.86 | 16.96 | 1.14 | 0.96 | Female |
| 70 | Australia | WPSA | 16.3 | 18.51 | 1.14 | 0.96 | Female |
| 70 | Bangladesh | WPSA | 12.12 | 13.82 | 1.14 | 0.96 | Female |
| 70 | Bhutan | WPSA | 10.52 | 11.93 | 1.13 | 0.96 | Female |
| 70 | Brunei Darussalam | WPSA | 11.55 | 13.77 | 1.19 | 1 | None |
| 70 | Cambodia | WPSA | 11.05 | 12.27 | 1.11 | 0.93 | Female |
| 70 | Dem. People's Republic Of Korea | WPSA | 10.89 | 13.07 | 1.2 | 1.01 | None |
| 70 | Fiji | WPSA | 8.37 | 10.3 | 1.23 | 1.04 | Male |
| 70 | Indonesia | WPSA | 10.02 | 11.88 | 1.19 | 1 | None |
| 70 | Japan | WPSA | 15.91 | 20.19 | 1.27 | 1.07 | Male |
| 70 | Lao People's Democratic Republic | WPSA | 9.81 | 11.05 | 1.13 | 0.95 | Female |
| 70 | Malaysia | WPSA | 12.73 | 14.59 | 1.15 | 0.96 | Female |
| 70 | Maldives | WPSA | 12.91 | 14.95 | 1.16 | 0.98 | None |
| 70 | Myanmar | WPSA | 9.45 | 10.9 | 1.15 | 0.97 | None |
| 70 | Nepal | WPSA | 10 | 10.99 | 1.1 | 0.92 | Female |
| 70 | New Zealand | WPSA | 15.52 | 17.53 | 1.13 | 0.95 | Female |
| 70 | Papua New Guinea | WPSA | 9.02 | 10.79 | 1.2 | 1.01 | None |
| 70 | Philippines | WPSA | 9.78 | 11.85 | 1.21 | 1.02 | None |
| 70 | Republic Of Korea | WPSA | 14.64 | 18.89 | 1.29 | 1.09 | Male |
| 70 | Singapore | WPSA | 15.71 | 18.57 | 1.18 | 1 | None |
| 70 | Solomon Islands | WPSA | 10.01 | 10.96 | 1.09 | 0.92 | Female |
| 70 | Sri Lanka | WPSA | 11.68 | 14.4 | 1.23 | 1.04 | Male |
| 70 | Taiwan | WPSA | 14.54 | 17.85 | 1.23 | 1.03 | Male |
| 70 | Thailand | WPSA | 14.6 | 17.86 | 1.22 | 1.03 | Male |
| 70 | Timor-Leste | WPSA | 9.75 | 10.81 | 1.11 | 0.93 | Female |
| 70 | Vanuatu | WPSA | 10.2 | 12.18 | 1.19 | 1.01 | None |
| 70 | Viet Nam | WPSA | 11.41 | 15.33 | 1.34 | 1.13 | Male |

## **Appendix Table D.**Alternative frontier classification, >5 million population countries

| Age | Frontier life expectancy, female | Frontier life expectancy, male | Frontier sex ratio | Top 5% countries, male | Top 5% countries, female |
| --- | --- | --- | --- | --- | --- |
| 0 | 85.56 | 81.21 | 1.053 | United Arab Emirates, Switzerland, Hong Kong, Japan, Singapore, Sweden | Spain, France, Hong Kong, Japan, Republic of Korea, Singapore |
| 5 | 80.87 | 76.55 | 1.056 | United Arab Emirates, Switzerland, Hong Kong, Japan, Singapore, Sweden | Spain, France, Hong Kong, Japan, Republic of Korea, Singapore |
| 15 | 70.92 | 66.60 | 1.065 | United Arab Emirates, Australia, Switzerland, Hong Kong, Japan, Singapore | Spain, France, Hong Kong, Japan, Republic Of Korea, Singapore |
| 35 | 51.13 | 47.09 | 1.086 | United Arab Emirates, Australia, Switzerland, Hong Kong, Japan, Sweden | Spain, France, Hong Kong, Japan, Republic of Korea, Singapore |
| 50 | 36.52 | 32.77 | 1.114 | United Arab Emirates, Australia, Switzerland, Hong Kong, Japan, Sweden | Spain, France, Hong Kong, Japan, Republic of Korea, Singapore |
| 70 | 18.52 | 15.89 | 1.165 | Australia, Switzerland, France, Hong Kong, Israel, Japan | Spain, France, Hong Kong, Japan, Republic of Korea, Singapore |

## **Appendix Table E.** Number of countries with equal sex ratio or female/male disadvantage in life expectancy at different ages, removing countries not listed in the United Nations regions

|  | Original sex ratio | | | Adjusted sex ratio | | |
| --- | --- | --- | --- | --- | --- | --- |
|  | (A) Female disadvantage | (B) Male disadvantage | (C) No disadvantage | (A) Female disadvantage | (B) Male disadvantage | (C) No disadvantage |
| Birth | 0 | 173 | 0 | 28 | 118 | 27 |
| Age 5 | 2 | 173 | 0 | 39 | 100 | 32 |
| Age 15 | 0 | 173 | 0 | 37 | 103 | 33 |
| Age 35 | 0 | 173 | 0 | 40 | 93 | 40 |
| Age 50 | 0 | 173 | 0 | 54 | 82 | 37 |
| Age 70 | 0 | 173 | 0 | 74 | 45 | 54 |

## **Appendix Table F.**Buffer definitions and life expectancies

| Age | 30th percentile | | | 10% of adjusted ratio | | | 1 year LE increase | | | 0.5 year LE increase | | |
| --- | --- | --- | --- | --- | --- | --- | --- | --- | --- | --- | --- | --- |
|  | Low | High | % in buffer | Low | High | % in buffer | Low | High | % in buffer | Low | High | % in buffer |
| 0 | 0.99 | 1.011 | 30% | 0.99 | 1.01 | 28% | 0.988 | 1.012 | 35% | 0.994 | 1.006 | 17% |
| 5 | 0.99 | 1.01 | 30% | 0.99 | 1.01 | 32% | 0.987 | 1.012 | 37% | 0.994 | 1.006 | 20% |
| 15 | 0.99 | 1.012 | 30% | 0.99 | 1.01 | 27% | 0.985 | 1.014 | 38% | 0.993 | 1.007 | 21% |
| 35 | 0.986 | 1.013 | 30% | 0.99 | 1.01 | 24% | 0.979 | 1.019 | 40% | 0.990 | 1.010 | 24% |
| 50 | 0.982 | 1.021 | 30% | 0.99 | 1.01 | 16% | 0.97 | 1.027 | 44% | 0.985 | 1.014 | 22% |
| 70 | 0.97 | 1.021 | 30% | 0.99 | 1.01 | 14% | 0.941 | 1.053 | 61% | 0.969 | 1.027 | 34% |

## **Appendix Figure A.** Distribution of differences and adjusted differences in 2019, by age

**
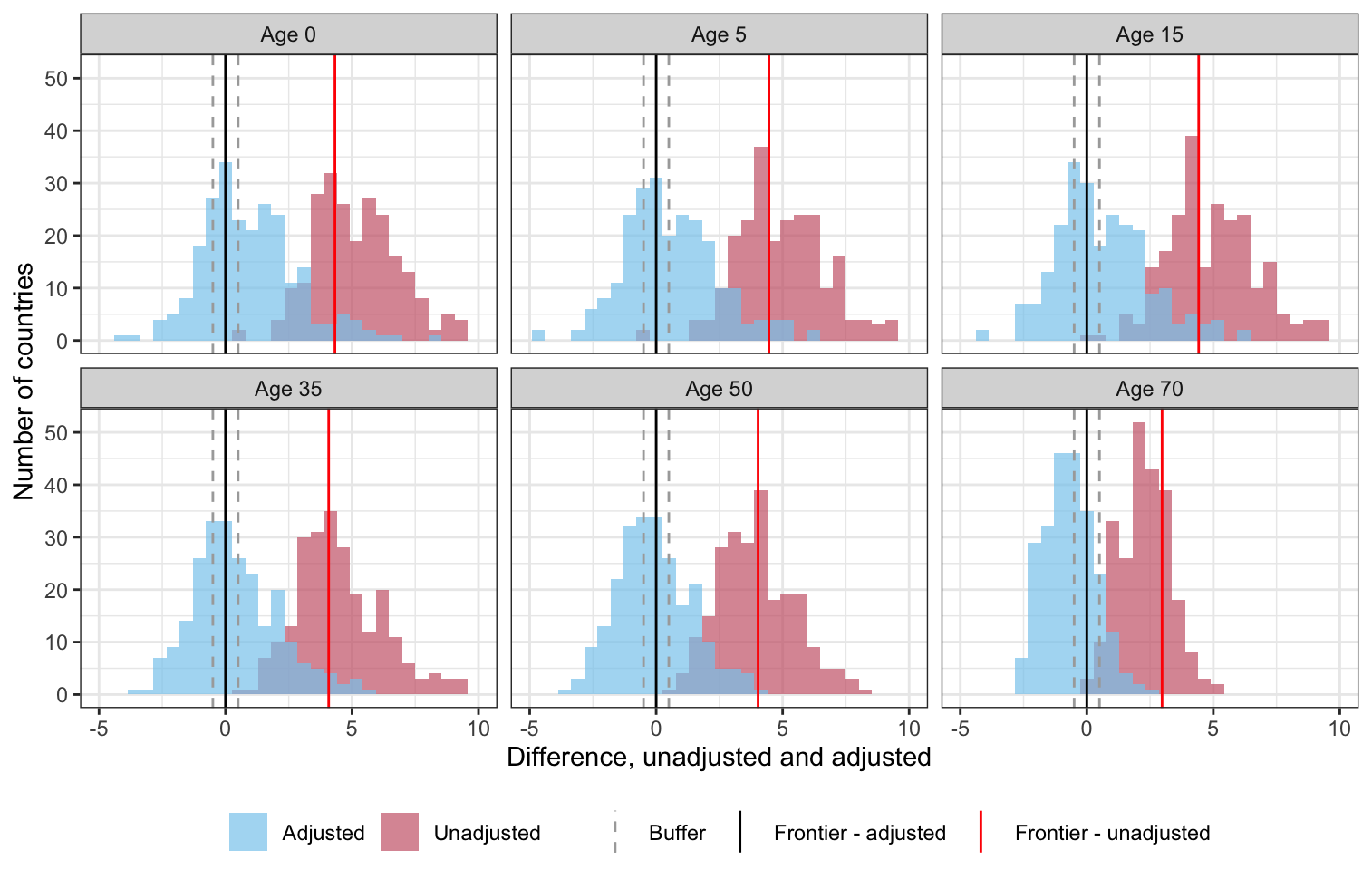
**

## **Appendix Figure B.** Adjusted differences by life expectancy for the 30 most populous countries, 2019, by age

**
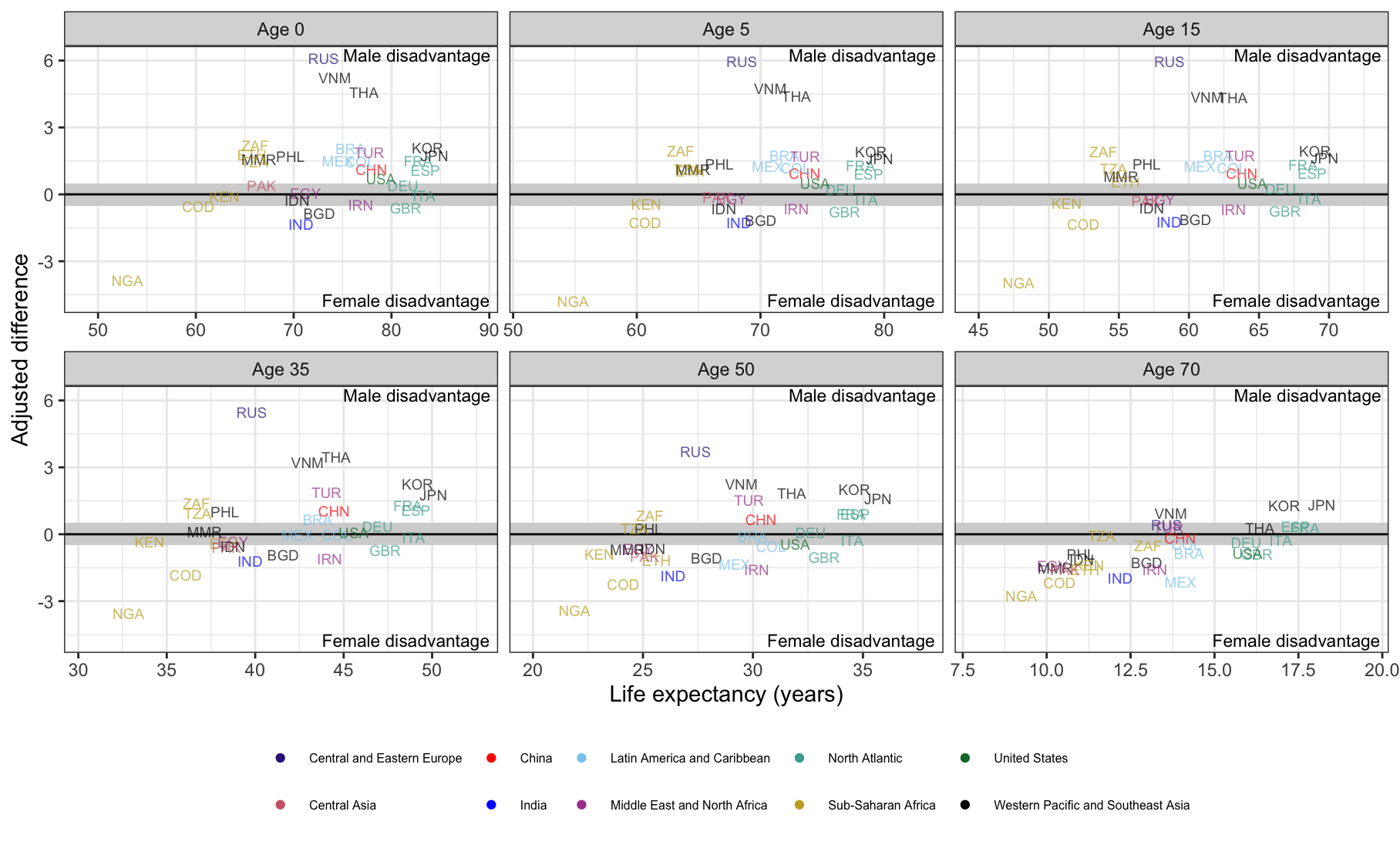
**

## **Appendix Figure C.** Adjusted differences by CIH regions, 2019, by age


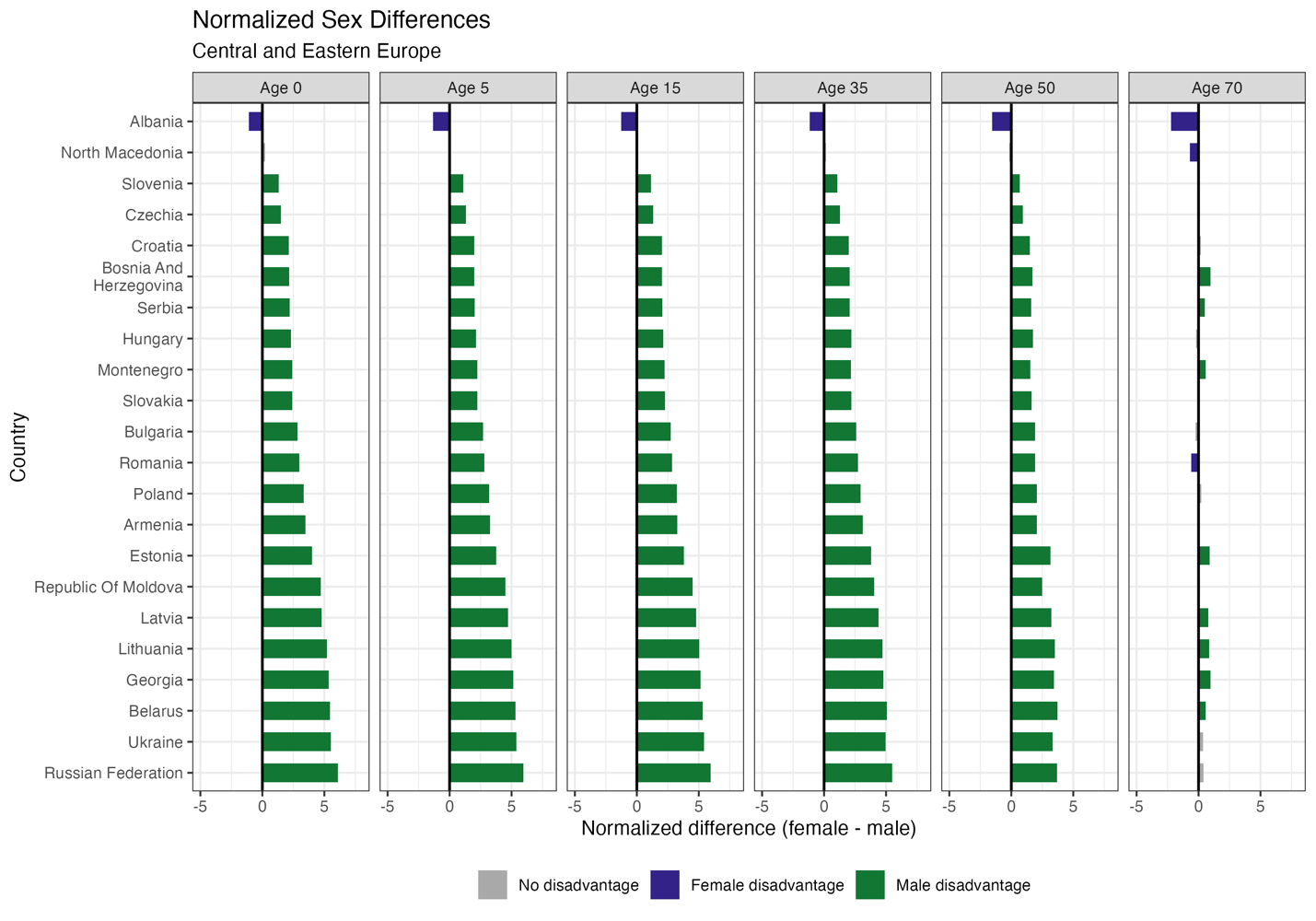


**
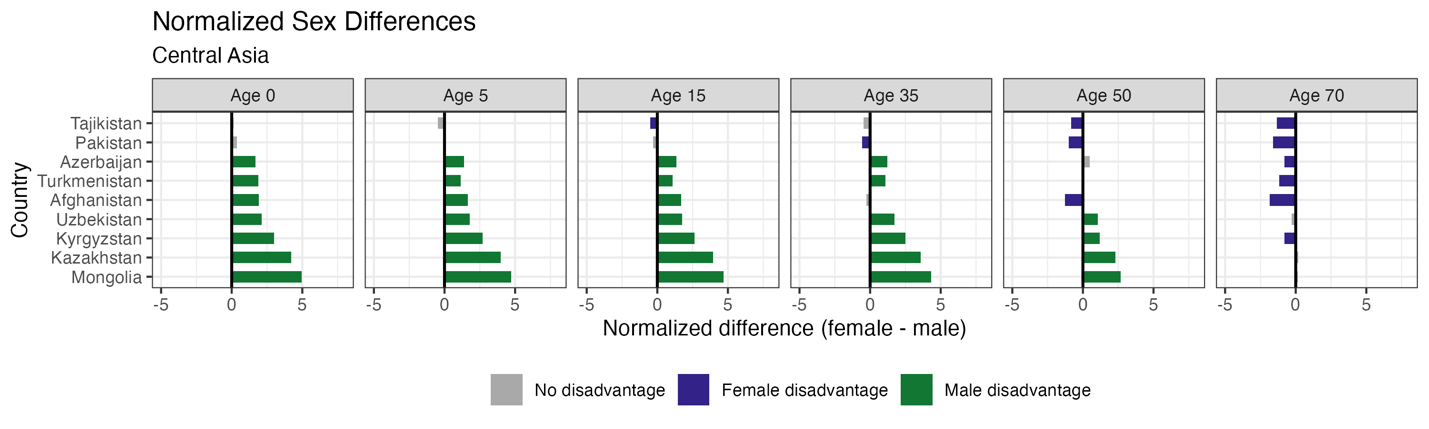
**

**
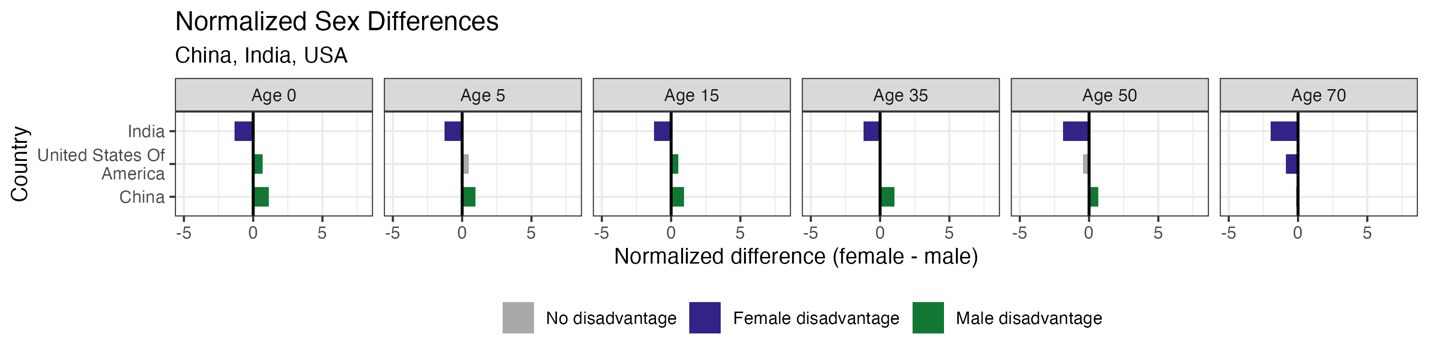
**

**
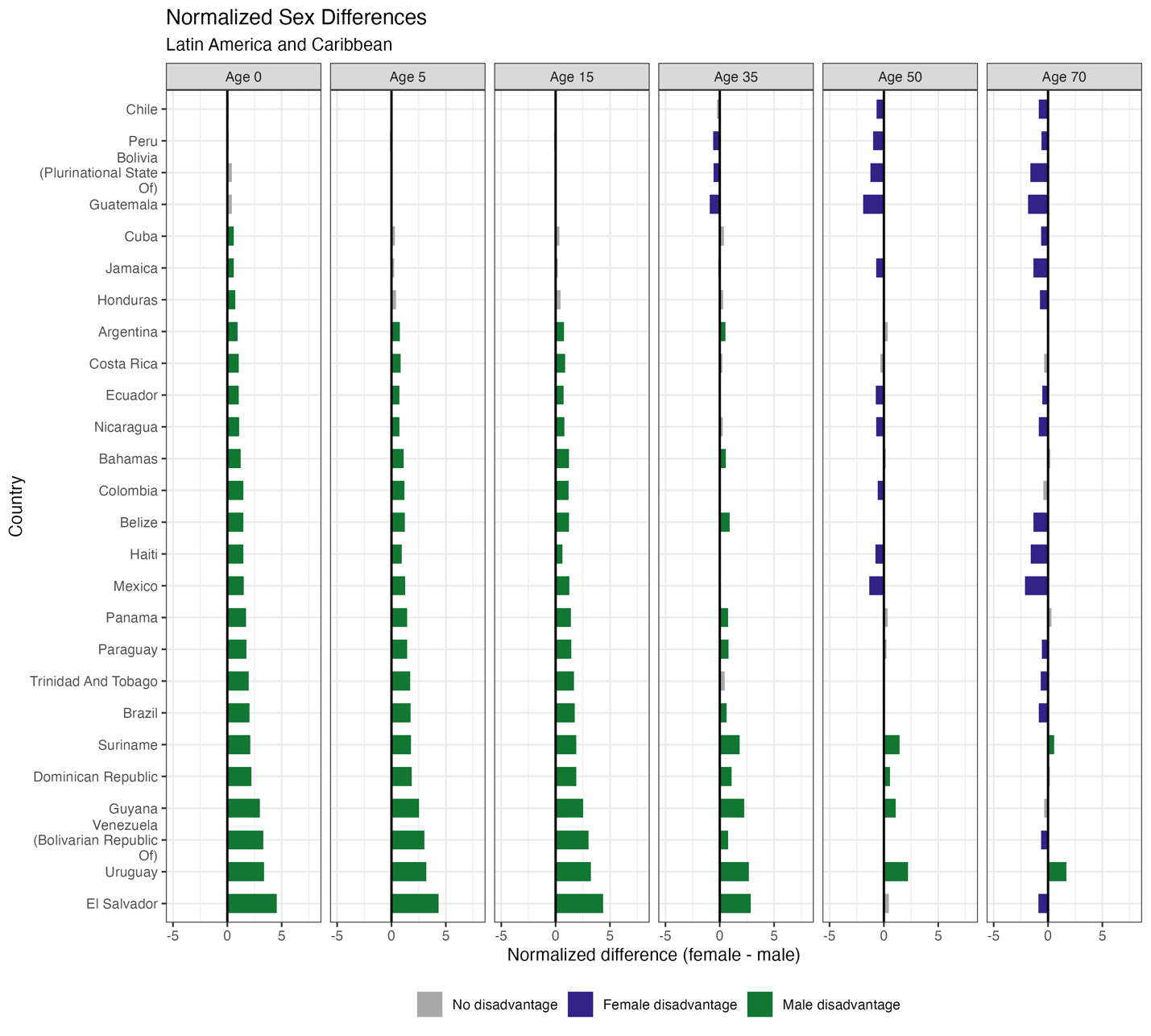
**

**
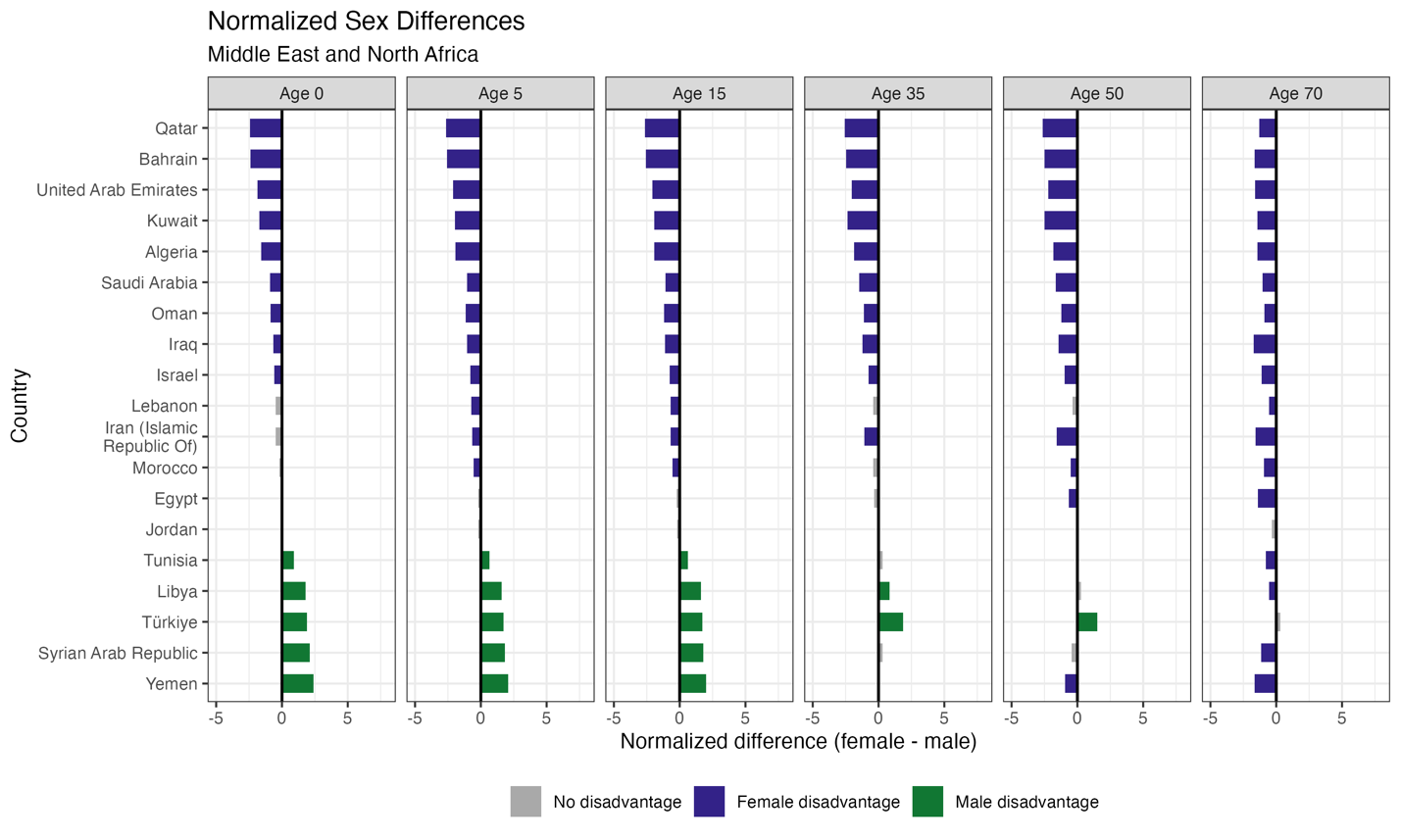
**

**
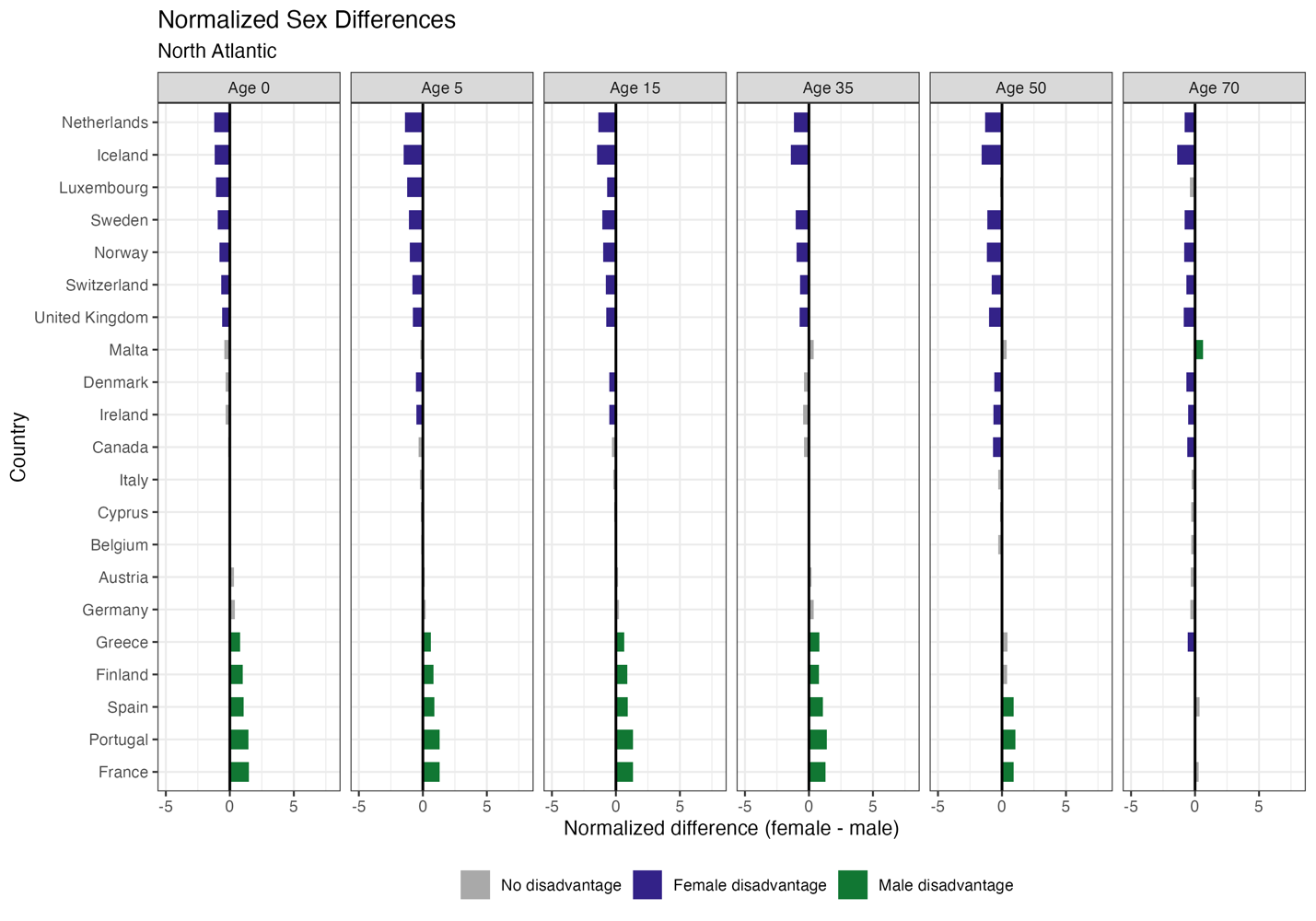
**

**
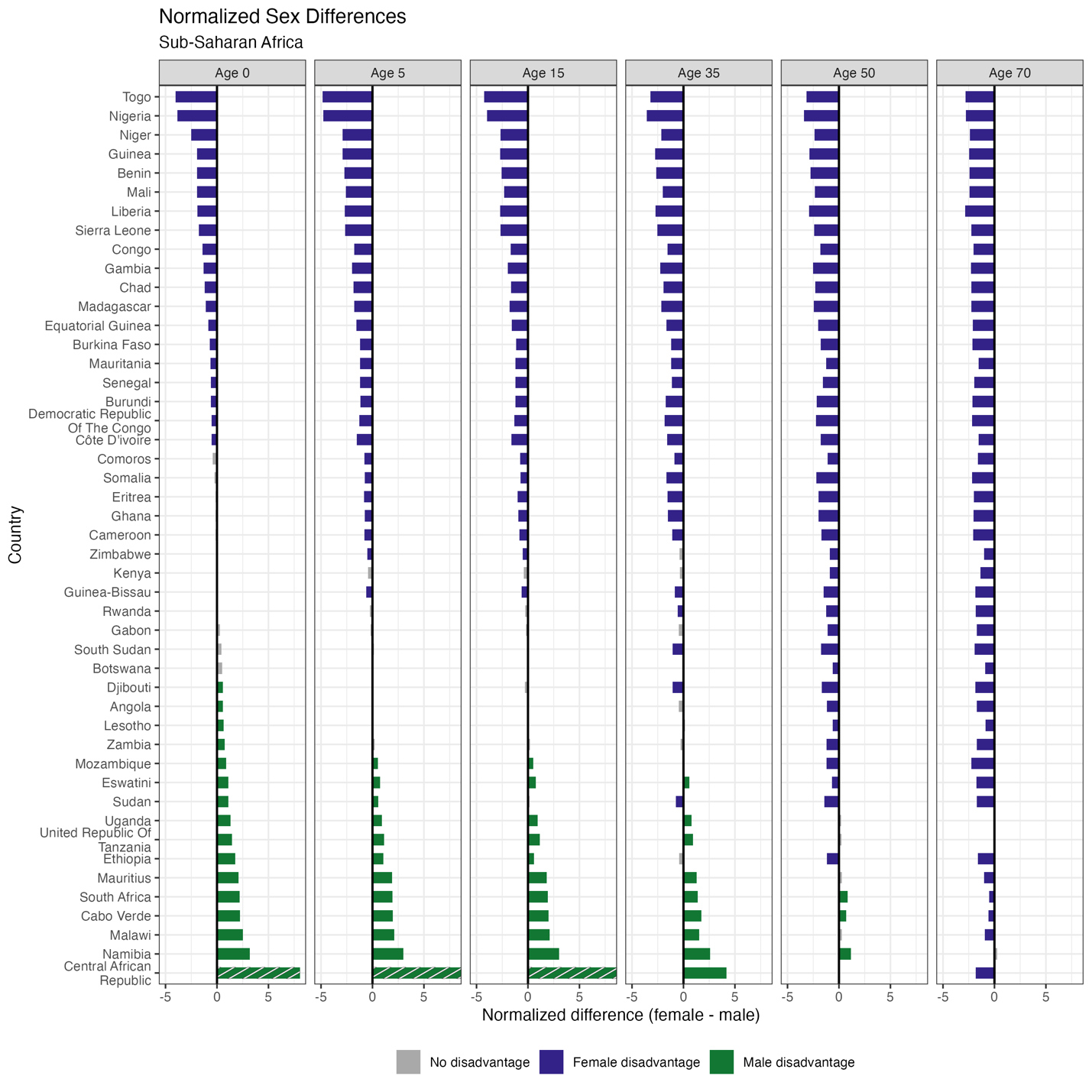
**

**
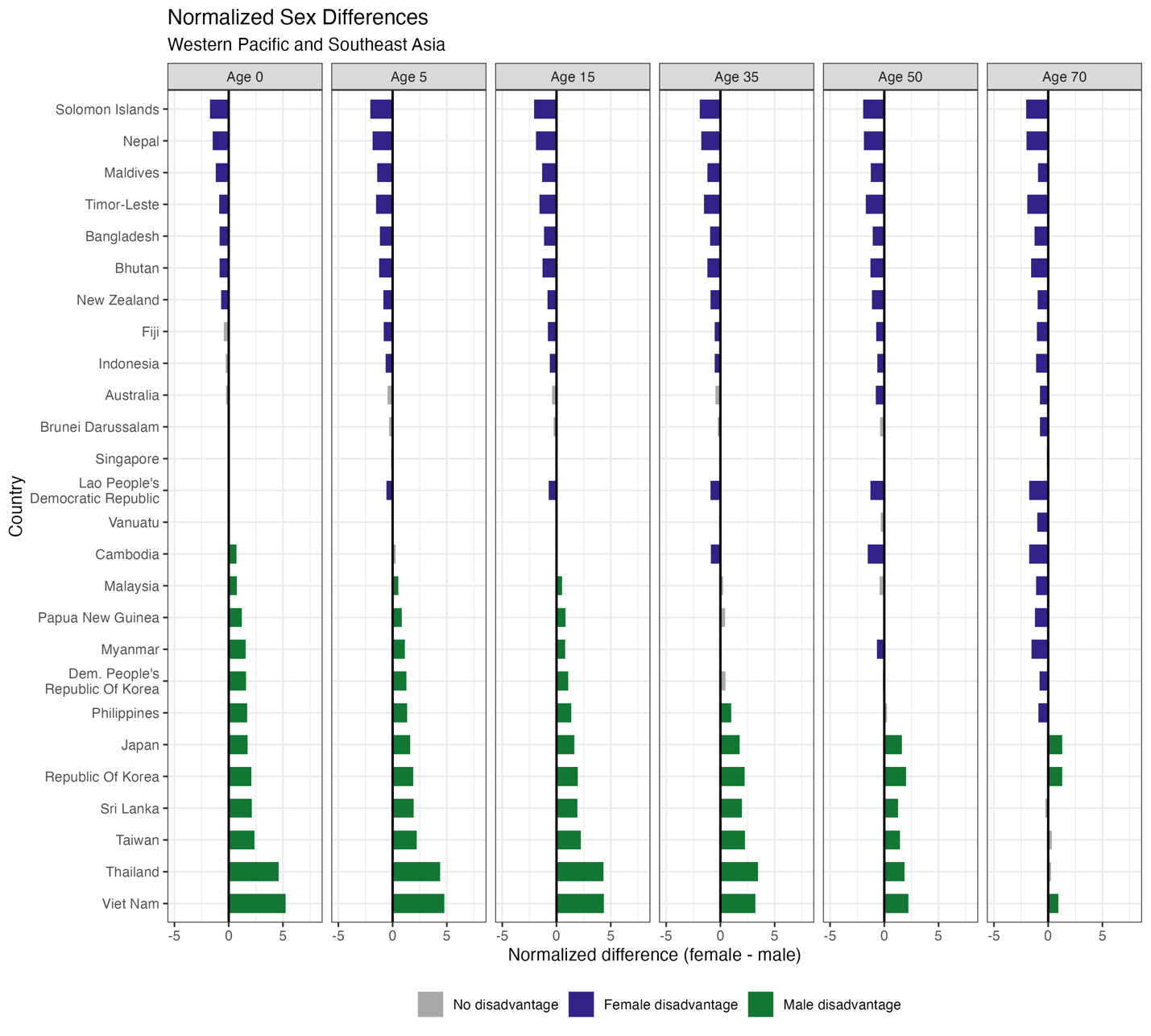
**

## **Appendix Figure D.** Comparison of adjusted ratios and adjusted differences, by age


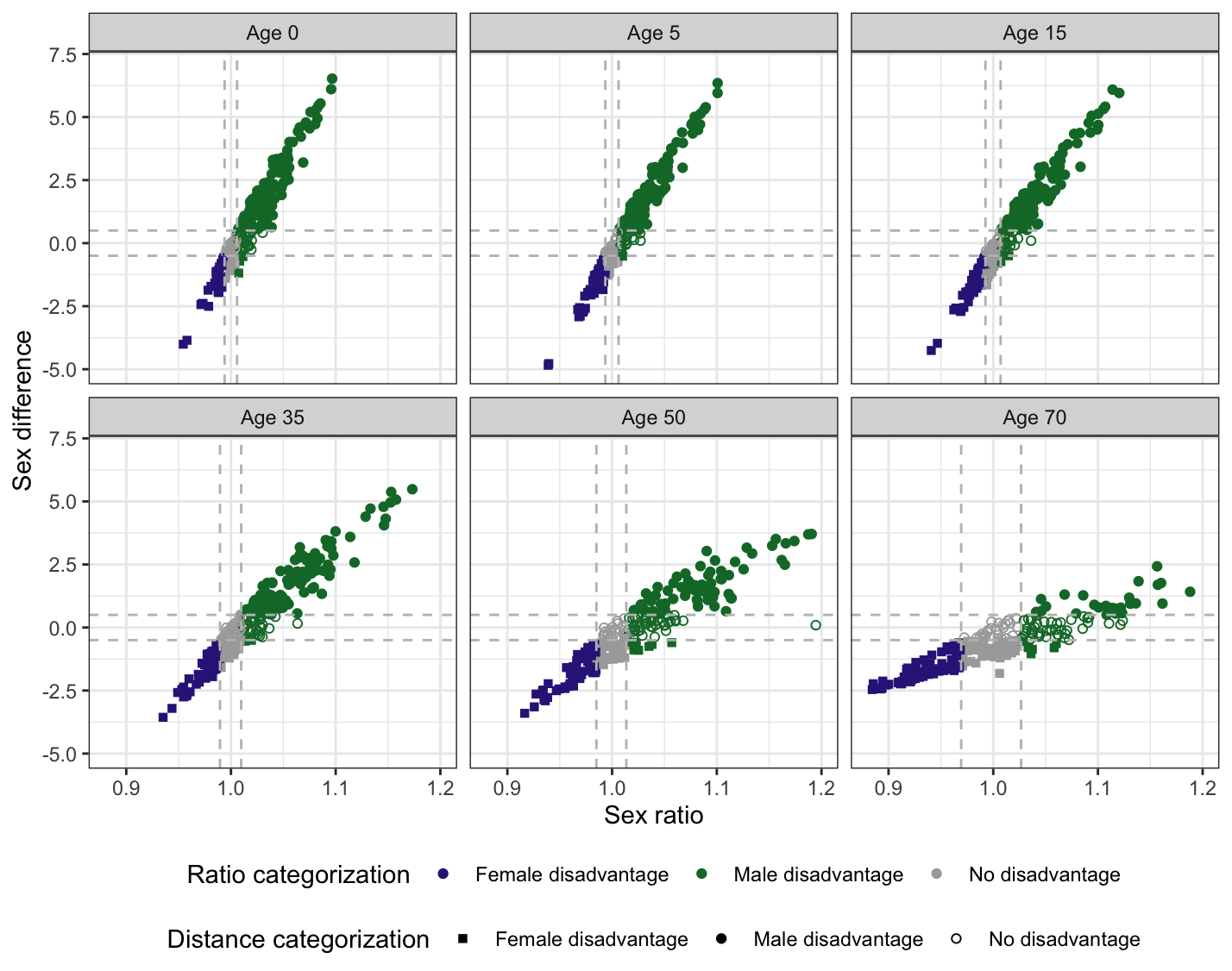


The gray dashed lines indicate the half-year life expectancy increase buffer.

## **Appendix Figure E.** Frontier ratio parameter sensitivity

**
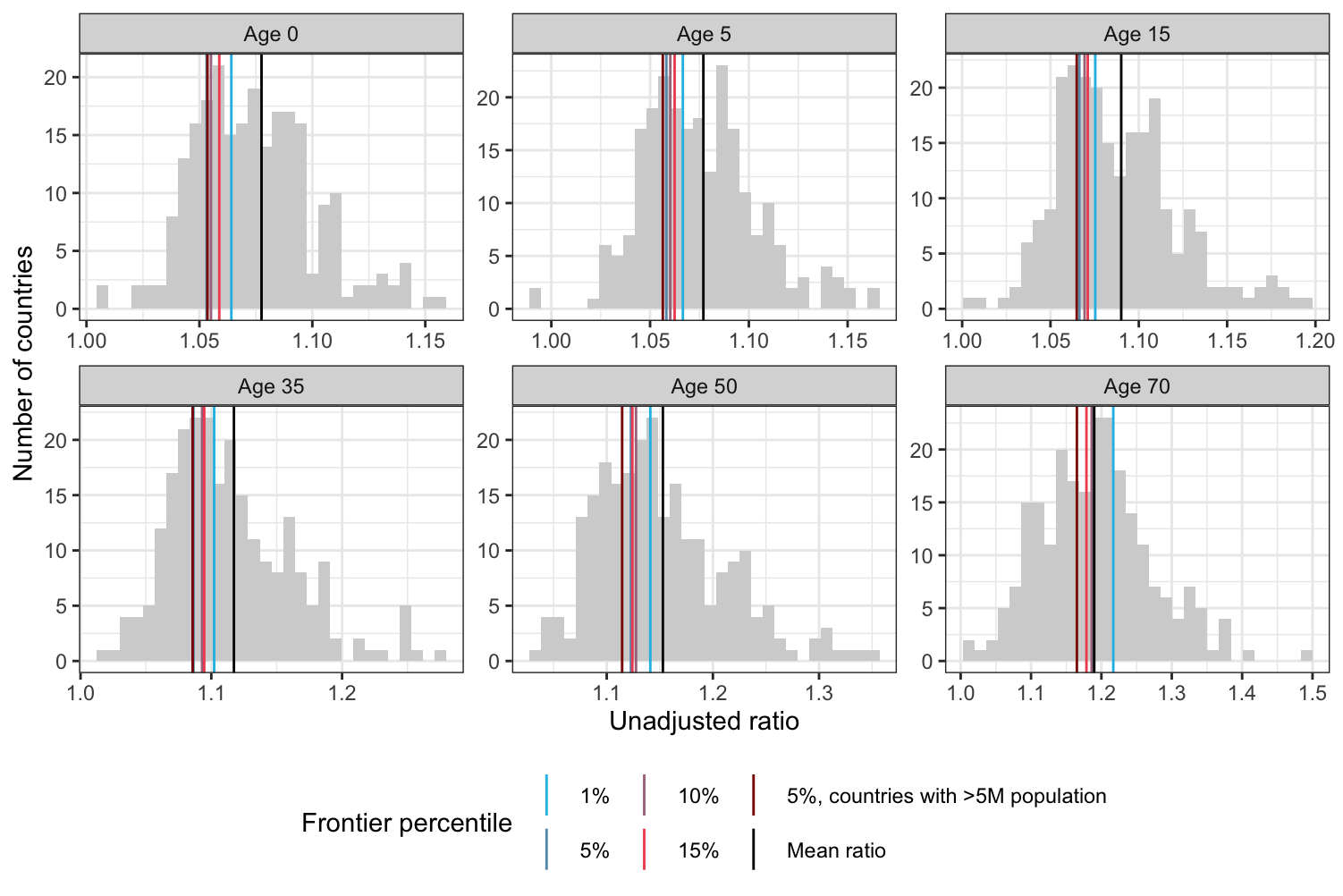
**

## **Appendix Figure F.** Country classification with various buffer methods, age 0 and 70


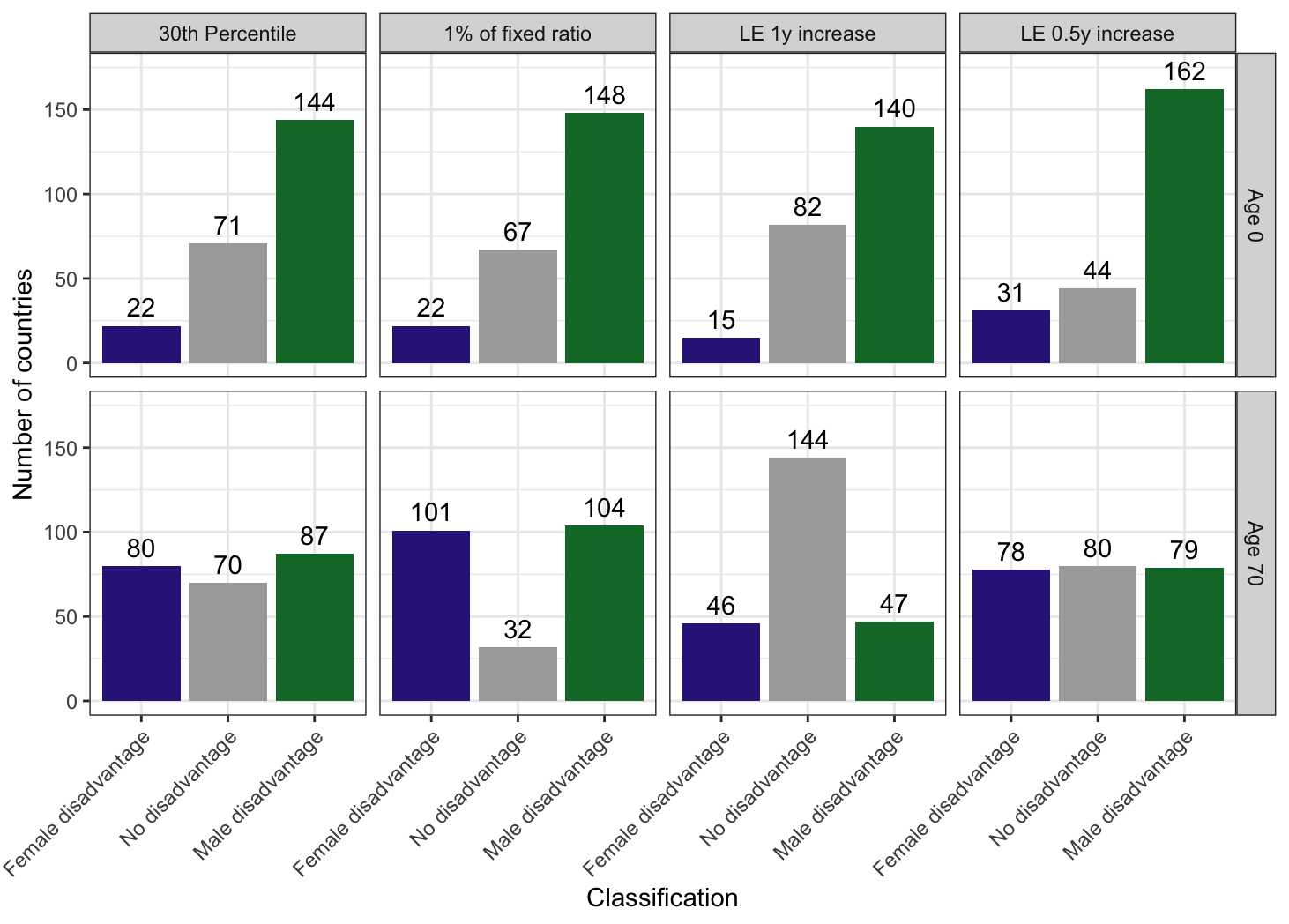

Supplement: S1 Appendix — Appendix Table A. Adjusted ratio buffer boundaries, by age, 2019. Appendix Table B. Number of countries and proportion of world population classified as female disadvantage, male disadvantage, or no disadvantage before and after adjustment, by life expectancy at different ages, 2019. Appendix Table C. Adjusted sex ratios, unadjusted sex ratios, and life expectancy by sex at each age by country, 2019. Appendix Table D. Alternative frontier classification, >5 million population countries. Appendix Table E. Number of countries with equal sex ratio or female/male disadvantage in life expectancy at different ages, removing countries not listed in the United Nations regions. Appendix Table F. Buffer definitions and life expectancies. Appendix Figure A. Distribution of differences and adjusted differences in 2019, by age. Appendix Figure B. Adjusted differences by life expectancy for the 30 most populous countries, 2019, by age. Appendix Figure C. Adjusted differences by CIH regions, 2019, by age. Appendix Figure D. Comparison of adjusted ratios and adjusted differences, by age. Appendix Figure E. Frontier ratio parameter sensitivity. Appendix Figure F. Country classification with various buffer methods, age 0 and 70. (DOCX) [file pmed.1004828.s001.docx]
